# A dehydrated space-weathered skin cloaking the hydrated interior of Ryugu

**DOI:** 10.1038/s41550-022-01841-6

**Published:** 2022-12-19

**Authors:** Takaaki Noguchi, Toru Matsumoto, Akira Miyake, Yohei Igami, Mitsutaka Haruta, Hikaru Saito, Satoshi Hata, Yusuke Seto, Masaaki Miyahara, Naotaka Tomioka, Hope A. Ishii, John P. Bradley, Kenta K. Ohtaki, Elena Dobrică, Hugues Leroux, Corentin Le Guillou, Damien Jacob, Francisco de la Peña, Sylvain Laforet, Maya Marinova, Falko Langenhorst, Dennis Harries, Pierre Beck, Thi H. V. Phan, Rolando Rebois, Neyda M. Abreu, Jennifer Gray, Thomas Zega, Pierre-M. Zanetta, Michelle S. Thompson, Rhonda Stroud, Kate Burgess, Brittany A. Cymes, John C. Bridges, Leon Hicks, Martin R. Lee, Luke Daly, Phil A. Bland, Michael E. Zolensky, David R. Frank, James Martinez, Akira Tsuchiyama, Masahiro Yasutake, Junya Matsuno, Shota Okumura, Itaru Mitsukawa, Kentaro Uesugi, Masayuki Uesugi, Akihisa Takeuchi, Mingqi Sun, Satomi Enju, Aki Takigawa, Tatsuhiro Michikami, Tomoki Nakamura, Megumi Matsumoto, Yusuke Nakauchi, Masanao Abe, Masahiko Arakawa, Atsushi Fujii, Masahiko Hayakawa, Naru Hirata, Naoyuki Hirata, Rie Honda, Chikatoshi Honda, Satoshi Hosoda, Yu-ichi Iijima, Hitoshi Ikeda, Masateru Ishiguro, Yoshiaki Ishihara, Takahiro Iwata, Kousuke Kawahara, Shota Kikuchi, Kohei Kitazato, Koji Matsumoto, Moe Matsuoka, Yuya Mimasu, Akira Miura, Tomokatsu Morota, Satoru Nakazawa, Noriyuki Namiki, Hirotomo Noda, Rina Noguchi, Naoko Ogawa, Kazunori Ogawa, Tatsuaki Okada, Chisato Okamoto, Go Ono, Masanobu Ozaki, Takanao Saiki, Naoya Sakatani, Hirotaka Sawada, Hiroki Senshu, Yuri Shimaki, Kei Shirai, Seiji Sugita, Yuto Takei, Hiroshi Takeuchi, Satoshi Tanaka, Eri Tatsumi, Fuyuto Terui, Ryudo Tsukizaki, Koji Wada, Manabu Yamada, Tetsuya Yamada, Yukio Yamamoto, Hajime Yano, Yasuhiro Yokota, Keisuke Yoshihara, Makoto Yoshikawa, Kent Yoshikawa, Ryohta Fukai, Shizuho Furuya, Kentaro Hatakeda, Tasuku Hayashi, Yuya Hitomi, Kazuya Kumagai, Akiko Miyazaki, Aiko Nakato, Masahiro Nishimura, Hiromichi Soejima, Ayako I. Suzuki, Tomohiro Usui, Toru Yada, Daiki Yamamoto, Kasumi Yogata, Miwa Yoshitake, Harold C. Connolly, Dante S. Lauretta, Hisayoshi Yurimoto, Kazuhide Nagashima, Noriyuki Kawasaki, Naoya Sakamoto, Ryuji Okazaki, Hikaru Yabuta, Hiroshi Naraoka, Kanako Sakamoto, Shogo Tachibana, Sei-ichiro Watanabe, Yuichi Tsuda

**Affiliations:** 1 Division of Earth and Planetary Sciences, Kyoto University, Kyoto, Japan; 2 The Hakubi Center for Advanced Research, Kyoto University, Kyoto, Japan; 3 Institute for Chemical Research, Kyoto University, Kyoto, Japan; 4 Institute for Materials Chemistry and Engineering, Kyushu University, Fukuoka, Japan; 5 Pan-Omics Data-Driven Research Innovation Center, Kyushu University, Fukuoka, Japan; 6 Interdisciplinary Graduate School of Engineering Sciences, Kyushu University, Fukuoka, Japan; 7 The Ultramicroscopy Research Center, Kyushu University, Fukuoka, Japan; 8 Department of Geosciences, Osaka Metropolitan University, Osaka, Japan; 9 Department of Earth and Planetary Systems Science, Hiroshima University, Hiroshima, Japan; 10 Kochi Institute for Core Sample Research, X-Star, JAMSTEC, Nankoku, Japan; 11 Hawai’i Institute of Geophysics and Planetology, The University of Hawai’i at Mānoa, Honolulu, HI USA; 12 Unité Matériaux et Transformations UMR 8207, Université de Lille, CNRS, INRAE, Centrale Lille, Lille, France; 13 Institut Michel-Eugène Chevreul FR 2638, Université de Lille, CNRS, INRAE, Centrale Lille, Université Artois, Lille, France; 14 Institut für Geowissenschaften, Friedrich-Schiller-Universität Jena, Jena, Germany; 15 European Space Resources Innovation Centre, Luxembourg Institute of Science and Technology, Belvaux, Luxembourg; 16 Institut de Planétologie et d’Astrophysique de Grenoble (IPAG), Université Grenoble Alpes, CNRS, Grenoble, France; 17 NASA Langley Research Center, Hampton, VA USA; 18 Materials Characterization Lab, The Pennsylvania State University Materials Research Institute, University Park, USA; 19 Lunar and Planetary Laboratory, The University of Arizona, Tucson, AZ USA; 20 Department of Earth, Atmospheric and Planetary Sciences, Purdue University, West Lafayette, IN USA; 21 Buseck Center for Meteorite Studies, Arizona State University, Tempe, AZ USA; 22 Materials Science and Technology Division, US Naval Research Laboratory, Washington, DC USA; 23 NRC Postdoctoral Research Associate, US Naval Research Laboratory, Washington, DC USA; 24 Space Park Leichester, The University of Leicester, Leicester, UK; 25 School of Geology, Geography and the Environment, The University of Leicester, Leicester, UK; 26 School of Geographical and Earth Sciences, The University of Glasgow, Glasgow, UK; 27 Australian Centre for Microscopy and Microanalysis, The University of Sydney, Sydney, New South Wales Australia; 28 Department of Materials, The University of Oxford, Oxford, UK; 29 School of Earth and Planetary Sciences, Curtin University, Perth, Western Australia Australia; 30 ARES, NASA Johnson Space Center, Houston, TX USA; 31 Jacobs Engineering, Dallas, TX USA; 32 Research Organization of Science and Technology, Ritsumeikan University, Kusatsu, Japan; 33 CAS Key Laboratory of Mineralogy and Metallogeny, Guangdong Provincial Key Laboratory of Mineral Physics and Materials, Guangzhou Institute of Geochemistry, Chinese Academy of Sciences (CAS), Guangzhou, China; 34 CAS Center for Excellence in Deep Earth Science, Guangzhou, China; 35 Japan Synchrotron Radiation Research Institute (JASRI/SPring-8), Sayo, Japan; 36 University of Chinese Academy of Sciences, Beijing, China; 37 Department of Mathematics, Physics, and Earth Science, Ehime University, Matsuyama, Japan; 38 Department of Earth and Planetary Science, The University of Tokyo, Tokyo, Japan; 39 Faculty of Engineering, Kindai University, Higashi-Hiroshima, Japan; 40 Department of Earth Science, Tohoku University, Sendai, Japan; 41 Institute of Space and Astronautical Science, Japan Aerospace Exploration Agency, Sagamihara, Japan; 42 The Graduate University for Advanced Studies (SOKENDAI), Hayama, Japan; 43 Department of Planetology, Kobe University, Kobe, Japan; 44 Aizu Research Center for Space Informatics, The University of Aizu, Fukushima, Japan; 45 Department of Information Science, Kochi University, Kochi, Japan; 46 Department of Physics and Astronomy, Seoul National University, Seoul, Korea; 47 Planetary Exploration Research Center, Chiba Institute of Technology, Chiba, Japan; 48 National Astronomical Observatory of Japan, Tokyo, Japan; 49 Faculty of Science, Niigata University, Niigata, Japan; 50 Department of Physics, Rikkyo University, Tokyo, Japan; 51 Instituto de Astrofísica de Canarias, University of La Laguna, Tenerife, Spain; 52 Department of Mechanical Engineering, Kanagawa Institute of Technology, Atsugi, Japan; 53 Marine Works Japan Ltd, Yokosuka, Japan; 54 Department of Geology, Rowan University, Glassboro, NJ USA; 55 Department of Earth and Planetary Sciences, Hokkaido University, Sapporo, Japan; 56 Creative Research Institution Sousei, Hokkaido University, Sapporo, Japan; 57 Department of Earth and Planetary Sciences, Kyushu University, Fukuoka, Japan; 58 UTokyo Organization for Planetary and Space Science, The University of Tokyo, Tokyo, Japan; 59 Department of Earth and Environmental Sciences, Nagoya University, Nagoya, Japan

**Keywords:** Meteoritics, Planetary science

## Abstract

Without a protective atmosphere, space-exposed surfaces of airless Solar System bodies gradually experience an alteration in composition, structure and optical properties through a collective process called space weathering. The return of samples from near-Earth asteroid (162173) Ryugu by Hayabusa2 provides the first opportunity for laboratory study of space-weathering signatures on the most abundant type of inner solar system body: a C-type asteroid, composed of materials largely unchanged since the formation of the Solar System. Weathered Ryugu grains show areas of surface amorphization and partial melting of phyllosilicates, in which reduction from Fe^3+^ to Fe^2+^ and dehydration developed. Space weathering probably contributed to dehydration by dehydroxylation of Ryugu surface phyllosilicates that had already lost interlayer water molecules and to weakening of the 2.7 µm hydroxyl (–OH) band in reflectance spectra. For C-type asteroids in general, this indicates that a weak 2.7 µm band can signify space-weathering-induced surface dehydration, rather than bulk volatile loss.

## Abstract (web summary)

A close-up look at the action of space weathering on carbonaceous asteroids, provided by Ryugu’s returned samples, highlights its role on the dehydration of the first micrometre-thick layer of the surface, possibly hiding a water-rich interior. The depth of the 2.7 µm hydration band may be an indication of the level of space weathering withstood by a C-type asteroid.

## Main

Solar wind irradiation and high-velocity micrometeoroid bombardment dominate space weathering^1,2^ for all airless bodies. However, the effects of these processes vary substantially, depending on the specific class of body. The solar wind is a plasma composed mainly of low-energy protons and electrons streaming from our Sun^1–3^, which induces radiation damage, including amorphization of silicates and formation of nanophase metallic iron particles (npFe^0^). In contrast, micrometeoroids are interplanetary dust particles that impact airless surfaces at hypervelocities^4^, resulting in cratering, melting and vapour deposits, and sometimes also amorphous silicates and npFe^0^. Space-weathering products of two anhydrous bodies, the Moon and the S-type asteroid Itokawa, have been investigated extensively^5–14^. These studies revealed that nanometre-sized metallic Fe particles (npFe^0^), formed via space weathering, resulted in weakened absorption features in visible to near-infrared reflectance. In contrast, it has been unclear what role npFe^0^ plays in the reflectance properties of dark (C- and D-type) asteroids^1,2^.

Space-weathering modification of reflectance spectra features from airless bodies makes identifying a direct link between asteroids and specific meteorite classes based on composition and mineralogy difficult. The Hayabusa mission of the Japan Aerospace Exploration Agency (JAXA) revealed the connection between visible to near-infrared reflectance spectra from S-type asteroids and ordinary chondrite meteorites^5^, with the difference largely attributable to the role of nanophase particles. However, laboratory experiments that mimic solar wind irradiation and micrometeoroid impact on C-type asteroids show a lack of detectable production of npFe^0^, with some spectra reddening (a positive change in spectral slope) and others bluing (just the opposite)^15–20^. Thus, the observed change of spectral slope and absorption band in reflectance spectra of C-type asteroids compared with carbonaceous chondrites meteorites is difficult to interpret^15–20^. JAXA’s Hayabusa2 spacecraft observed spectral variation on asteroid Ryugu^21–24^ thought to be related to space weathering. Our studies of Ryugu samples offer the first opportunity to directly link the spectral variation to the space-weathering-induced physical and chemical alteration of regolith on C-type asteroids.

## Results

### Surface modifications found on Ryugu grains

The mineralogy of most Ryugu grains investigated by (scanning) transmission electron microscopy is similar to that of CI chondrites^25^, which are the most chemically primitive materials in the Solar System^26^, consistent with other recent studies^27–31^. Therefore, to understand the space weathering of Ryugu grains is to understand the weathering of the most chemically primitive Solar System material.

**Fig. 1:**
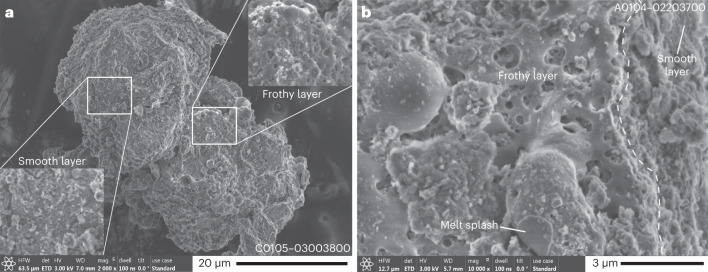
Secondary electron images of Ryugu grains showing surface modifications related to space weathering. **a**, The grain C0105–03004800 was collected at the second touchdown site. It is composed of two parts showing different types of space weathering: a frothy layer and a smooth layer. Enlarged images of the two boxed areas on this grain are shown in the insets at the upper right (frothy layer) and the lower left (smooth layer) corners of **a**. **b**, The grain A0104–02203700 was collected at the first touchdown site. The frothy layer partially covers the smooth layer on the left-hand side of the image. The boundary between two types of layers is indicated by a dashed curve. The frothy layer has many burst vesicles. A melt splash, located at the lower centre of the image, is attached to the surface of the frothy layer. Source data

**Extended Data Fig. 1:**
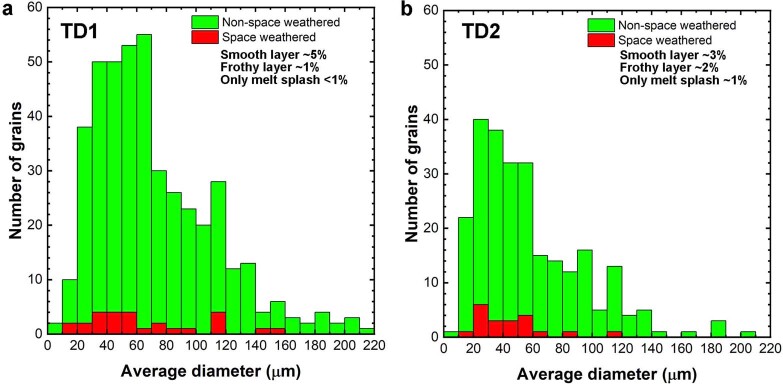
Histograms of average diameters of fine-grained Ryugu samples investigated in this study. Grains recovered from both **(a)** 1st and **(b)** 2nd touchdown (landing) sites (TD1 and TD2) have a skewed size distribution. No clear differences are observed between size distributions of non-space weathered (blue bins) and space weathered (red bins) grains in both touchdown sites. The proportions of grains with smooth layers, frothy layers, and melt splashes on non-space weathered surfaces are expressed as percentages in both **(a)** and **(b)**. Bin ranges are 10 µm. Source data

**Extended Data Fig. 2:**
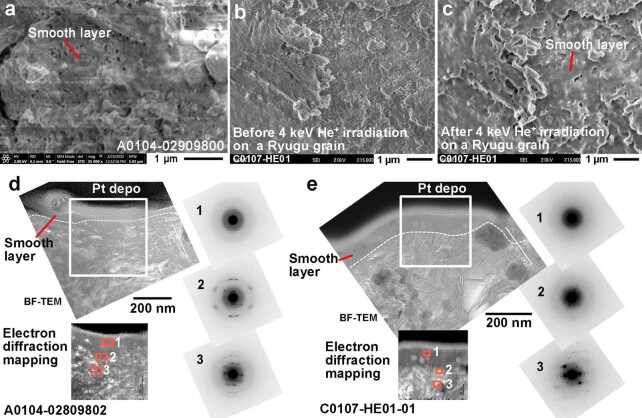
Textural comparison between smooth surfaces and a He+ ion irradiated sample. Secondary electron (SE) images of **(a)** a smooth layer on the Ryugu grain A0104–02909600. (**b, c**) A surface of the non-space weathered Ryugu grain C0107–HE01 **(b)** before and **(c)** after a 4 keV He+ irradiation experiment. (**d, e**) Bright-field (BF) TEM images, diffraction maps, and electron diffraction patterns of **(d)** natural and **(e)** artificial smooth layers on Ryugu grains, which are described in **(a)** and **(b)**, respectively. In both cases, the smooth layers in the cross-section samples A0104–02809802 and C0107–HE01 are amorphized as shown in the insets of electron diffraction patterns #1 in **(d)** and **(e)**. Source data

**Extended Data Fig. 3:**
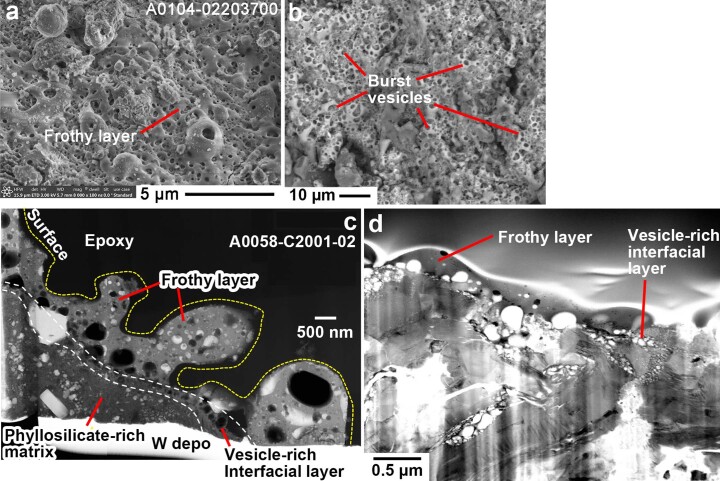
Textural Comparison between frothy layers and laser irradiated samples. **(a)** SE image of a frothy layer on the Ryugu grain A0104–0223700. **(b)** Backscattered electron image of the surface of the Murchison CM chondrite after laser irradiation experiment. In both **(a)** and **(b)**, there are many burst vesicles on grain surfaces. **(c)** HAADF-STEM image of a frothy layer in a cross-section sample A0058–C2001–02 on the large Ryugu grain A0058–C2001. **(d)** BF TEM image of the Murchison meteorite cross-section sample after laser irradiation experiment. One of the run products of the laser irradiation experiments performed by Thompson et al. (2019) was used for comparison. Source data

More than 500 grains (average diameter ~71 µm) collected at the first touchdown (landing) site (TD1) and >300 grains (average diameter ~57 µm) collected at the second touchdown site (TD2) were investigated for surface modifications potentially related to space weathering. Recognizable surface modifications of the phyllosilicate-rich matrix were found in ~6% of the observed grains from TD1 and ~7% from TD2 (Extended Data Fig. 1). The surface modifications of grains differ considerably from those from the Moon and Itokawa because the most abundant phases in Ryugu grains are hydrated sheet silicates (phyllosilicates), not anhydrous silicates (for example, olivine). Several distinct surface modifications are observed, including smooth layers, frothy layers, melt splashes and their combinations (Fig. 1 and Extended Figs. 2 and 3). We also examined three millimetre-sized grains (A0067, A0094 and A0058) that have surface modifications related to space weathering.

### Smooth layers on Ryugu grains

**Fig. 2:**
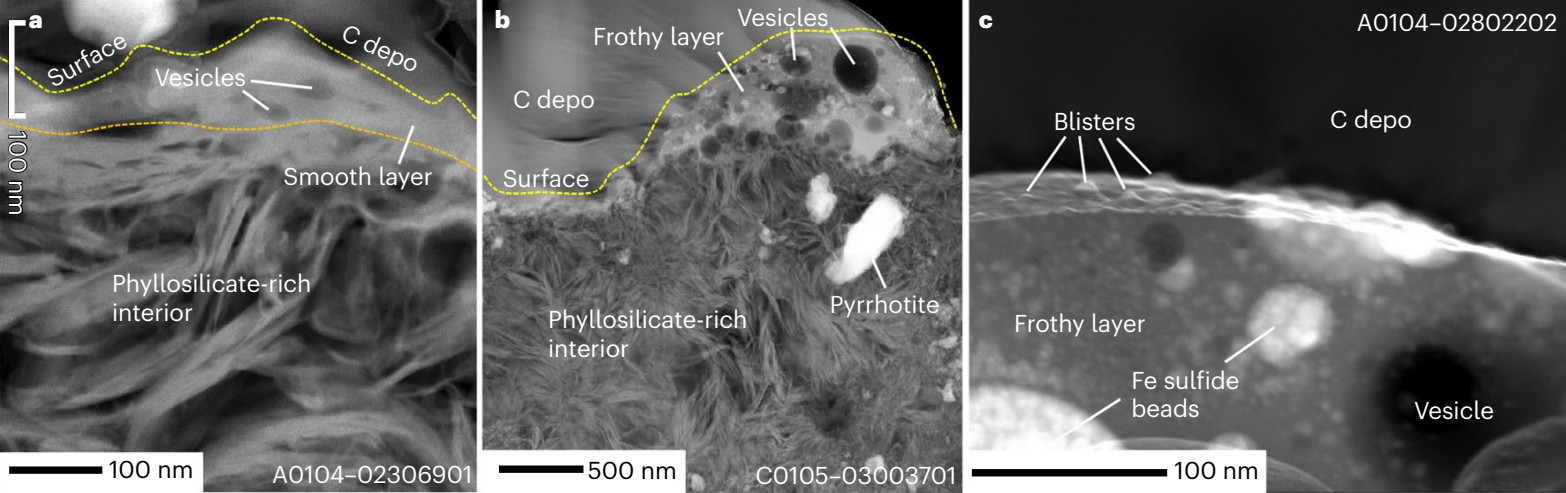
Cross-sections of three Ryugu grains showing typical surface modifications on the phyllosilicate-rich matrix. **a**, The cross-section A0104–02306901 was prepared from the grain A0104–02306900 collected at the first touchdown site. It has a smooth layer that forms a ~100-nm-thick continuous layer covering the surface of the grain. The phyllosilicate-rich matrix is present below the smooth layer. A yellow dashed curve indicates the cross-section of the sample surface. The boundary between the smooth layer and the phyllosilicate-rich interior is indicated by an orange dashed curve. **b**, The cross-section C0105–03003701 was prepared from the grain C0105–03003700 collected at the second touchdown site. A frothy layer containing abundant vesicles (darker circles) and <50-nm-size brighter spots (Fe-Ni sulfide beads) covers the surface of this grain. The thickness of the frothy layer varies considerably locally from <100 nm to >500 nm. A yellow dashed curve indicates the cross-section of the sample surface. C-depo denotes carbon depositions to protect the surface of the samples during FIB processing. **c**, An enlarged image of a frothy layer in a cross-section A0104–02802202. The frothy layer contains many tiny (<20 nm across) blisters (vesicles just below the surface) on its surface. These are high-anglular dark-field scanning transmission electron microscope images, in which materials with higher average atomic numbers are brighter than those with lower average atomic numbers. Source data

**Fig. 3:**
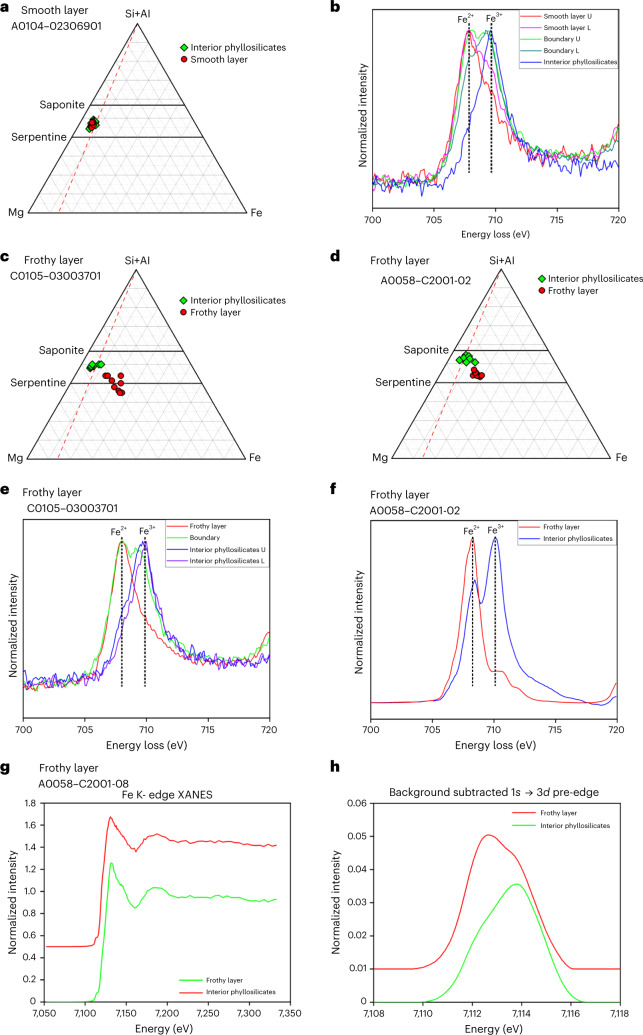
Elemental compositions and redox states of Fe in a smooth layer, frothy layers and the interior phyllosilicates. **a**, The ternary [Si+Al]-Mg-Fe atomic-ratio diagram shows that elemental compositions of a smooth layer are indistinguishable from those of the interior phyllosilicates in the cross-section sample A0104–02306901. **b**, However, a Fe L_3_-edge peak in EELS spectra shows that Fe^2+^ is enriched in the smooth layer, which means that Fe^3+^ in the smooth layer is reduced to Fe^2+^. The EELS spectra were obtained from the upper (U) and lower (L) parts of the smooth layers, the upper (U) and lower (L) areas around the boundary between the smooth layer and the interior phyllosilicates, and the interior phyllosilicates. **c**, By contrast, the frothy layer in the cross-section sample C105–03003700 is more enriched in Fe relative to [Si+Al] and Mg than the interior phyllosilicates. **d**, The same compositional relationship is shown between the frothy layer in the cross-section sample A0058–C2001 and the interior phyllosilicates. The whole grain sizes of these samples are quite different. C105–03003700 and A0058–C2001 are ~30 µm and ~3 mm across, respectively. **e**–**h**, Fe^3+^ in the frothy layers is also reduced to Fe^2+^. **e**, Fe L_3_-edge peak spectra obtained by EELS. The spectra were obtained from the frothy layer, the boundary area between the frothy layer and the interior phyllosilicates, and the upper and lower areas of the interior phyllosilicates. **f**, Fe L_3_-edge peak spectra obtained by STXM–XANES. **g**,**h**, Fe K-edge spectra (**g**) and background-subtracted pre-edge peak spectra (**h**) obtained by XANES. Int. phyllosilicates, phyllosilicates in the interior of a sample.　Serp and Sap in **a**, **c**, and **d** are the abbreviations for serpentine and saponite, respectively. Source data

**Extended Data Fig. 4:**
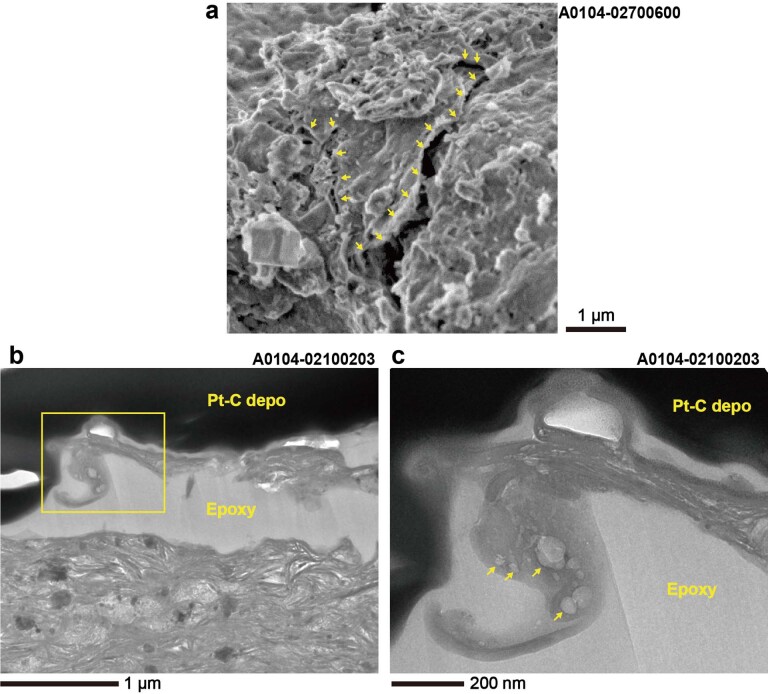
Partially exfoliated smooth layers on phyllosilicate-rich matrix. **(a)** Secondary electron image of a smooth layer on the grain A104–02700600. Partial exfoliation of the smooth surface is indicated by yellow arrows. (b-c) BF-TEM image of an exfoliated smooth layer containing vesicles in the cross-section sample A104–02100203. **(c)**An enlarged image of the area is indicated by a square in **(b)**. The arrows in **(c)** indicate vesicles in the exfoliated smooth layer. The interstice between the exfoliated layer and the phyllosilicate base is filled by epoxy resin, and Pt-C was deposited by FIB-SEM, both from sample preparation. Source data

Approximately 5% of the observed grains from TD1 and ~3% from TD2 have a smooth layer, evident as a thin (<100 nm) continuous smooth sheet covering the surface. Some of these layers contain vesciles of <50 nm diameter that intersect the surface (Fig. 2a and Extended Data Fig. 2a). Partial detachment of the smooth layers is observed in some grains (Extended Data Fig. 4). Electron diffraction reveals that smooth layers are almost completely amorphous (Extended Data Fig. 2d). Atomic ratios among major cations of the smooth layers are indistinguishable from those of the phyllosilicate-rich matrix (Fig. 3a). Most Fe in the smooth layer is Fe^2+^, but most Fe in the underlying phyllosilicates is Fe^3+^ based on the Fe L_3_-edge electron energy-loss spectroscopy (EELS), and Fe L_3_ and Fe K X-ray absorption near-edge spectroscopy (XANES) (Fig. 3b and Supplementary Table 1), indicating that the smooth layer is more reduced than the matrix.

**Extended Data Fig. 5:**
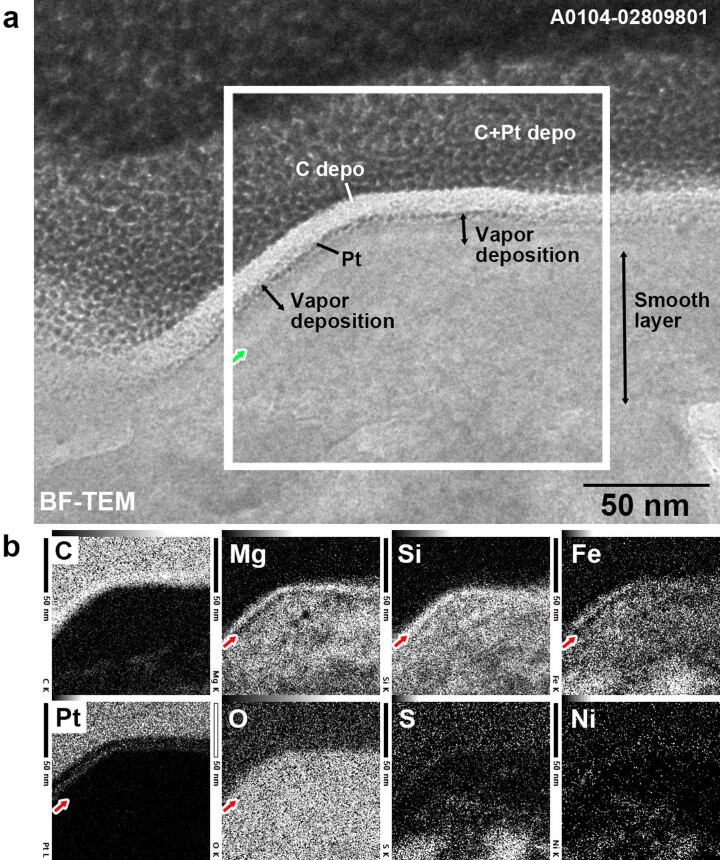
10-nm thick vapor deposit on a smooth layer found in a cross-section sample A104–02809801. A very thin (~10 nm) layer covers the ~60-nm thick smooth layer. EDS maps show that the upper part of the very thin layer is enriched in Mg, Si, Fe, and O, and the lower part is depleted in Mg, Si, and Fe. There are no detectable differences in concentrations in S and Ni. Because similarly thin top surface layers with such element distribution patterns were reported from Itokawa grains6,7, this top surface layer is considered a vapor deposit on the smooth layer. The estimated boundary between the vapor deposition and the smooth layer is indicated by green or red arrows. This is the only vapor deposit found on the surface of smooth layers investigated. Source data

An unweathered Ryugu grain that was irradiated with 4 keV He^+^ at a fluence of 1.3 × 10^18 ^ions cm^−^^2^ to simulate space weathering shows a surface morphology and an internal structure that are very similar to those of the smooth layers (Figs. 1a and 2a, and Extended Data Fig. 2) of the bonafide space-weathered grains. On lunar and Itokawa grains, smooth surfaces were formed by micrometeoroid impacts and subsequent redeposition. On Ryugu grains, in contrast, we found a ~10-nm-thick vapour deposit on top of a smooth layer of only one grain (Extended Data Fig. 5), suggesting that ‘vapour deposition’ does not play an important role in forming smooth layers. Instead, the laboratory-irradiated Ryugu grain indicates that solar wind irradiation probably played an important role in modifying the surface of the phyllosilicate-rich matrix, and the smooth layers represent space weathering induced by solar wind irradiation.

### Frothy layers and melt splashes on Ryugu grains

**Extended Data Fig. 6:**
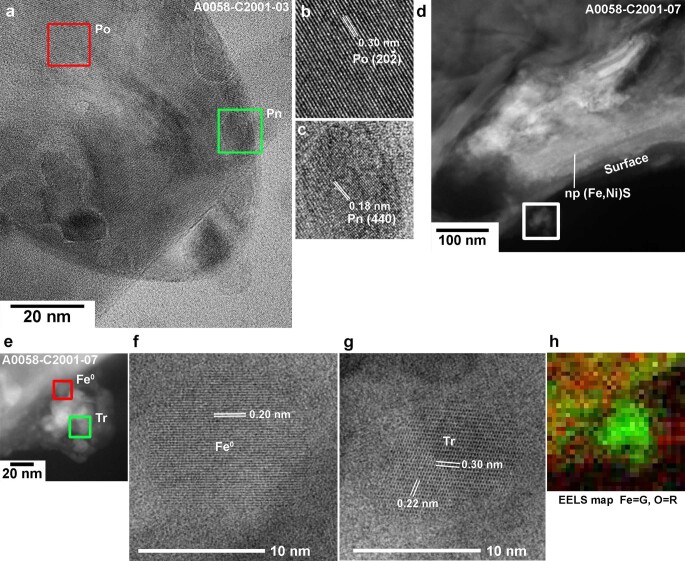
High-resolution images of sulfide and metal in a frothy layer on the large grain A0058–C2001. **(a)** A BF-TEM image of a spherical Fe-Ni sulfide bead in the frothy layer in the cross-section sample A0058-C2001–03. (**b, c**) Enlarged BF-TEM images of the red and green boxed areas are shown in **(b)** and **(c)**, respectively. The lattice fringes shown in **(b)** are 0.30 nm, which suggests $\left( {2\;0\;\bar 2} \right)$ of pyrrhotite (Po). The fringes shown in **(c)** are 0.18 nm, suggestive of (4 4 0) of pentlandite (Pn). **(d)** HAADF-STEM image of the cross-section sample A0058–C2001–07 shows a chain of nanophase (np) (Fe, Ni)S in the thin (~100 nm) smooth layer. (e-h) An aggregate composed of nanophases (<100 nm) on the frothy layer of the cross-section sample A0058–C2001–7. HAADF-STEM image of the aggregate is shown in **(e)**. Enlarged BF-TEM images of the red and green boxed areas in **(e)** are shown in **(f)** and **(g)**, respectively. The lattice fringes shown in **(b)** are 0.30 nm, which suggests $\left( {2\;0\;\bar 2} \right)$ of pyrrhotite (Po). The fringes shown in **(f)** is 0.20 nm, suggestive of (1 1 0) of kamacite (Fe°) and those in **(g)** are 0.22 and 0.30 nm, suggestive of $\left( {1\;\bar 2\;4} \right)$, and (1 1 0) of troilite (Tr). **(h)** EELS map of the same area shown in **(e)** shows that the aggregate lacks oxygen and contains iron. Source data

Frothy layers are found on ~1% of the observed grains from TD1 and ~2% of grains from TD2, which are composed of silicate glass containing abundant embedded vesicles ~0.1- to ~1 µm wide and numerous submicroscopic (<200 nm) rounded Fe-Ni sulfide beads (Fig. 2b and Extended Data Fig. 3). The internal structure suggests that silicate and Fe-Ni sulfides were melted and immiscibly separated into silicate and sulfide melts and that vesiculation occurred during melting. The frothy layers have higher Fe and lower Si+Al and Mg than the interior phyllosilicate-rich matrix (Fig. 3c,d), irrespective of the size of the grain on which they reside. The frothy layer is also more reduced in Fe than the underlying phyllosilicates (Fig. 3e–h). The Fe-Ni sulfide beads, composed of pyrrhotite and pentlandite with diameters from ~200 to <10 nm, are ubiquitous within frothy layers (Extended Data Fig. 6a–d). While most microphase sulfides are probably immiscibly separated as droplets during melting, the ~10-nm-sized nanophase sulfide shown in Extended Data Fig. 6d may be a vapour deposit, consistent with previous laser irradiation experiments^15,16^. We identified no npFe^0^ within the frothy layers investigated. However, on the surface of a frothy layer, we found an aggregate composed of npFe^0^ and troilite (stoichiometric FeS) (Extended Data Fig. 6e–h). Porous apatite, dolomite and magnetite occur in some frothy layers and are believed to be relict minerals that survived melting. In addition, a frothy layer is observed with abundant blisters (vesicles just below the surface) (Fig. 2c). Melt splashes (<10 µm across) are found on <1% of the observed grains from TD1 and ~1% of grains from TD2, and are attached to Ryugu grains with and without detectable surface modifications (Fig. 1b).

### Exceptionally rare npFe^0^ on Ryugu

The exceptionally low abundance of npFe^0^ in Ryugu grains is in stark contrast to lunar and Itokawa surface samples, which contain abundant npFe^0^ in both radiation-damaged layers on ferromagnesian silicates^7–9,11,13,14^ and in vapour deposit layers produced by micrometeoroid impacts^32,33^. While nano- to microphase Fe-bearing sulfides are ubiquitous in all the frothy layers on the Ryugu grains investigated, no interior npFe^0^ was found. The reduction effect of space weathering might be insufficient to form npFe^0^ from abundant Fe^3+^ contained in phyllosilicates in Ryugu grains (Supplementary Table 1). In addition, the –OH in phyllosilicates may hinder the reduction of Fe. These results are similar to laboratory experiments where in situ X-ray photoelectron spectroscopic analyses of H^+^ and He^+^ irradiated Murchison CM2 chondrite showed that partial reduction of surface Fe to lower oxidation states occurred with simulated solar wind exposure^19^. Nano- to microphase Fe sulfide is also common in both H^+^ and He^+^ irradiated Murchison CM2 chondrite and laser-irradiated Murchison. These results are consistent with our observations, although Murchison is a CM chondrite with significantly different mineralogy and Fe content than Ryugu. We suggest that it is unlikely that npFe^0^ contributes significantly to the observed spectral variability on Ryugu, but submicroscopic Fe-Ni sulfides may contribute to this variability.

### More abundant impact melts on Ryugu than on Itokawa

Among Itokawa grains, only 2 out of 590 grains (0.3%) show melted structures^34^ that resemble the frothy layers found on ~1% to 2% of Ryugu grains. The calculated dry and wet solidus temperatures of Ryugu material^28^ are approximately the same (862 and 867 °C) under ~10^5 ^Pa because of the low H_2_O solubility in the melt at low ambient pressures^35,36^, which indicates that high porosity may explain the higher abundance of impact melts among Ryugu grains relative to Itokawa grains. Ryugu grains have high average microporosity (~28%, measured by synchrotron radiation nanotomography) and may have experienced higher post-shock temperature than low-porosity (1.5–1.9%)^37^ Itokawa grains since porosity collapse by shock compression causes a large temperature increase^38^. Both the surface morphology and internal structure of laser irradiation products^15,17^ that simulate shock heating by micrometeoroid impacts are similar to those of the frothy layers (Figs. 1 and 2b, and Extended Data Fig. 3). Thus, one of the major formation mechanisms of the frothy layers might be frictional heating among loose regolith grains by meteoroid impact. In addition, in situ formation of melt by micrometeoroid impact onto the grains and deposition of melt formed by a neighbouring impact event would also contribute to the formation of the frothy layers. The observed small melt splashes might be ejecta formed during micrometeoroid cratering.

## Discussion

**Extended Data Fig. 7:**
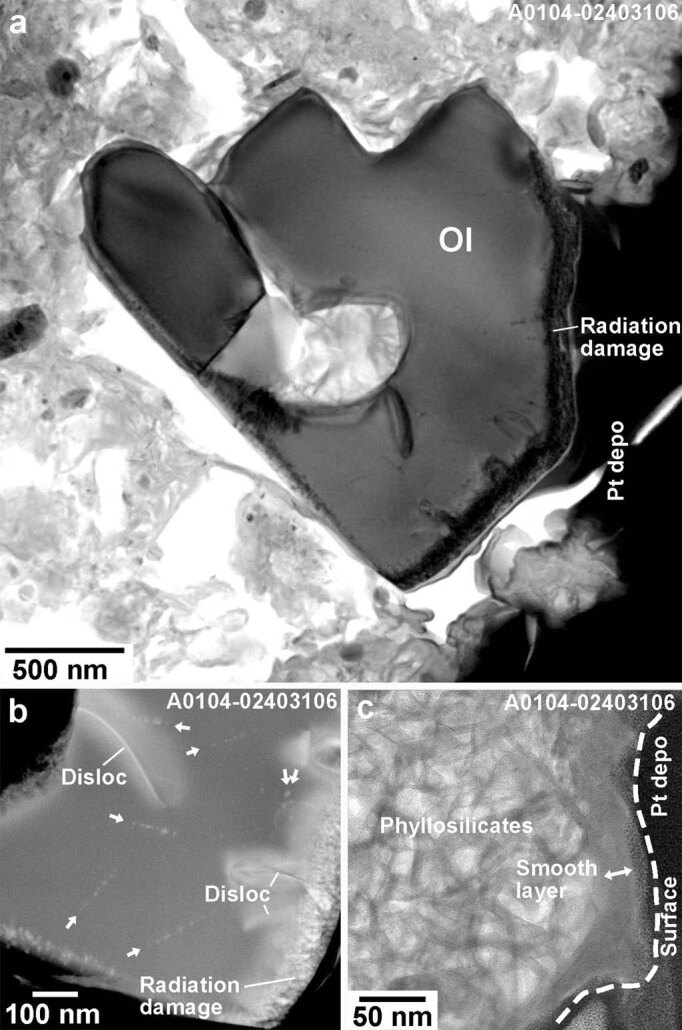
An olivine crystal and the surrounding phyllosilicate-rich matrix in the rare olivine-bearing grain A0104–02403200. **(a)** BF-TEM image of radiation-damaged olivine and highly porous hydrated matrix in the cross-section sample A0104–02403206, which was prepared from A0104–02403200. **(b)** Low-angle annular dark-field (LAADF)-STEM image shows that the olivine contains solar flare tracks indicated by arrows as well as dislocations (Disloc) and a radiation damage layer. **(c)** BF-TEM image shows that a thin (~20 nm) continuous smooth layer covers the phyllosilicate-rich matrix of the cross-section sample A0104–02303204, which was prepared from A0104–02403200. Source data

**Extended Data Fig. 8:**
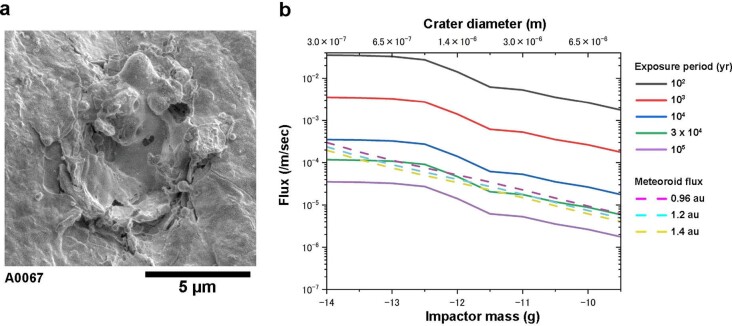
Exposure age of the surface of a millimeter-sized Ryugu grain A0067 estimated from the impact crater population. Forty craters with average diameters ranging from 300 nm up to 8.5 µm were examined to estimate the exposure age (see Methods). **(a)** Secondary electron image of one of the investigated craters. **(b)** The cumulative impactor flux versus the mass of the impactor, assuming exposure time from 10^2^ years to 10^5^ years (solid lines). Broken lines indicate interplanetary meteoroid flux models at Ryugu’s perihelion (0.96 au [astronomical unit]), average orbit (1.2 au), and aphelion (1.4 au). This diagram shows that the exposure age of the smooth layer-covered surface of A0067 is estimated to be 3 × 10^4^ years, calculated by its crater population and assuming craters formed by interplanetary meteoroid impacts, even if the average semimajor axis of the orbit of Ryugu had changed from 0.96 to 1.4 au. Source data

We estimated the timescale of formation of the smooth layer on Ryugu grain surfaces. The fluence of the ion irradiation experiment (Extended Data Fig. 2c) is equivalent to ~3 × 10^3^ years at 1.2 au (the semimajor axis of Ryugu’s orbit) by considering the solar wind flux density at 1 au, 3–5 × 10^8^ ions cm^−^^2 ^s^−1^ (ref. ^39^), and the average He/H ratio in the solar wind, 0.045 (ref. ^40^). We found an olivine crystal exhibiting a radiation-damaged rim and containing solar flare tracks with a number density of ~2 × 10^8^ cm^−^^2^ (Extended Data Fig. 7), which corresponds to an ~6 × 10^3 ^year dwell time for the grain within ~1 mm from the surface, based on lunar sample studies^41^. A thin (~20 nm) smooth layer on the phyllosilicate matrix was found near the olivine grain in the same sample. These independent results suggest that it may take >3 × 10^3^ years to form a detectable smooth layer on phyllosilicates. The exposure age of the smooth layer-covered surface of Ryugu grain A0067 is estimated to be 3 × 10^4^ years, calculated by its crater population assuming they formed by interplanetary meteoroid impacts^4^ (Extended Data Fig. 8). In comparison, studies of craters on Itokawa grains^42,43^ showed that most submicrometre-scale craters were probably formed by secondary impacts of ejecta excavated from larger craters. If such impacts occurred on Ryugu, then the formation of the smooth layer would require less time. In either scenario, the upper limit on time required to develop the smooth layer is 3 × 10^4^ years, which is consistent with the above estimate.

A frothy layer with abundant blisters (Fig. 2c) suggests that, after irradiation by the solar wind, blisters formed during subsequent heating, probably related to micrometeoroid impact, which induced the release of trapped solar wind gas species. This is consistent with steady and continual solar wind irradiation through time, while micrometeoroid bombardment occurs sporadically.

Some grains have partially exfoliated smooth layers (Extended Data Fig. 4), which suggests that smooth layers can detach. Detachment of smooth layers may explain the low abundance (~7%) of investigated grains with space-weathered features. In addition, the fragility of Ryugu grains may be another important factor that reduces the abundance of grains with observable space weathering. Among 6 large (millimetre-sized) grains collected at TD1, ~66% (4 of 6) show evidence of space weathering based on the field emission scanning electron microscope (FE–SEM) observation at Kyoto and Tohoku Universities, which is much higher than 6–7% for <100-µm-sized grains (Extended Data Fig. 1). The difference can be interpreted as indicating that most fine-grained samples are fragments of larger grains. Exfoliation and destruction could occur by thermal fatigue and meteoroid impacts on Ryugu. In addition, they could also occur during sampling and transportation to Earth, or even during handling processes.

**Fig. 4:**
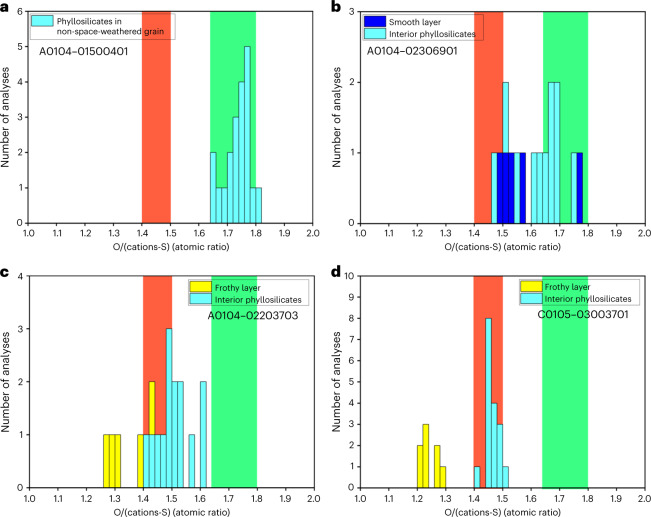
Histograms of atomic ratios of oxygen to the cations bonded to oxygen in phyllosilicates, a smooth layer and frothy layers. A mixture of saponite without interlayer H_2_O molecules and serpentine has a range of ratios represented by green bands. If a mixture of saponite and serpentine is decomposed into an anhydrous compound, it has a range of ratios represented by red bands. In order to calculate the atomic ratios of oxygens to the cations bonded to oxygen in phyllosilicates, we subtracted the cations bonded to sulfur (S), which were calculated based on the assumption that the ratio of the S-bonded Fe and Ni ions to S is unity for simplicity. **a**, Phyllosilicates in a non-space-weathered grain contain almost no interlayer H_2_O but preserve structural –OH groups. **b**, A smooth layer lost a considerable amount of structural –OH groups and phyllosilicates just below the smooth layers partially lost structural –OH groups. **c**, Phyllosilicates just below the frothy layer have lost the structural –OH groups considerably. **d**, Phyllosilicates just below the frothy layer have lost almost all the structural –OH groups.Because the frothy layers have even lower ratios than the red bands, they are also anhydrous. Their very low ratios may be related to their high abundance of embedded Fe-Ni sulfide. The ratio at the right end of the green belts is 1.8, which is calculated from the generalized chemical formula of serpentine Y_6_Z_4_O_10_(OH)_8_. O/(Y + Z) = 18/10 = 1.8. The ratio at the left end of the green belts is 1.64, which is calculated from the generalized chemical formula of saponite with no interlayer H_2_O molecules X_0.6_Y_6_Z_8_O_20_(OH)_4_. O/(X + Y + Z) = 24/14.6 = 1.64. The ratio at the right end of the red belts is 1.5, which is calculated from the generalized chemical formula of the dehydrated decomposition product of saponite X_0.6_Y_6_Z_8_O_22_. O/(X + Y + Z) = 22/14.6 = 1.5. The ratio at the left end of the red belts is 1.4, which is calculated from the generalized chemical formula of the dehydrated decomposition product of serpentine Y_6_Z_4_O_14_. O/(Y + Z) = 14/10 = 1.4. Source data

To quantify the amount of –OH in the Ryugu grain surfaces, we used energy dispersive X-ray spectroscope (EDS) measurements of focused ion beam (FIB) cross-sections of weathered and pristine grains (Fig. 4) to determine oxygen to cation ratios, correcting for S-bonded Fe and Ni. The ratio of oxygen to cations bonded with oxygen shows that, in pristine grains, interlayer H_2_O molecules in saponite are largely absent from a mixture of saponite and serpentine, but structural –OH groups in the phyllosilicates are retained (Fig. 4a). This estimation of –OH abundance is consistent with the thermogravimetric analysis of Ryugu grains^28^.

Space weathering over the length of Ryugu’s residence time at near-Earth orbits after its orbital shift from the main belt, which is thought to be several megayears based on noble gas data^44^_,_ may also play an important role in the removal of interlayer H_2_O from saponite. During the prolonged exposure to interplanetary space, even structural –OH groups might be removed from a mixture of serpentine and saponite that has lost its interlayer H_2_O. This may occur by decomposition of saponite and serpentine by dehydroxylation, that is, a decomposition to anhydrous compounds and liberated H_2_O molecules. However, we note that the solar wind would probably penetrate grains very heterogeneously owing to the high microporosity of the phyllosilicate-rich matrix of Ryugu grains.

**Fig. 5:**
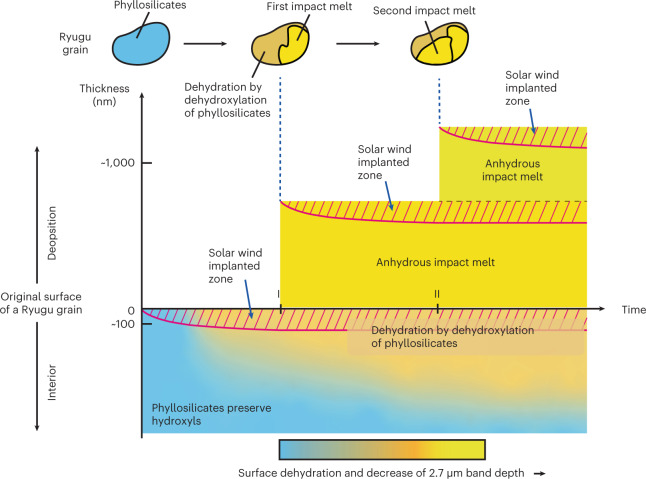
A conceptual illustration showing the development of two types of space weathering with dehydration by dehydroxylation observed on a Ryugu grain. Once a surface of a Ryugu grain is exposed to interplanetary space, the effects of solar wind irradiation start to accumulate at and near the surface, which is shown as hatched areas labelled as the ‘Solar wind implanted zone’ in the figure. As time passes, the gradual accumulation of solar wind radiation damage and phyllosilicate dehydroxylation form the smooth layer on its surface with a thickness that seldom exceeds ~100 nm. In contrast, the formation of impact melts (frothy layer, cratering and melt splash) is an intermittent process. In this conceptual illustration, partial coverage by impact melt occurred twice at times I and II. The change of colour from light blue via orange to yellow represents the progress of dehydration. As shown in Fig. 4, the impact melts are almost anhydrous. Therefore, the surface of the model grain is covered by both nearly anhydrous impact melts and dehydroxylated amorphized phyllosilicates. As a result, the surface of the asteroid Ryugu becomes covered by anhydrous material over time. After a long period of space exposure, dehydration by dehydroxylation of the phyllosilicates proceeds below both the smooth layers and the frothy ones. Note that the natural overturn, or gardening, of regolith grains on the asteroid parent body interrupt the schematic history of space weathering so that the space-weathering processes on any one grain do not necessarily progress as shown in Fig. 5.

A substantial amount of structural –OH has been lost in smooth layers (Fig. 4b). Almost all the structural –OH has been lost in frothy layers and also in the phyllosilicates just below frothy layers (Fig. 4c,d). These data suggest that more structural –OH in phyllosilicates is removed through dehydroxylation as space weathering proceeds (Fig. 5). A portion of structural –OH in phyllosilicates just below the smooth layer also appears to be absent (Fig. 4b), and sporadic amorphization of phyllosilicates is observed. Solar wind particles could penetrate to such depths, in regions of highest porosity of the phyllosilicate-rich matrix (Fig. 2a), and potentially promote dehydroxylation reactions. In addition, phyllosilicates just below the frothy layer lost most of their structural –OH (Fig. 4c, d). Frictional heating induced by meteoroid impacts and the formation of new surfaces by thermal stress^45^ could promote dehydration by dehydroxylation.

A conceptual illustration (Fig. 5) shows the development of solar wind implantation, dehydration by dehydroxylation of phyllosilicates and progressive coverage of anhydrous silicate-rich melt on a Ryugu grain. Once a surface of a Ryugu grain is exposed to interplanetary space, the effects of solar wind irradiation start to accumulate at and near the surface. As time passes, the gradual accumulation of solar wind radiation damage and phyllosilicate dehydroxylation form the smooth layer on its surface, which means that partial dehydration occurs in the smooth layer as shown in Fig. 4b. Because the effects of solar wind are constrained by the limited kinetic energy of solar wind particles^3^, the thickness of the smooth layer seldom exceeds ~100 nm. Sometimes, a very thin (~10 nm) vapour deposition may be formed on its surface as shown in Extended Data Fig. 5, although it is not illustrated in Fig. 5.

In contrast, formation of impact melts (frothy layers and melt splashes) is an intermittent process. The impact melt can be formed in several ways: in situ formation of melt by micrometeoroid impact melt onto the grain, deposition of melt formed by a neighbour impact event and in situ melting by frictional heating among porous regolith. In this conceptual illustration, partial coverage by impact melt occurred twice, at times I and II. After the coverage by a frothy layer, the effects of solar wind irradiation start to accumulate in the frothy layer. If another subsequent heating event or impact occurs, resulting in the deposition of another melt deposit, the implanted solar wind gases in the frothy layer may form blisters (Fig. 2c). The frothy layers (impact melts) are almost anhydrous because they were formed by high-temperature processing, which is indicated in Fig. 4c,d.

In Fig. 5, the change of colour from light blue via orange to yellow represents the progress of dehydration. The surface material of asteroid Ryugu becomes covered by nearly anhydrous material over time. After a long period of space exposure, dehydration by dehydroxylation of the phyllosilicates proceeds below both the smooth layers and the frothy ones, as shown in Fig. 4. We measured the chemical compositions of the frothy and smooth layers and the underlying phyllosilicate-rich matrix from the surface of grains to ~1.5 µm below the surface, demonstrating that the effects of dehydration by dehydroxylation extend to at least that depth in space-weathered Ryugu grains. Note that the natural overturn, or gardening, of regolith grains on the asteroid parent body interrupt the schematic history of space weathering so that the space-weathering processes on any one grain do not necessarily progress as shown in Fig. 5.

Strong absorption in the 3 µm region of the reflectance spectra^1,2^ is attributed to phyllosilicates and other –OH-rich minerals as well as H_2_O ice. Among these materials, the 2.7 µm band is ascribed to –OH^1,2^. Dehydroxylation from phyllosilicates may weaken the band. Spacecraft-based measurements of the reflectance spectra obtained at the artificial crater at TD2 on Ryugu normalized to a surface standard spectrum show a 2.7 µm band depth inside the crater that is ~5% stronger than that of the standard spectrum and those from outside the crater^46^. These observations suggest that more –OH is preserved in the subsurface material that was exposed by the formation of the artificial crater. Additional factors such as grain size, porosity and viewing geometry can also affect reflectance spectra^47–49^. Considering all these factors together, we hypothesize that the 2.7 µm band features are potential indicators of the degree of space weathering for C-type asteroids.

In addition to the band depth, band shift could be an important indicator of the degree of space weathering, but a definitive interpretation of shift is so far elusive, with different experiments indicating different shift directions. Measurements of the Ryugu artificial crater showed a small shift, but it is difficult to link this to measurements at the scale of individual particles. Several experiments have tried to simulate space weathering induced by solar wind irradiation or micrometeoroid impact to assess impact on spectral features^15–20,48–50^. However, none yet fully satisfy realistic space-weathering conditions and include coordinated near-infrared spectroscopy, scanning electron microscopy and transmission electron microscopy on the irradiated samples. Further studies are greatly needed to accurately reproduce the features of space weathering of Ryugu samples.

In the case of Ryugu, strong thermal alteration on the parent body^23,46^ can be excluded as the cause for surface dehydration because phyllosilicates in nearly all Ryugu samples preserve their structural –OH^27,28^. Therefore, either space weathering, solar radiative heating or both could have caused these differences^22^. If the surface material of Ryugu was heated by solar radiation^23^, all the grains and boulders from the surface to 10 to 100 cm deep must have experienced heating and dehydration throughout their interiors^23^. However, no heavily heated grains were found in this and other studies^28–31,44^. Therefore, solar radiation heating is unlikely to have caused the decrease of the 2.7 µm feature. As described previously, space-weathered Ryugu grains have an almost completely dehydrated surface and an interior with no evidence of thermal metamorphism. If the surface material contains a higher number of space-weathered grains than the subsurface material, as expected based on regolith gardening processes, the spectral differences before and after crater formation can be explained by different amounts of space-weathered grains in the surface and subsurface material of Ryugu. Therefore, it is likely that space weathering played an important role in the dehydration of Ryugu’s surface.

We note that ~40% of C-type asteroids do not show the 2.7 µm band features (sometimes generally referred to as the 3 µm band) and several interpretations were proposed for their origins^51–53^. Based on our data from Ryugu grains, we propose that the absence of the 2.7 µm absorption band can be at least partly explained by surface dehydration due to space weathering. Gradual covering by anhydrous amorphous silicate with longer exposure to space weathering was proposed by a radiative transfer study of Bennu^20^. The clear 2.7 µm feature on carbonaceous asteroid Bennu^54^ explored by NASA’s OSIRIS-REx spacecraft may be related to weaker space weathering experienced by Bennu than Ryugu or due to differences in phyllosilicate species and their chemical compositions. Suppression of the ‘water band’ by space weathering of C-type asteroid surfaces has implications for interpreting remote spectra, the first and least expensive tool for identifying water resources for eventual in situ resource utilization in space. Asteroids that appear dry on the surface may be water-rich, potentially requiring revision of our understanding of the abundances of asteroid types and the formation history of the asteroid belt.

## Methods

### Sample transfer and preparation for analyses

To preserve the pristine nature of the returned samples, the samples were prepared and analysed without cleaning, washing or other procedures that could introduce terrestrial contamination. The sample catcher has three separate chambers to store samples collected in the different locations on Ryugu. Chambers A and C contain samples collected from the first and second touchdown sites (TD1 and TD2), respectively. Air-tight sample transfer holders^55^ were used to bring samples from JAXA to Kyoto University. The allocated grains were handled in an N_2_-filled glove box at Kyoto University. Ryugu grains from both chambers were attached to Au plates on pin stubs with small amounts of epoxy glue. About 250 grains and about 40 thin foil sections prepared by FIB were investigated at the 21 hub universities and laboratories.

### FIB–scanning electron microscopy

Observation and sample preparation at Kyoto University are as follows. Surface morphology of about 300 grains was observed by a JEOL JSM-7001F FE–SEM. We observed them 15 pA current and 2 kV acceleration voltage. FIB sections were prepared using a Thermo Fisher Helios FIB–SEM. Selected areas were cut out with a 30 kV Ga^+^ ion beam. Before the extraction, the target areas were Pt-C coated by a 2 kV electron beam. Then, Pt-C was deposited on the target areas by 16 or 30 kV Ga^+^ ion beams. The sections mounted on the TEM grids were thinned to a thickness of 50 to 200 nm on the 12 or 16 kV Ga^+^ ion beams. The damaged layers were removed using a 2 kV Ga^+^ ion beam. About 70 FIB sections were prepared and investigated by the team. In parallel with the above work, we also performed FIB and (scanning) transmission electron microscopy under air-free conditions using an air-tight FIB–SEM sample transfer holder and a double tilt LN_2_ Atmos Defend Holder (Mel-Build Corporation) at Kyushu University. Another air-tight sample holder was used to transfer the samples. An Ar-filled glove box was used for sample handling. A Thermo Fisher Scios FIB–SEM was used for the observation of about 500 grains and for FIB processing of space-weathered grains. The conditions of the FIB psrocessing are similar to those at Kyoto University.

### (Scanning) transmission electron microscopy

At Kyoto University, a JEOL JEM 2100 F (scanning) transmission electron microscope ((S)TEM) operating at 200 kV equipped with a JEOL JED-2300T EDS was used. The ζ-factor method^56^ was used for quantitative analysis. Electron diffraction maps with quasi-parallel illumination were acquired using a Gatan Orius200D camera. At Kyushu University, a monochromatized and Cs aberration-corrected Thermo Fisher Titan Cubed G2 operating at 300 kV, equipped with four-quadrant windowless super-X silicon drift detector EDS and Gatan Quantum 965 image filter (GIF) for EELS was used. The probe current was less than 200 pA for TEM observation and ~60 pA for STEM observation as well as energy dispersive X-ray spectroscopy and EELS. The typical energy resolution of this EELS analysis was 0.4 eV. The energy dispersion was 0.1 eV per channel at the camera for EELS, which was calibrated by using standard samples of fayalite (Fe_2_SiO_4_) and synthetic Co olivine (Co_2_SiO_4_). EELS mapping was conducted using ~6 nm × ~6 nm square pixels and the acquisition time per pixel was 100 ms. The obtained EELS spectra were averaged over several hundred pixels (~10,000 nm^2^) to improve the signal-to-noise ratio. The Fe^3+^/ΣFe ratio was quantified by the Fe L_3_ peak as follows. The background was first subtracted using a power-law fit over an energy range of 20 eV, then the spectrum was decomposed to Fe^2+^ and Fe^3+^ components by multiple linear least-squares fitting based on two standard spectra obtained from fayalite (Fe_2_SiO_4_) and Fe_2_O_3_ in the energy range corresponding to the Fe L_3_ peak (705–715 eV). The Fe^3+^/ΣFe ratio was calculated from the ratio of the integrated spectral intensity of the Fe^3+^ component to the sum of the integrated spectral intensity of both components. The EDS acquisition time per pixel was 10 µs. For quantitative analysis, Cliff–Lorimer correction was used. *K*-factors were determined using many mineral standards. At Tohoku University, a JEOL JEM-2100F (S)TEM operating at 200 kV was used. *K*-factor correction was based on several mineral standards. At the University of Hawai’i at Mānoa, a monochromatized and Cs aberration-corrected Thermo Fisher Titan G2 (S)TEM operating at 300 kV, equipped with EDAX^®^ thin-window EDS and Gatan Tridium EELS was used. A ‘TitanX’ ChemiSTEM and a Thermo Fisher Titan G2 STEM were also used for additional analysis at the Molecular Foundry, Lawrence Berkeley National Laboratory. At Université de Lille, a monochromatized and Cs aberration-corrected Thermo Fisher Titan Themis (S)TEM operating at 300 kV, equipped with four-quadrant windowless super-X SDD and Gatan Quantum 966 ERS GIF for EELS was used. For quantitative analysis, Cliff–Lorimer correction was used. *K*-factor correction was based on several mineral standards. At the University of Arizona, a Cs aberration-corrected Hitachi HF5000 (S)TEM, equipped with an Oxford Instruments X-Max 100 TLE EDS system and a Gatan GIF Quantum ER (model 965) electron energy-loss spectroscope was used. The microscope was operated at 200 kV using a 100 pm probe. The energy dispersion was set to 0.25 eV per channel. Maps were acquired by averaging three frames with a relatively large pixel time of 0.2 s for core loss and 0.001 s for the low loss. To quantify the Fe^3+^/Fe^2+^ ratio, FeO, Fe_2_O_3_ and Fe_3_O_4_ were used as standards. The samples were measured with a dispersion of 0.25 eV per channel. The background was subtracted using a power-law fit over an energy range of 100 eV. Then, a linear fit was applied in the pre-edge region for the removal of any residuals to ensure a null background intensity. After the background removal, the continuum intensity beneath the edge was subtracted, using a double arctan function^57^. The Fe L_3a_ and Fe L_3b_ peak maxima of FeO and Fe_2_O_3_ were shifted to 708.7 eV and 710.25 eV, respectively, by systematically applying an offset of 3.19 eV to match the energies described in the literature^58^. The spectrum images were acquired over an area measuring 76 × 20 pixels and 2.83 × 0.75 µm. The Fe L_2,3_-edge was quantified using the methods described in the literature^58,59^. The obtained calibration curve was applied to each pixel of the spectrum image to determine the Fe^3+^/ΣFe. At the Naval Research Laboratory, the Nion UltraSTEM200-X equipped with a Gatan Enfinium ER electron energy-loss spectroscope and a windowless Bruker SDD EDS was used. Bright-field TEM images were collected on a JEOL2200FS TEM, equipped with a Gatan OneView camera. Quantification of STEM-EDS data was performed with the Cliff–Lorimer method. The EELS measurements have a typical energy resolution of 0.5 eV.

### Scanning transmission X-ray microscopy

The HERMES scanning transmission X-ray microscopy (STXM) beamline at the synchrotron SOLEIL was used. Analytical conditions for X-ray absorption near-edge structure (XANES) analysis using STXM is as follows. Energy calibration was done using the 3*p* Rydberg peak of gaseous CO_2_ at 294.96 eV as well as an internal haematite standard. The microscope operates under a high vacuum at 10^–5^ mbar. Stacks of images are collected at the Fe L_2,3_-edge in the energy range 680–720 eV, with an energy increment of 0.15 eV in the spectral range of the two main peaks associated with Fe^3+^ and Fe^2+^ absorption. The dwell time per pixel was fixed to 1 ms. The beam is focused onto the sample using a Fresnel zone plate of 25 nm. We selected pixel sizes of ~40 nm. The hyperspectral dataset was extracted and processed using the Hyperspy Python-based package^60^. The Fe^3+^/ΣFe ratio was quantified at each pixel^58,59^. The background is first subtracted, then a double arctangent is fitted and subtracted to take into account the iron content variation. Then the spectrum is integrated over the energy range corresponding to the Fe^3+^ absorption (708.8–712 eV) and the retrieved value is divided by the spectrum integrated over the full energy range (705–712 eV). This ratio is converted into the Fe^3+^/ΣFe ratio using calibration curves obtained on silicate standards. Ultimately, the component map was created by a linear least-squares fitting of the hyperspectral dataset, using component end-member spectra as inputs (oxidized silicates, melted silicate and sulfides).

### Synchrotron Fe K X-ray absorption spectroscopy

The I 14 X-ray Nanoprobe Beamline at Diamond Light Source, UK was used to achieve X-ray absorption spectroscopy mapping and X-ray fluorescence maps were obtained, each map measured at varied energies ranging from 7,050 to 7,350 eV with a higher energy resolution range over the XANES features (~7,100–7,150 eV). The XANES maps were processed using Mantis v.2.3.02 (ref. ^61^) and isolated spectra normalized in Athena v.0.8.056 (ref. ^62^).

### Microcrater measurement

Forty impact craters ranging from 0.5 to 8.5 µm in average diameters on a millimetre-sized grain (A0067) were measured using a FIB–SEM at Kyoto University. The surface area of 3.6 × 10^5^ µm^2^ was observed. The cumulative impactor flux *F*(*m*) was calculated as *F*(*m*) = *N*(*m*)/*ST*, where *m* is the mass of the impactor, *N(m)* is the cumulative distribution of the impactor, *S* = 3.6 × 10^5^ µm^2^ is the total investigated area of the Ryugu grain and *T* is the exposure time needed for craters to accumulate in the space environment. The mass of the impactor (*m*) is calculated from the diameter of the craters, assuming that an impactor is a spherical object having a density of 3 g cm^−^^3^ and that the ratio of the crater diameter *D* to the impactor diameter *d* (*D*/*d*) is assumed to be 1.60 based on laboratory impact experiments^63,64^. For comparison, interplanetary meteoroid flux is calculated using the models in the literature^4,64,65^.

### Microporosity measurements using X-ray nanotomography

Microporosity was estimated from the results of scanning imaging X-ray microscopy^66^ performed at SPring-8 BL47XU on Ryugu regolith samples (~15–80 µm; 27 particles). The samples were mounted on Ti needles using a FIB–SEM at Kyoto University, and differential phase images were obtained by scanning X-rays at 8 keV. Then 180° images were obtained every 0.4–1.2°, followed by phase recovery and tomographic reconstruction to obtain three-dimensional images of phase contrast with refractive index decrements (RIDs). The pixel size was ~100 × 100 × 100–200 nm. The grain surfaces were defined by automatic segmentation^67^ and manual modification from the three-dimensional images, and their average RIDs were obtained. The RIDs (*δ*) are approximately proportional to the material density (*ρ*), as expressed by the following equation^68^: *δ* = *aρ*^*b*^, where *a* = 3.7174, *b* = 0.87132. In this manner, the average RIDs were converted to the material density. Microporosity is the ratio of bulk density to particle density subtracted from 1. Since the particle density of the Ryugu has not been measured, the grain density of the Orgueil meteorite (2.42 × 10^3^ kg m^−^^3^)^69^ was used to estimate the microporosity.

### Helium irradiation experiments

The irradiation experiments were performed at ISAS/JAXA. Irradiation experiments of 4 keV He ions onto a Ryugu grain C0107–HE01 (~300 × ~200 µm) were performed in a vacuum. The grains were fixed on a gold substrate with a small amount of epoxy glue. The ion flux was kept at ~1.5 × 10^13^ ions cm^–2^ s and the total dose of the irradiated ions was 1.3 × 10^18^ ions cm^–2^. The surface textures before and after irradiation were observed with a JEOL JSM-7000F FE–SEM at the University of Tokyo without carbon deposition under low acceleration voltage and low current conditions (2 kV and 50 pA). FIB thin foil preparation and (S)TEM observation were performed at Kyoto University.

## Supplementary information

Supplementary Information Supplementary Table 1.

## Data Availability

All data needed to evaluate the conclusions in the paper are present in the paper and the Supplementary Information. All data are also available through the DARTS archive (https://data.darts.isas.jaxa.jp/pub/hayabusa2/paper/sample/.Noguchi_2022/). Source data are provided with this paper.

## Source data

Source Data Fig. 1 Unprocessed images that were used in Fig. 1.
Source Data Fig. 2 Unprocessed images that were used in Fig. 2.
Source Data Fig. 3 Original graphs that were used in Fig. 3, and excel data to make these graphs.
Source Data Fig. 4 Original graphs that were used in Fig. 4, and excel data to make these graphs.
Source Data Extended Data Fig. 1 Original graphs that were used in Extended Data Fig. 1, and excel data to make these graphs.
Source Data Extended Data Fig. 2 Unprocessed images that were used in Extended Data Fig. 2.
Source Data Extended Data Fig. 3 Unprocessed images that were used in Extended Data Fig. 3.
Source Data Extended Data Fig. 4 Unprocessed images that were used in Extended Data Fig. 4.
Source Data Extended Data Fig. 5 Unprocessed images that were used in Extended Data Fig. 5.
Source Data Extended Data Fig. 6 Unprocessed images that were used in Extended Data Fig. 6.
Source Data Extended Data Fig. 7 Unprocessed images that were used in Extended Data Fig. 7.
Source Data Extended Data Fig. 8 An unprocessed image and a graph that were used in Extended Data Fig. 8, and an excel file to make the graph.
