# Supplementary material for: A dehydrated space-weathered skin cloaking the hydrated interior of Ryugu
Source: Nat Astron. 2022 Dec 19;7(2):170–81. doi: 10.1038/s41550-022-01841-6 (PMC9943745; doi:10.1038/s41550-022-01841-6)
Supplement: Supplementary file 1 — Supplementary Table 1. [file 41550_2022_1841_MOESM1_ESM.pdf]

nature astronomy

Article

https://doi.org/10.1038/s41550-022-01841-6

A dehydrated space-weathered skin cloaking the hydrated interior of Ryugu

In the format provided by the
authors and unedited

Supplementary Table 1 $Fe^{3+}/\Sigma Fe$ ratios in the space-weathered surface and the interior phyllosilicates estimated by various methods.

Supplementary Table 1 $Fe^{3+}/\Sigma Fe$ ratios in the space-weathered surface and the interior phyllosilicates estimated by various methods.

Sample No.	Chamber	Targets	Fe3+//ΣFe ratios	Methods
A0104-02306901	A	Interior phyllosilicates	~0.85	Fe $L_{2,3}$ ELNES
A0104-02306901	A	Smooth layer	~0.45	Fe $L_{2,3}$ ELNES
C0105-030003701	C	Interior phyllosilicates	~0.95	Fe $L_{2,3}$ ELNES
C0105-030003701	C	Frothy layer	~0.20	Fe $L_{2,3}$ ELNES
A0058-C2001-02	A	Interior phyllosilicates	~0.60	Fe $L_{2,3}$ XANES
A0058-C2001-02	A	Frothy layer	~0	Fe $L_{2,3}$ XANES
A0058-C2001-03	A	Interior phyllosilicates	~0.70	Fe $L_{2,3}$ ELNES
A0058-C2001-03	A	Frothy layer	~0.30	Fe $L_{2,3}$ ELNES
A0058-C2001-08	A	Interior phyllosilicates	~0.40	Fe K XANES
A0058-C2001-08	A	Frothy layer	~0.15	Fe K XANES

ELNES: energy loss near edge structure. XANES: X-ray absorption near edge structure.
