# Supplementary figures and images for: A dehydrated space-weathered skin cloaking the hydrated interior of Ryugu
Source: Nat Astron. 2022 Dec 19;7(2):170–81. doi: 10.1038/s41550-022-01841-6 (PMC9943745; doi:10.1038/s41550-022-01841-6)

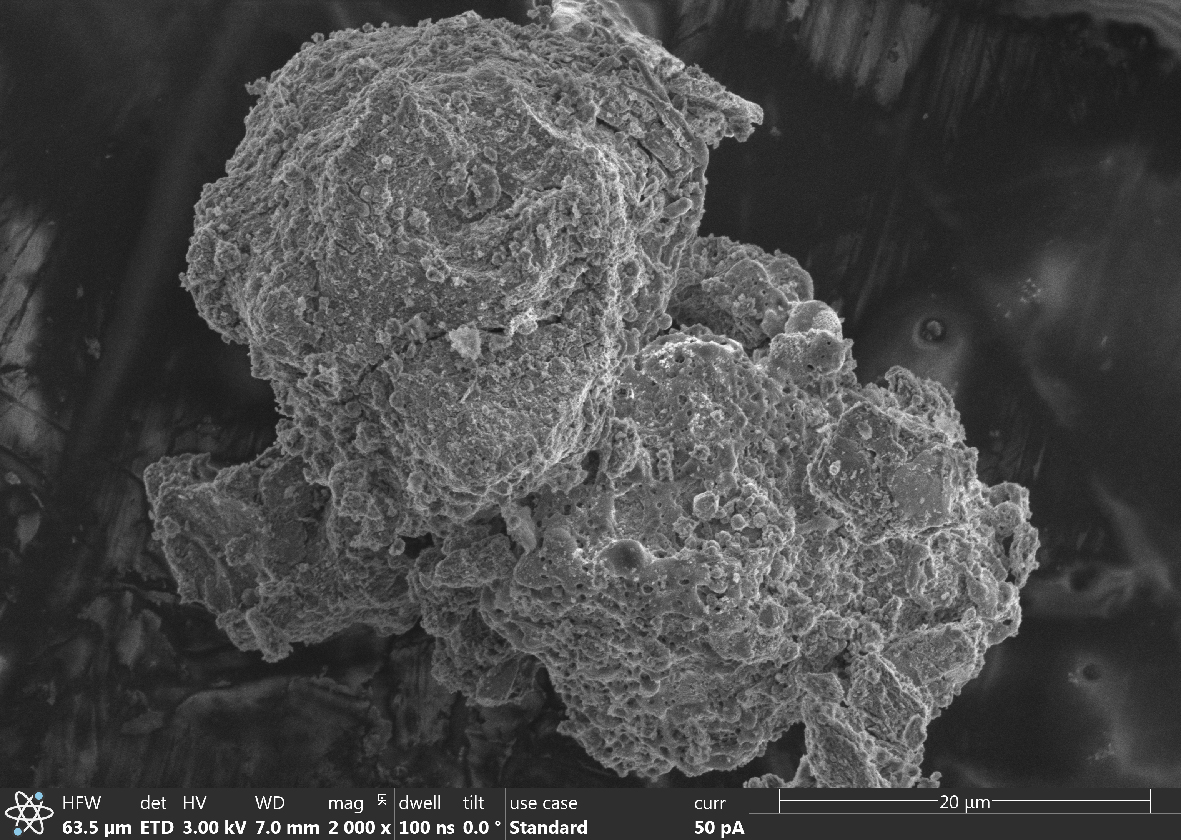

Supplement: Source Data Fig. 1 — Unprocessed images that were used in Fig. 1. [file 41550_2022_1841_MOESM2_ESM.zip › Source_Data_Fig.1/Fig.1_01.jpg]

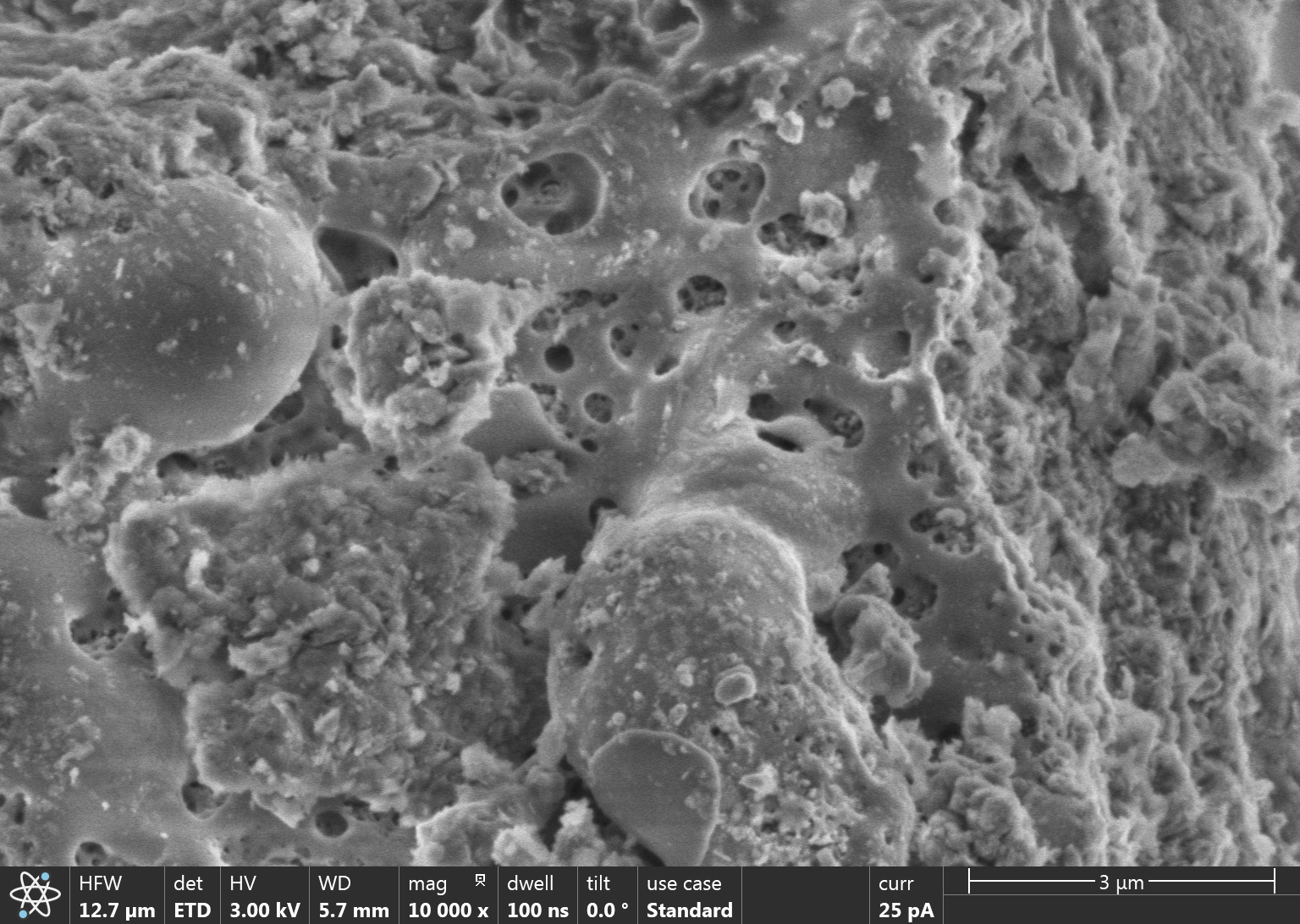

Supplement: Source Data Fig. 1 — Unprocessed images that were used in Fig. 1. [file 41550_2022_1841_MOESM2_ESM.zip › Source_Data_Fig.1/Fig.1_02.jpg]

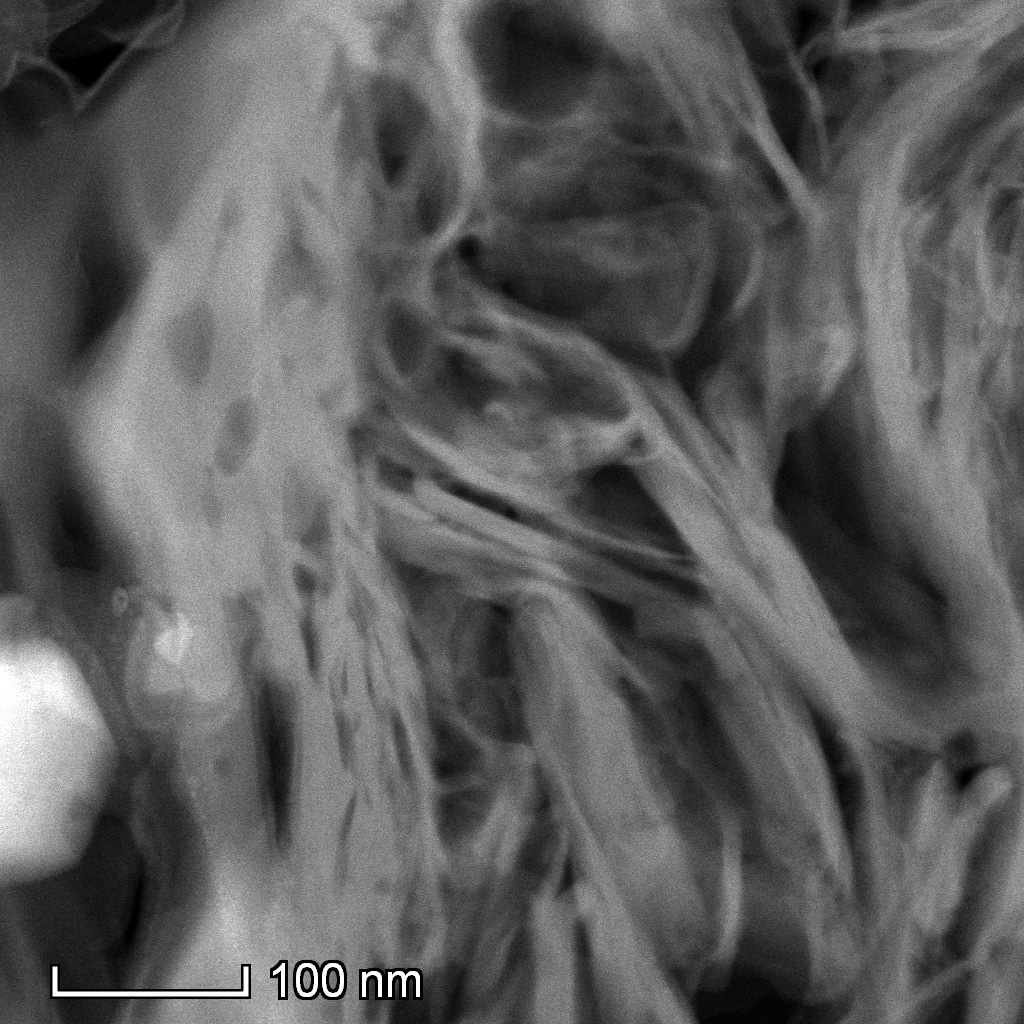

Supplement: Source Data Fig. 2 — Unprocessed images that were used in Fig. 2. [file 41550_2022_1841_MOESM3_ESM.zip › Source_Data_Fig2/Fig.2_01.jpg]

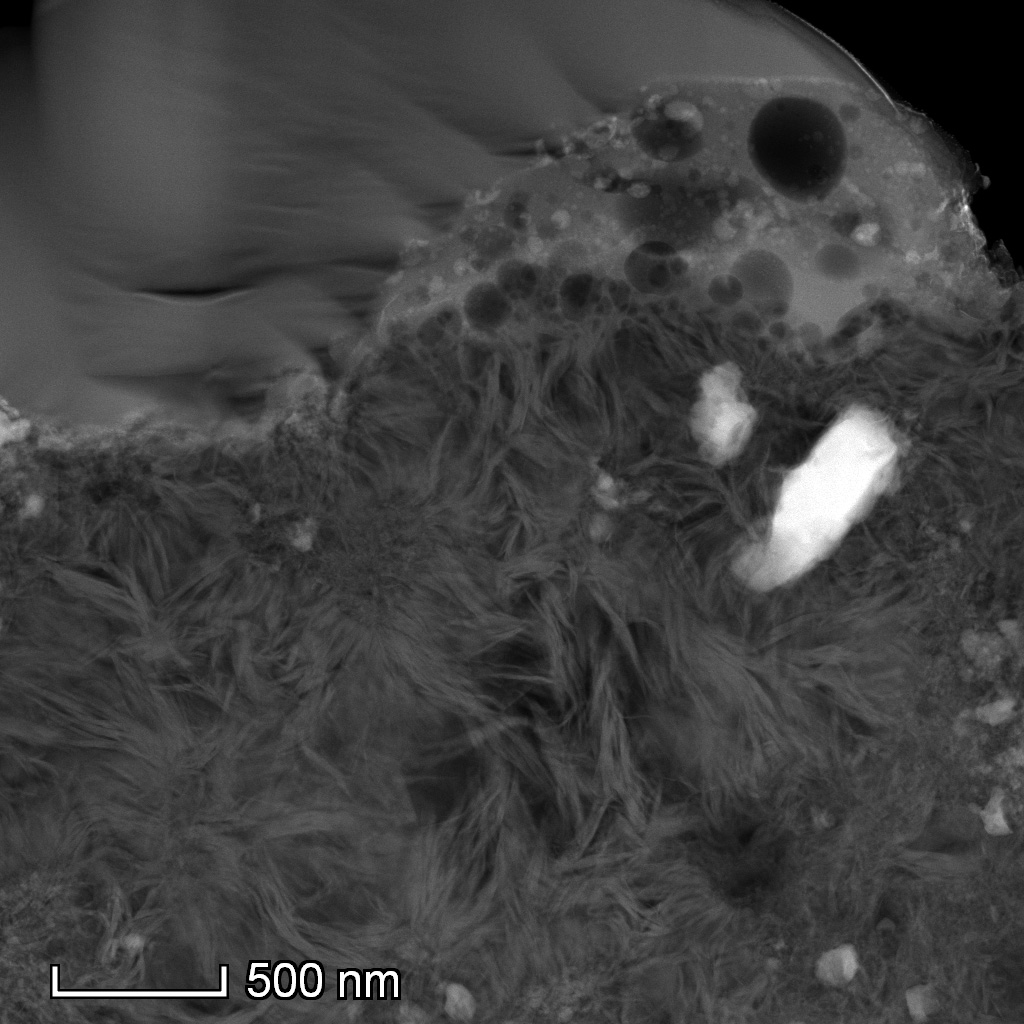

Supplement: Source Data Fig. 2 — Unprocessed images that were used in Fig. 2. [file 41550_2022_1841_MOESM3_ESM.zip › Source_Data_Fig2/Fig.2_02.jpg]

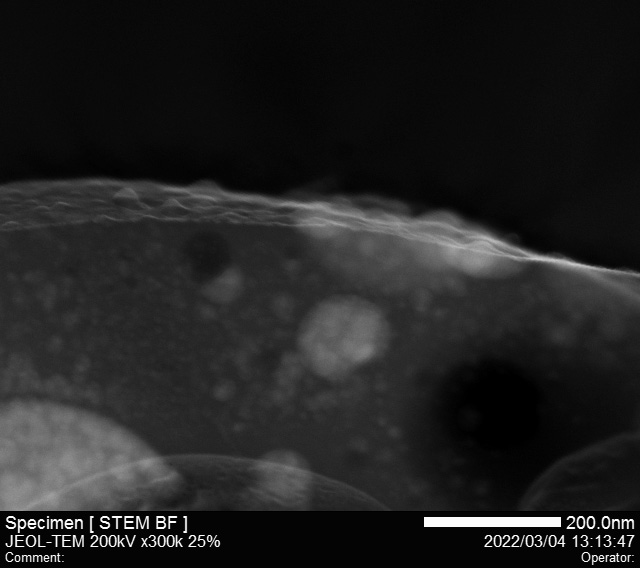

Supplement: Source Data Fig. 2 — Unprocessed images that were used in Fig. 2. [file 41550_2022_1841_MOESM3_ESM.zip › Source_Data_Fig2/Fig.2_03.jpg]

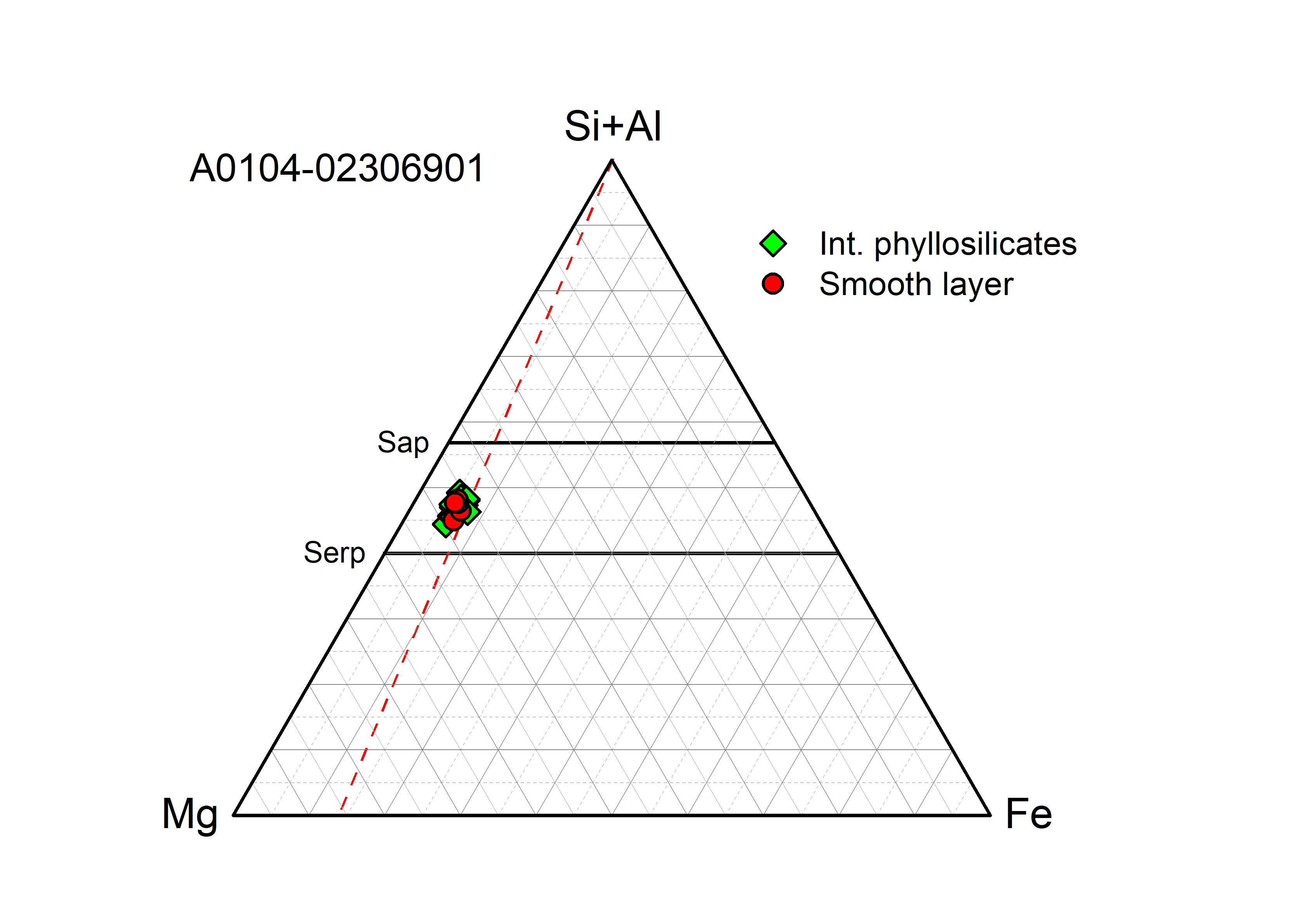

Supplement: Source Data Fig. 3 — Original graphs that were used in Fig. 3, and excel data to make these graphs. [file 41550_2022_1841_MOESM4_ESM.zip › Source_Data_Fig3/Fig.3_01.jpg]

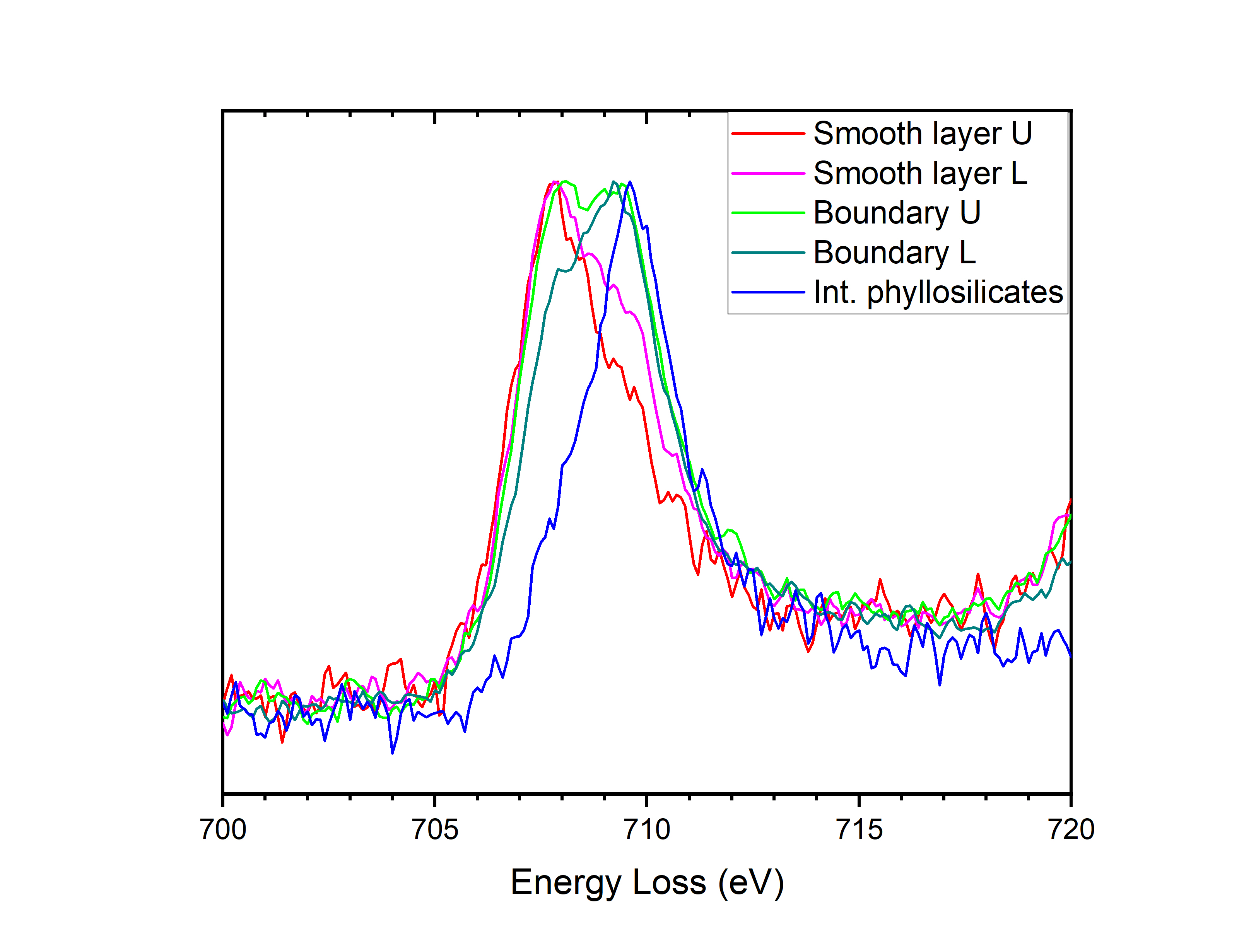

Supplement: Source Data Fig. 3 — Original graphs that were used in Fig. 3, and excel data to make these graphs. [file 41550_2022_1841_MOESM4_ESM.zip › Source_Data_Fig3/Fig.3_02.jpg]

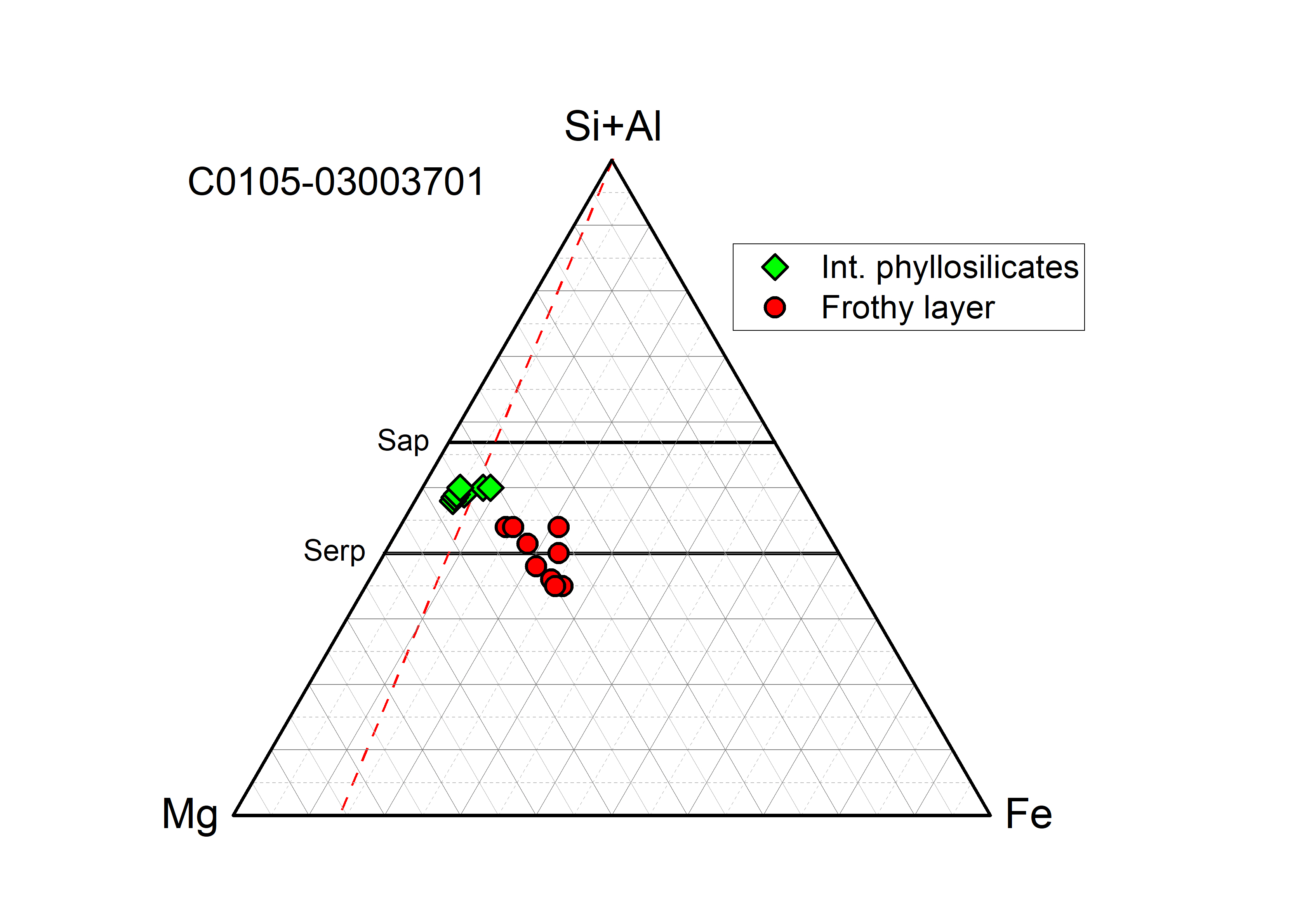

Supplement: Source Data Fig. 3 — Original graphs that were used in Fig. 3, and excel data to make these graphs. [file 41550_2022_1841_MOESM4_ESM.zip › Source_Data_Fig3/Fig.3_03.jpg]

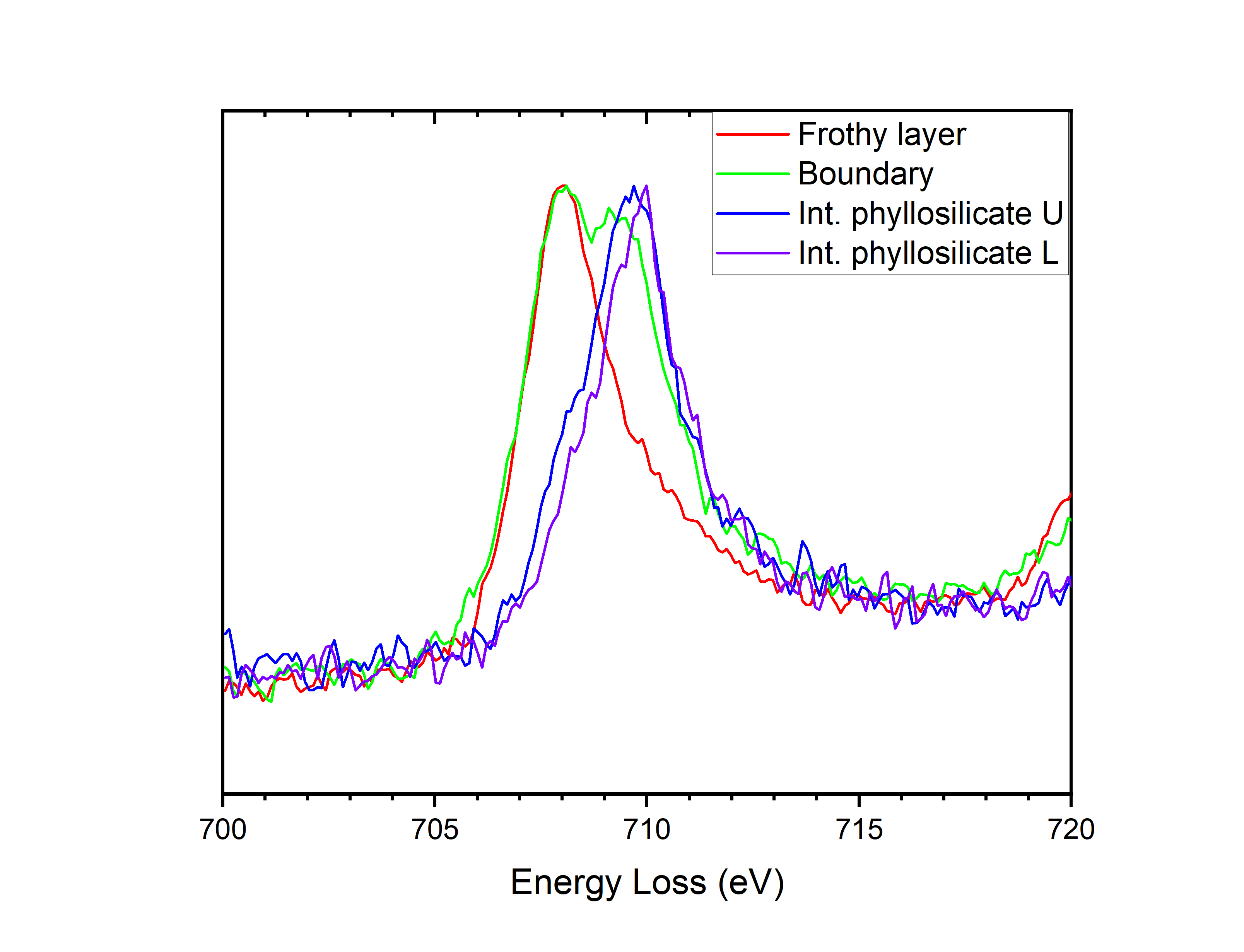

Supplement: Source Data Fig. 3 — Original graphs that were used in Fig. 3, and excel data to make these graphs. [file 41550_2022_1841_MOESM4_ESM.zip › Source_Data_Fig3/Fig.3_04.jpg]

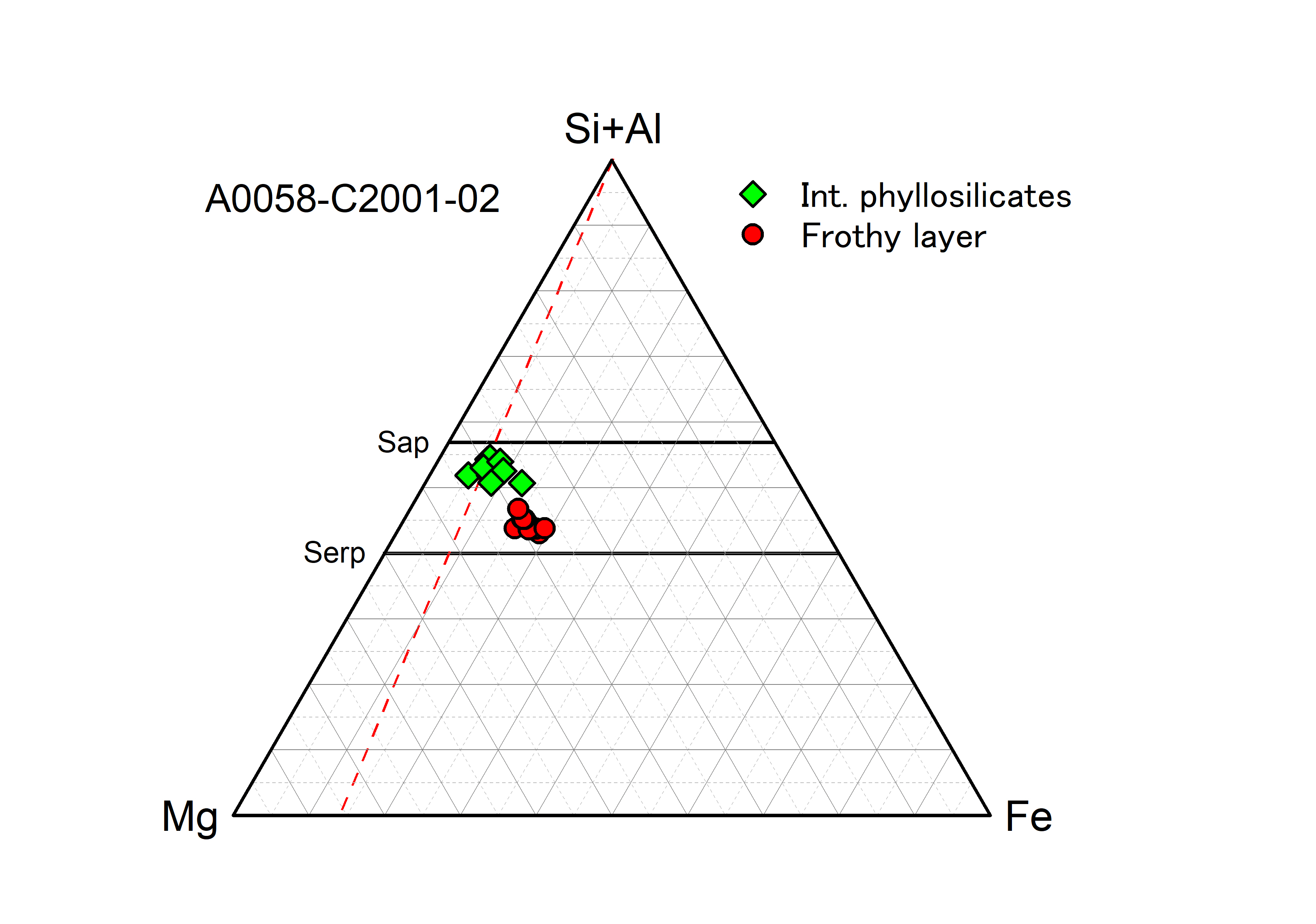

Supplement: Source Data Fig. 3 — Original graphs that were used in Fig. 3, and excel data to make these graphs. [file 41550_2022_1841_MOESM4_ESM.zip › Source_Data_Fig3/Fig.3_05.jpg]

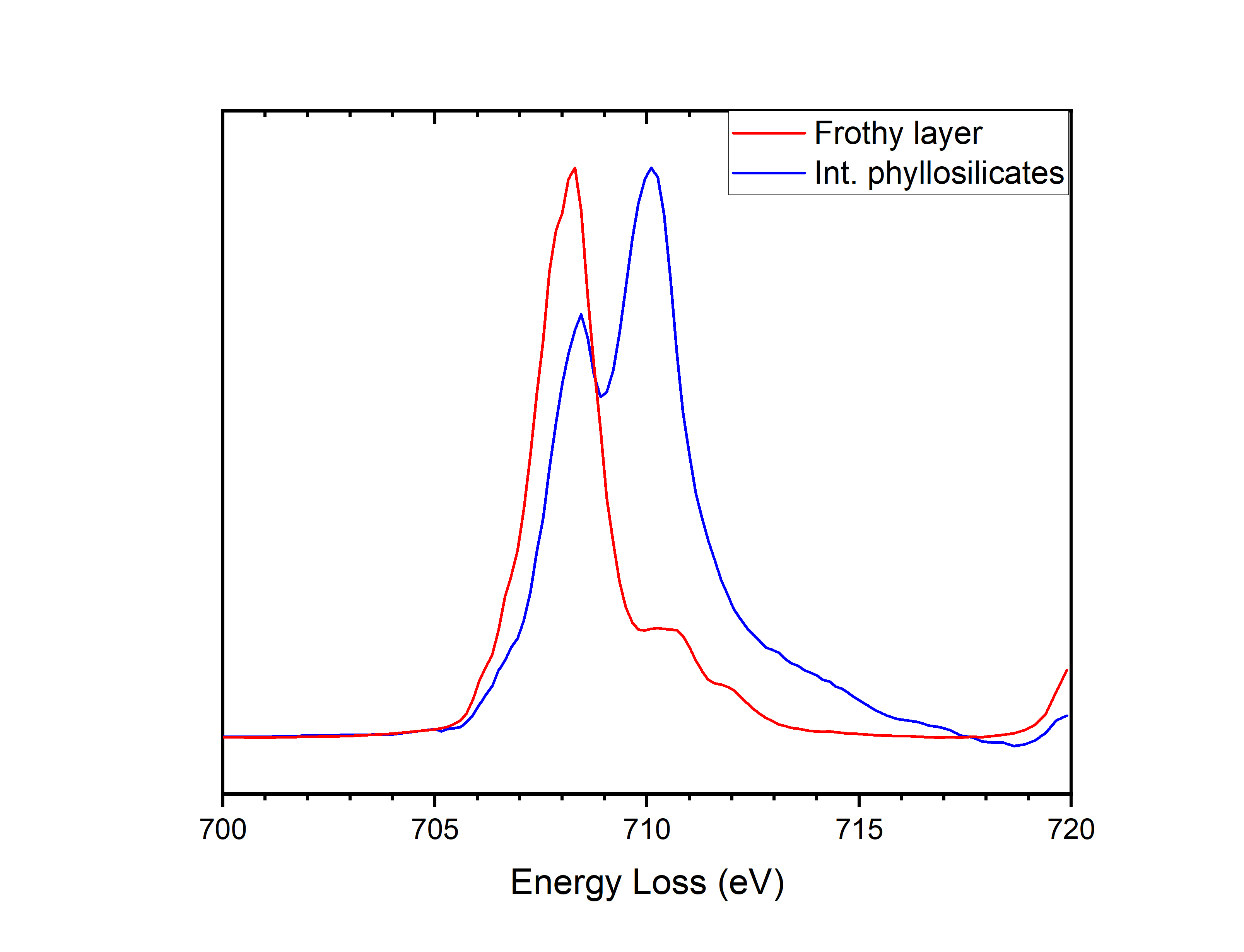

Supplement: Source Data Fig. 3 — Original graphs that were used in Fig. 3, and excel data to make these graphs. [file 41550_2022_1841_MOESM4_ESM.zip › Source_Data_Fig3/Fig.3_06.jpg]

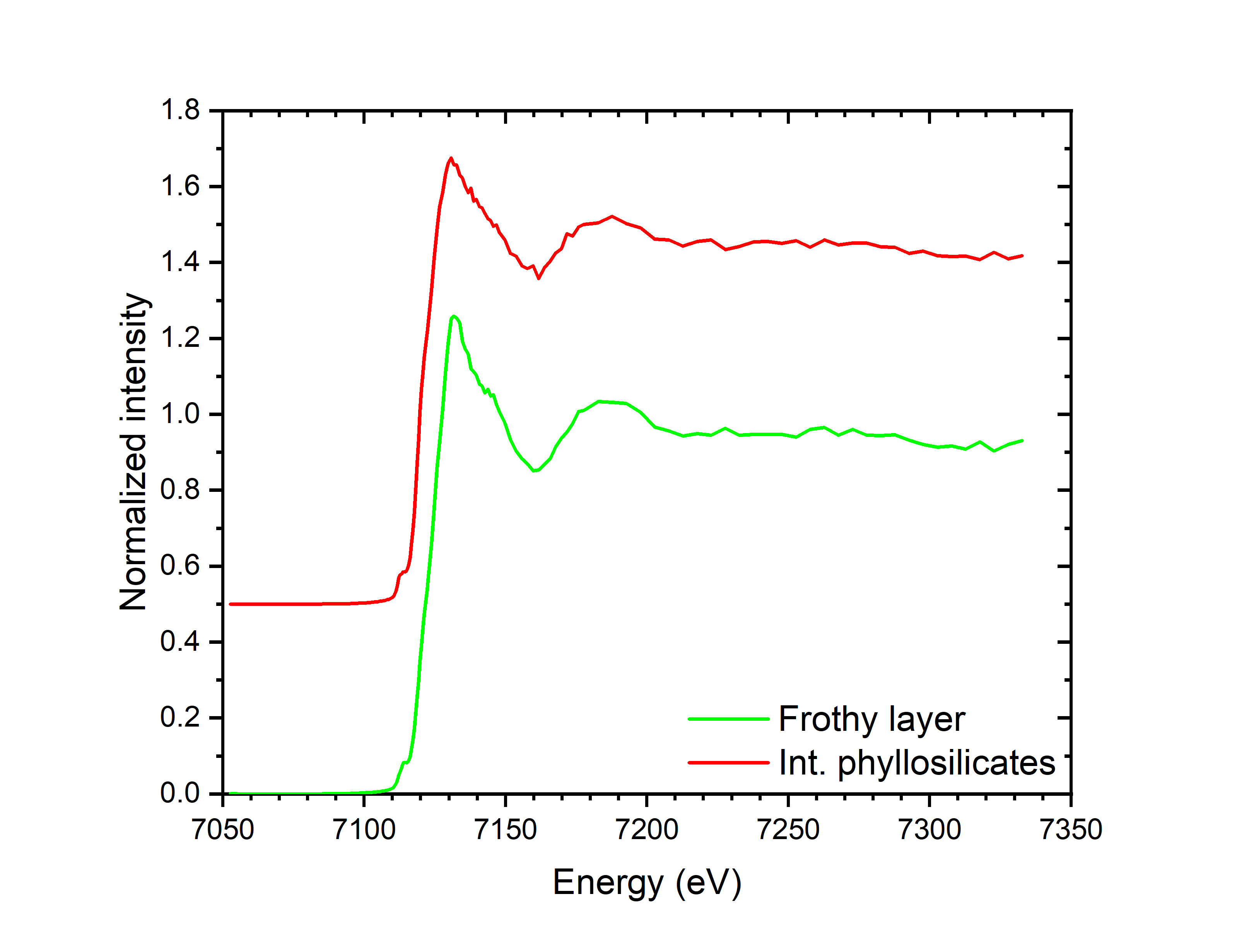

Supplement: Source Data Fig. 3 — Original graphs that were used in Fig. 3, and excel data to make these graphs. [file 41550_2022_1841_MOESM4_ESM.zip › Source_Data_Fig3/Fig.3_07.jpg]

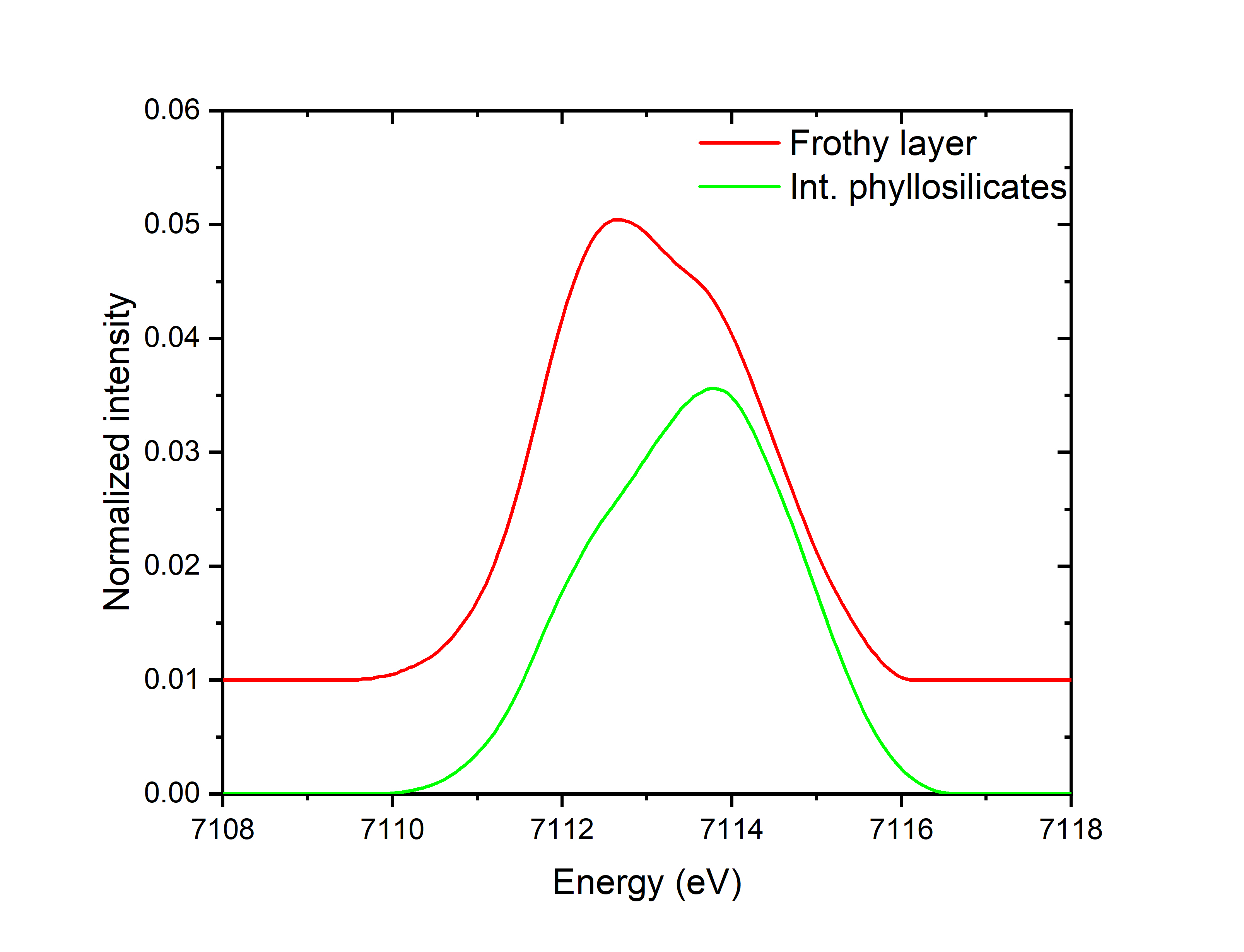

Supplement: Source Data Fig. 3 — Original graphs that were used in Fig. 3, and excel data to make these graphs. [file 41550_2022_1841_MOESM4_ESM.zip › Source_Data_Fig3/Fig.3_08.jpg]

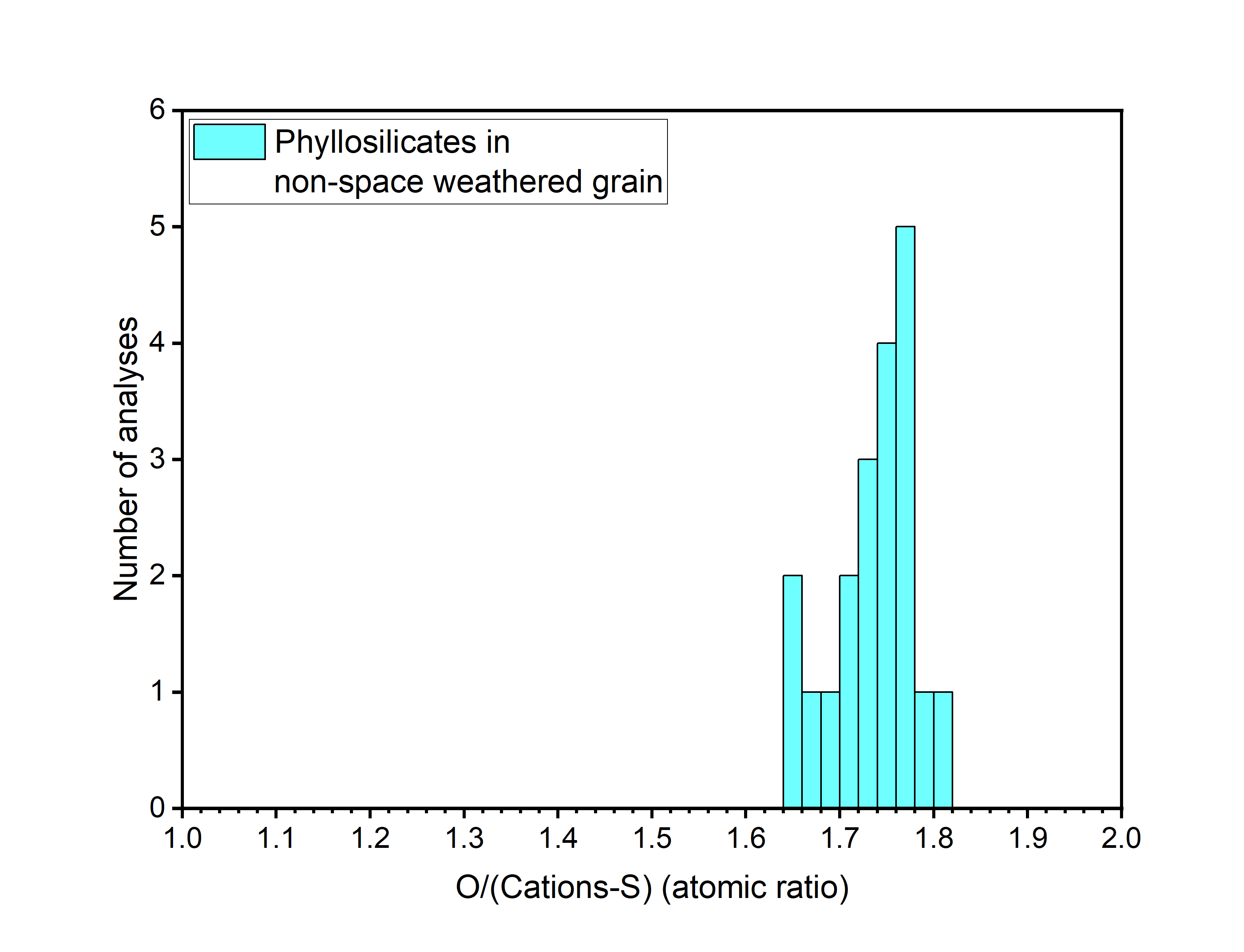

Supplement: Source Data Fig. 4 — Original graphs that were used in Fig. 4, and excel data to make these graphs. [file 41550_2022_1841_MOESM5_ESM.zip › Source_Data_Fig4/Fig.4_01_hist_A0104-01500401.jpg]

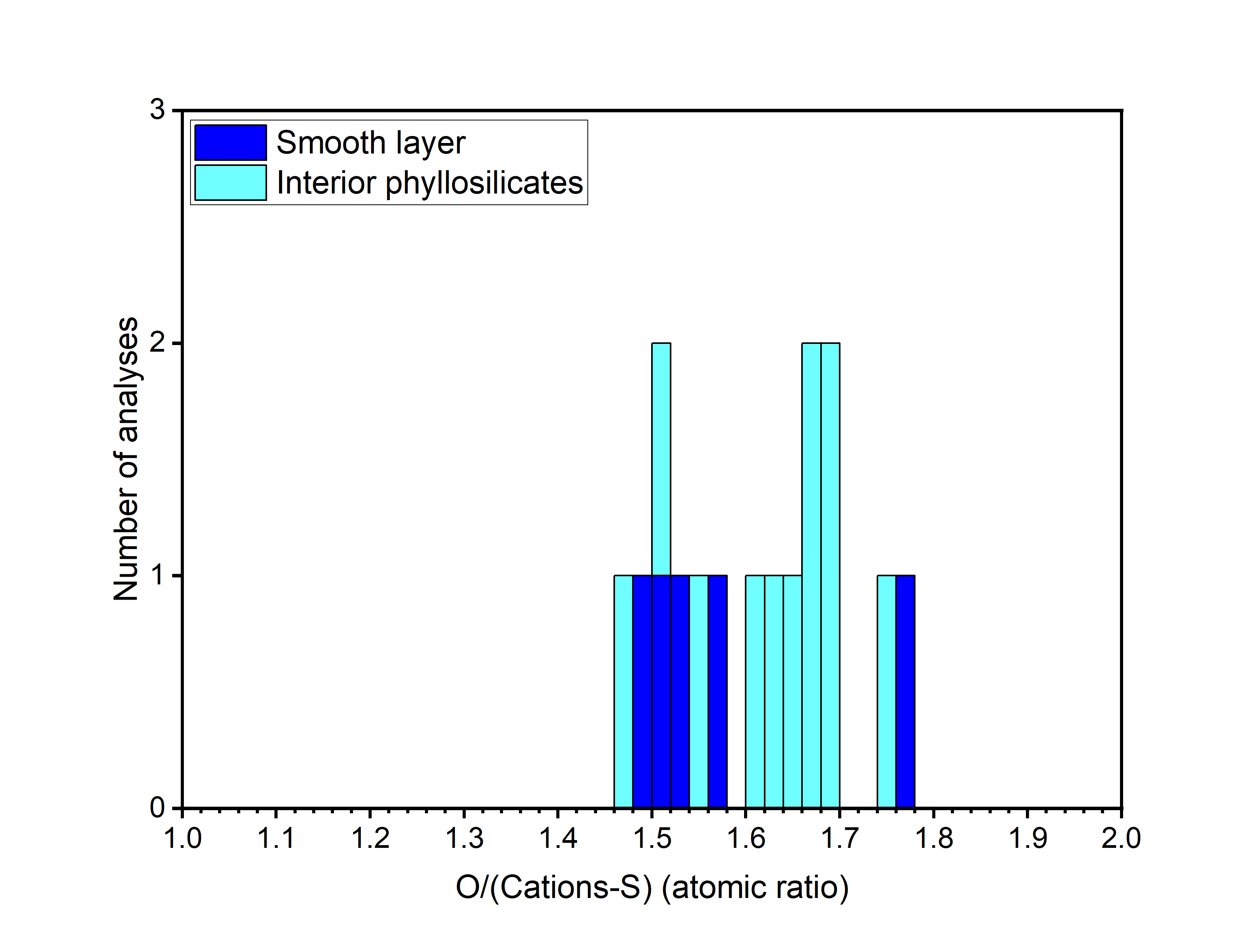

Supplement: Source Data Fig. 4 — Original graphs that were used in Fig. 4, and excel data to make these graphs. [file 41550_2022_1841_MOESM5_ESM.zip › Source_Data_Fig4/Fig.4_02_hist_A0104-02306901.jpg]

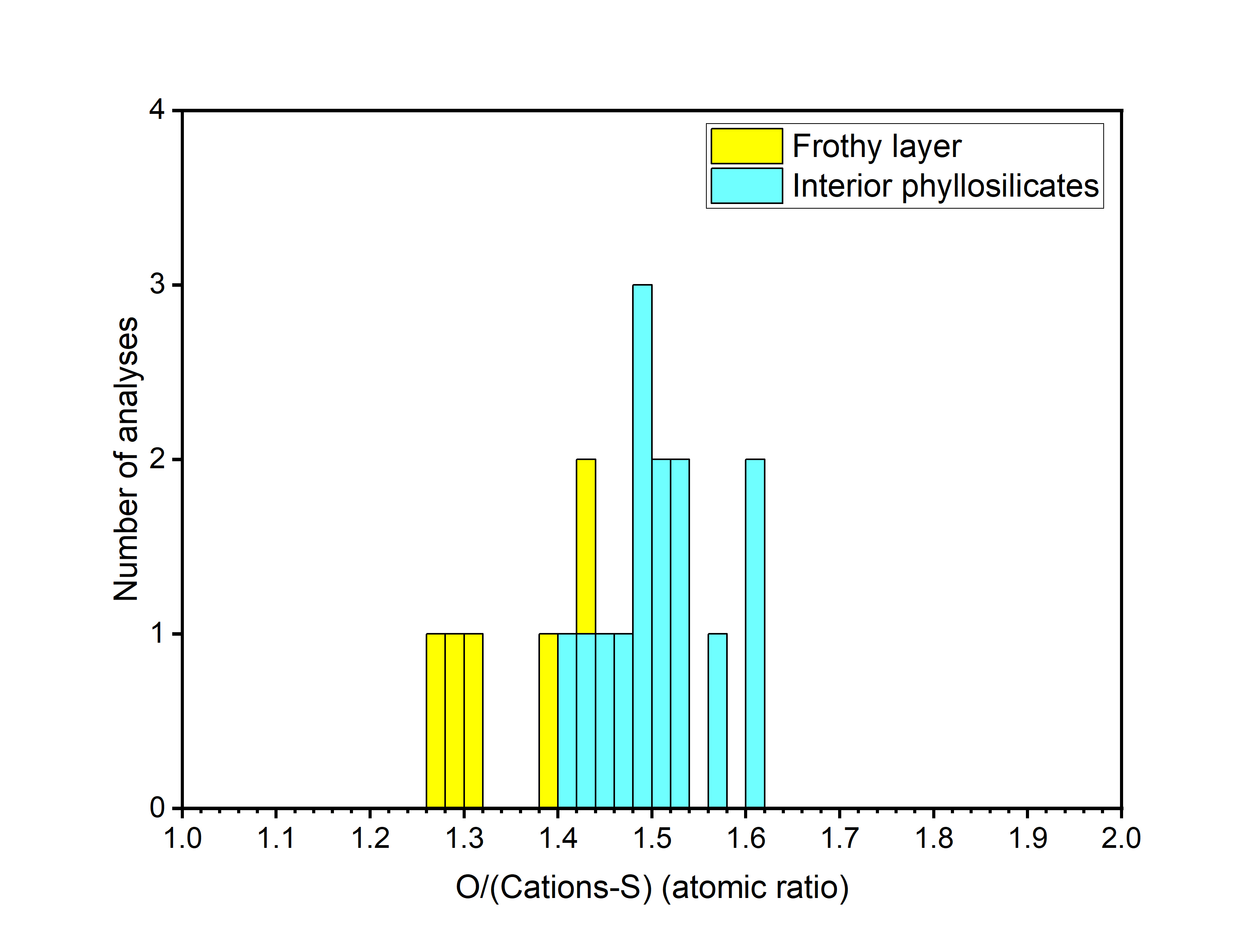

Supplement: Source Data Fig. 4 — Original graphs that were used in Fig. 4, and excel data to make these graphs. [file 41550_2022_1841_MOESM5_ESM.zip › Source_Data_Fig4/Fig.4_03_hist_A0104-02203703.jpg]

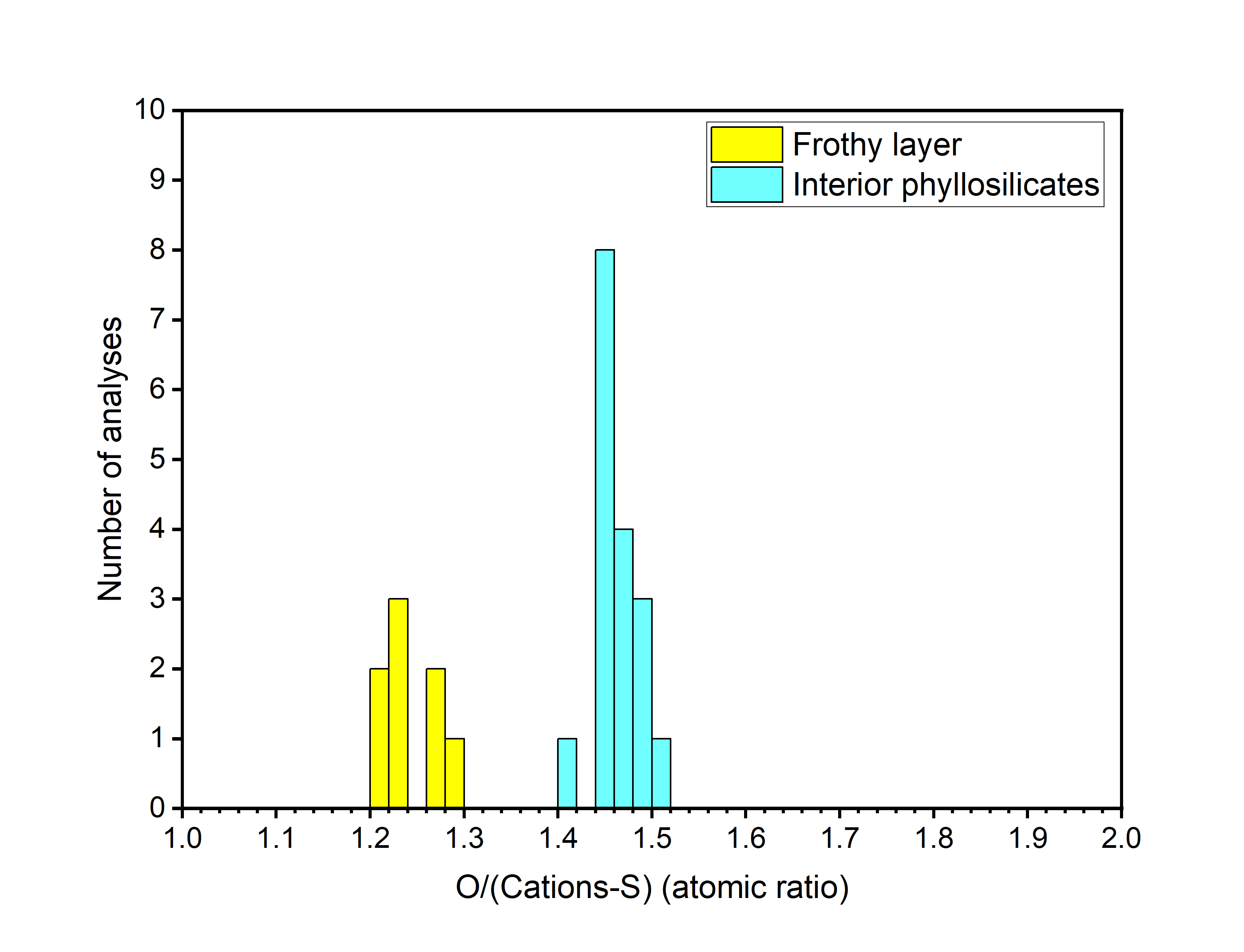

Supplement: Source Data Fig. 4 — Original graphs that were used in Fig. 4, and excel data to make these graphs. [file 41550_2022_1841_MOESM5_ESM.zip › Source_Data_Fig4/Fig.4_04_hist_C0105-03003701.jpg]

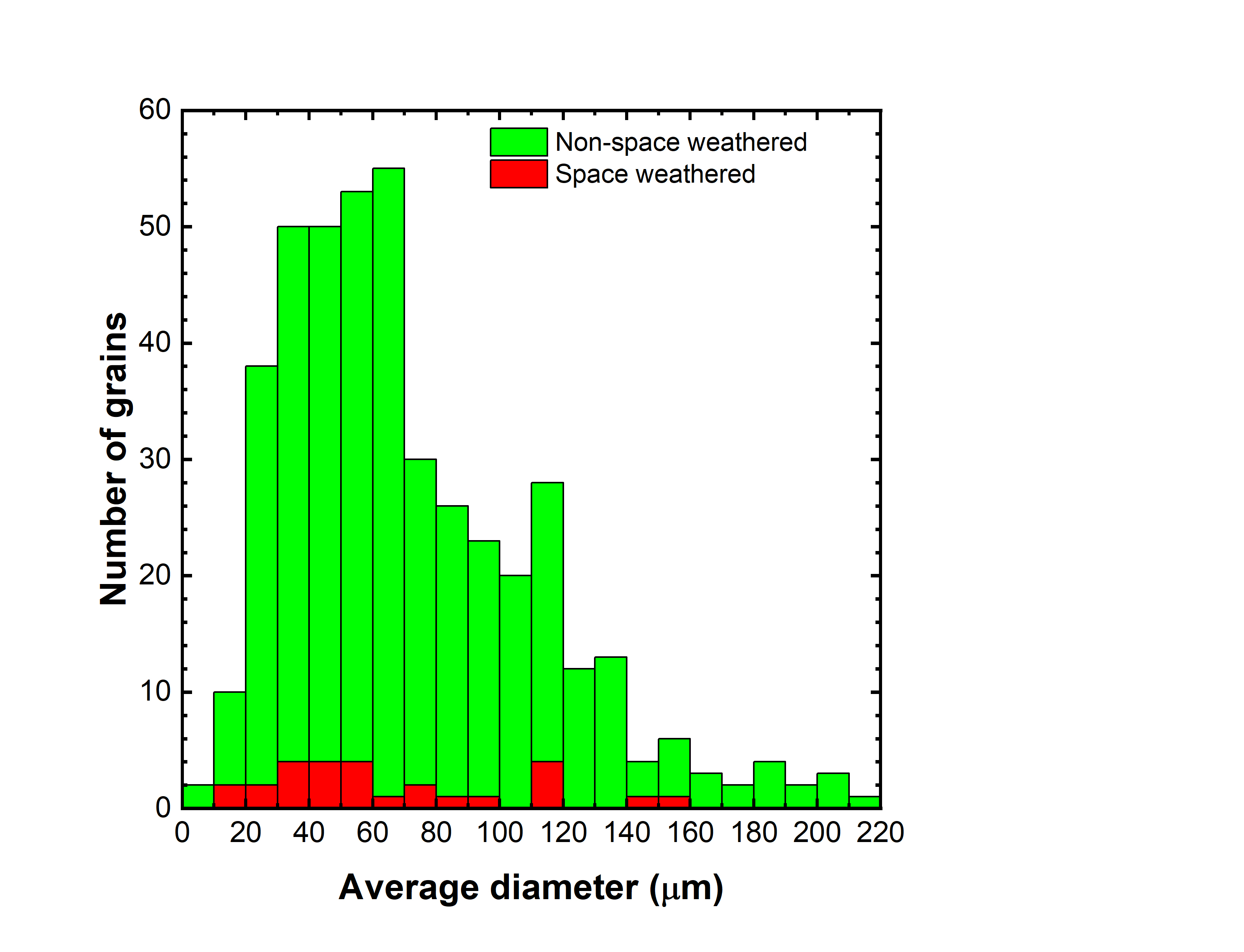

Supplement: Source Data Extended Data Fig. 1 — Original graphs that were used in Extended Data Fig. 1, and excel data to make these graphs. [file 41550_2022_1841_MOESM6_ESM.zip › Source_Data_ED_Fig1/EDFig.1_01.jpg]

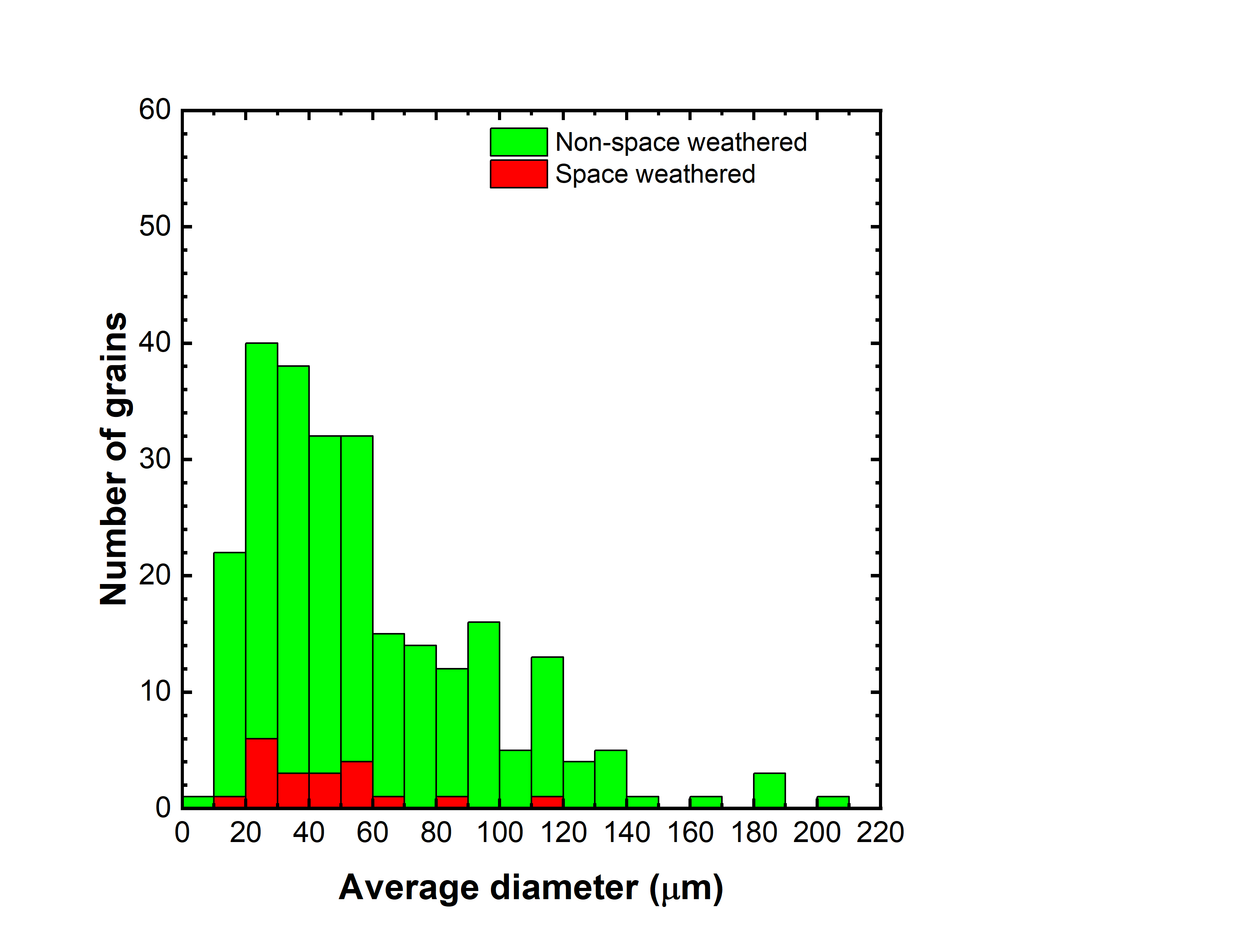

Supplement: Source Data Extended Data Fig. 1 — Original graphs that were used in Extended Data Fig. 1, and excel data to make these graphs. [file 41550_2022_1841_MOESM6_ESM.zip › Source_Data_ED_Fig1/EDFig.1_02.jpg]

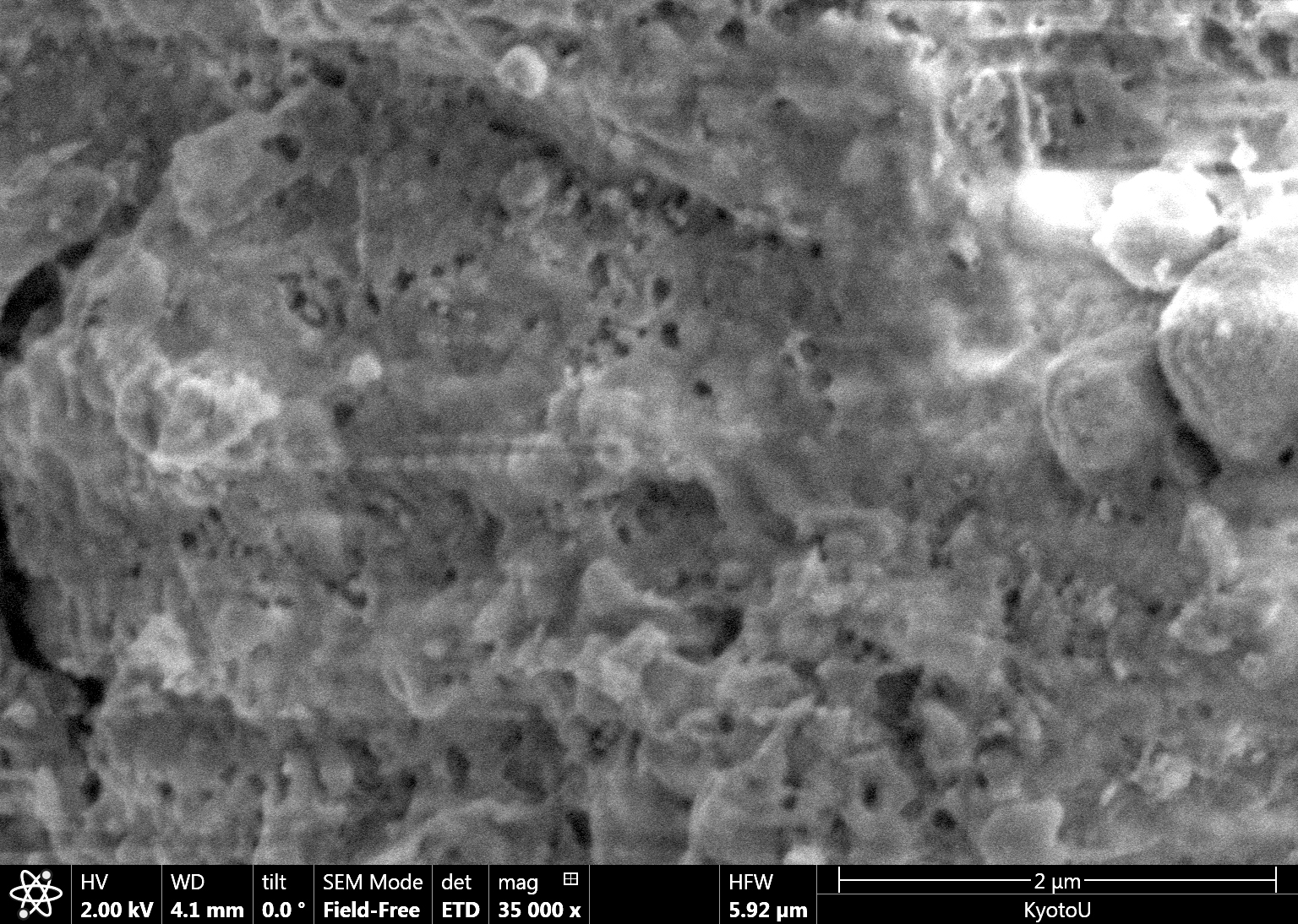

Supplement: Source Data Extended Data Fig. 2 — Unprocessed images that were used in Extended Data Fig. 2. [file 41550_2022_1841_MOESM7_ESM.zip › Source_Data_ED_Fig2/ED_Fig.2_01.jpg]

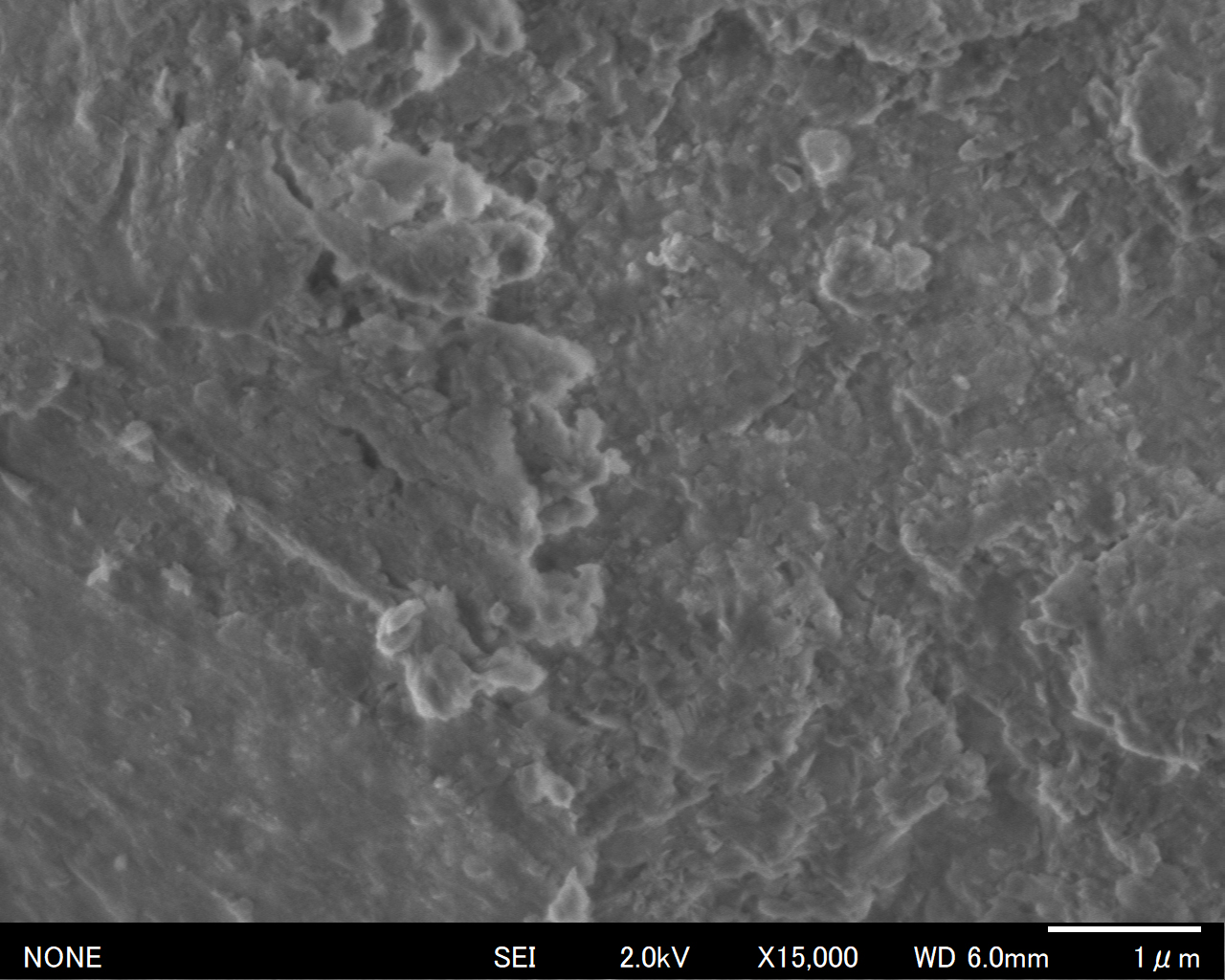

Supplement: Source Data Extended Data Fig. 2 — Unprocessed images that were used in Extended Data Fig. 2. [file 41550_2022_1841_MOESM7_ESM.zip › Source_Data_ED_Fig2/ED_Fig.2_02.jpg]

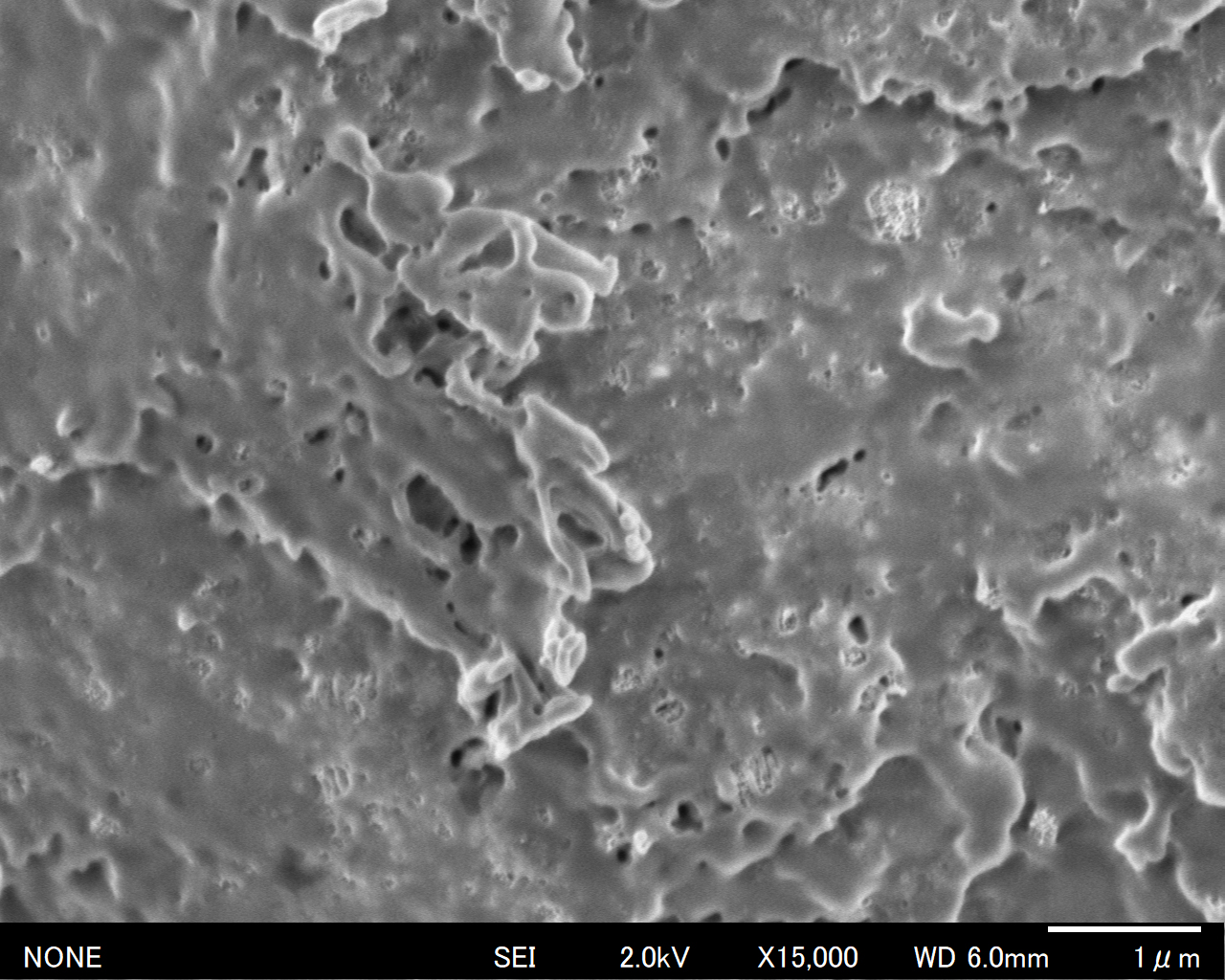

Supplement: Source Data Extended Data Fig. 2 — Unprocessed images that were used in Extended Data Fig. 2. [file 41550_2022_1841_MOESM7_ESM.zip › Source_Data_ED_Fig2/ED_Fig.2_03.jpg]

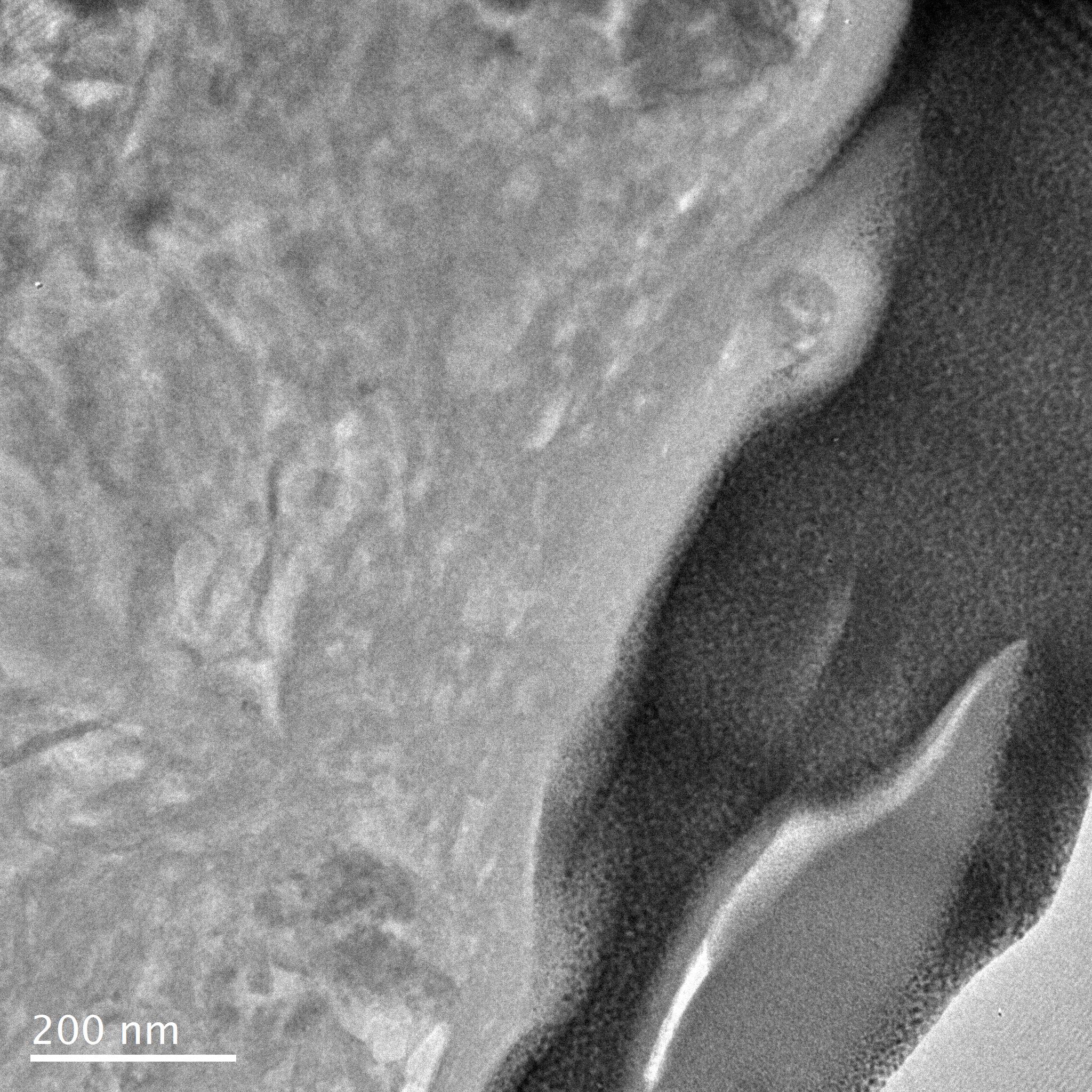

Supplement: Source Data Extended Data Fig. 2 — Unprocessed images that were used in Extended Data Fig. 2. [file 41550_2022_1841_MOESM7_ESM.zip › Source_Data_ED_Fig2/ED_Fig.2_04.jpg]

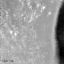

Supplement: Source Data Extended Data Fig. 2 — Unprocessed images that were used in Extended Data Fig. 2. [file 41550_2022_1841_MOESM7_ESM.zip › Source_Data_ED_Fig2/ED_Fig.2_05.jpg]

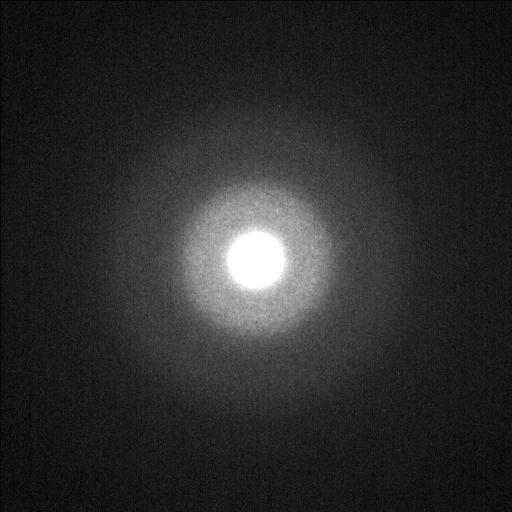

Supplement: Source Data Extended Data Fig. 2 — Unprocessed images that were used in Extended Data Fig. 2. [file 41550_2022_1841_MOESM7_ESM.zip › Source_Data_ED_Fig2/ED_Fig.2_06.jpg]

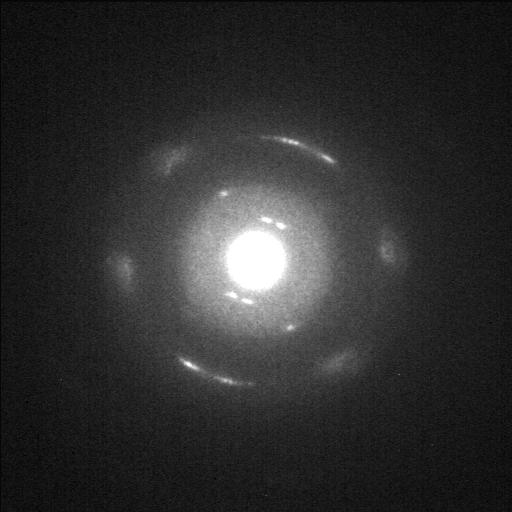

Supplement: Source Data Extended Data Fig. 2 — Unprocessed images that were used in Extended Data Fig. 2. [file 41550_2022_1841_MOESM7_ESM.zip › Source_Data_ED_Fig2/ED_Fig.2_07.jpg]

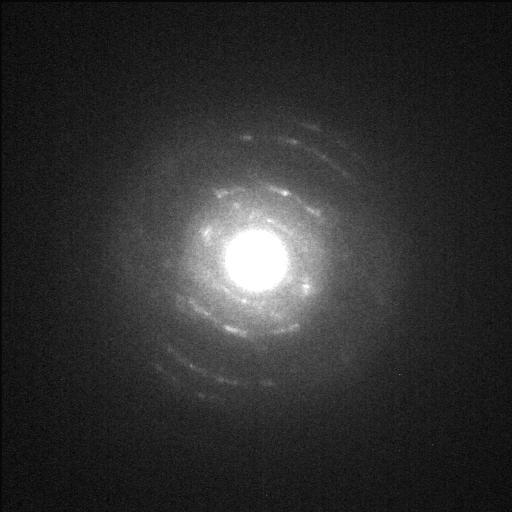

Supplement: Source Data Extended Data Fig. 2 — Unprocessed images that were used in Extended Data Fig. 2. [file 41550_2022_1841_MOESM7_ESM.zip › Source_Data_ED_Fig2/ED_Fig.2_08.jpg]

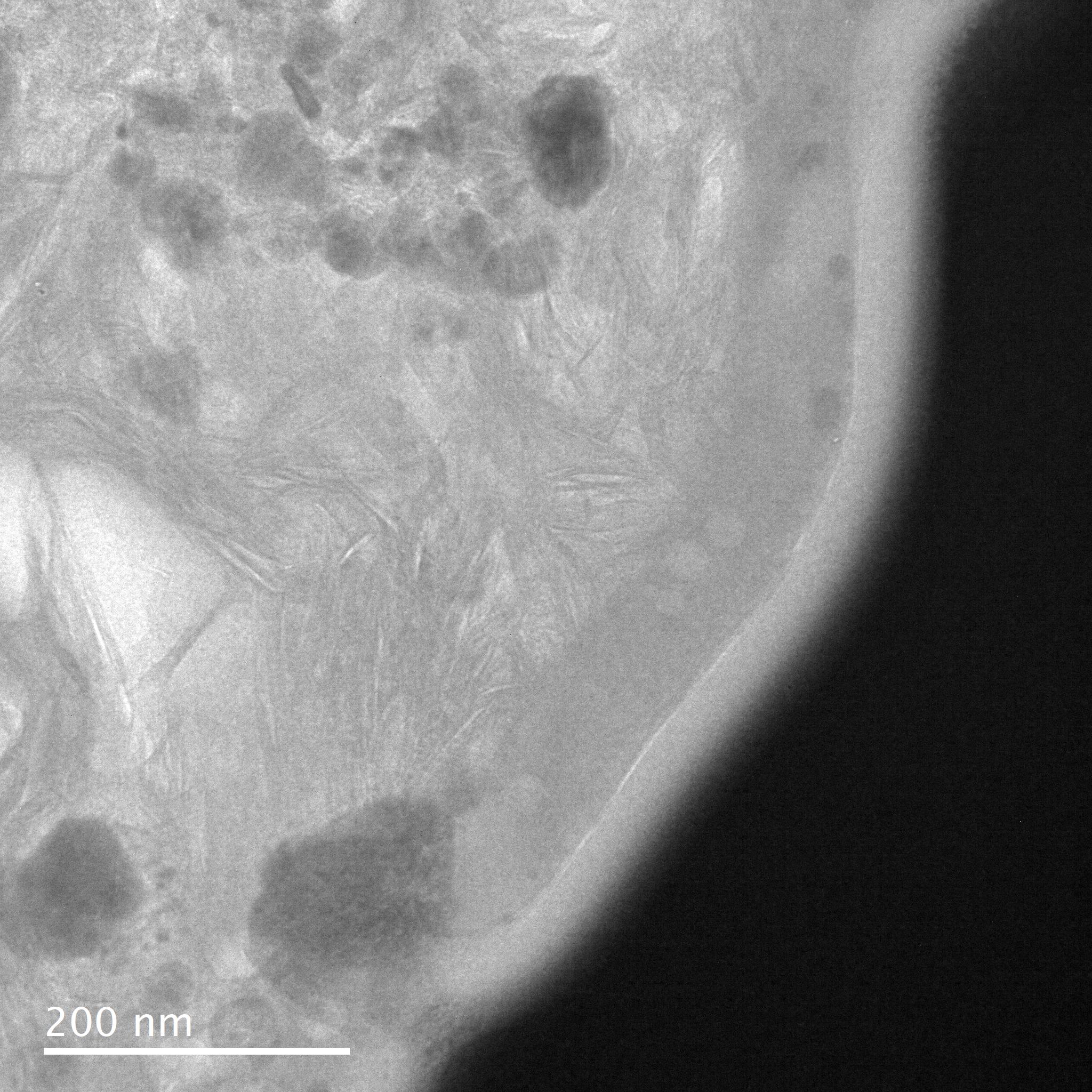

Supplement: Source Data Extended Data Fig. 2 — Unprocessed images that were used in Extended Data Fig. 2. [file 41550_2022_1841_MOESM7_ESM.zip › Source_Data_ED_Fig2/ED_Fig.2_09.jpg]

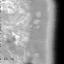

Supplement: Source Data Extended Data Fig. 2 — Unprocessed images that were used in Extended Data Fig. 2. [file 41550_2022_1841_MOESM7_ESM.zip › Source_Data_ED_Fig2/ED_Fig.2_10.jpg]

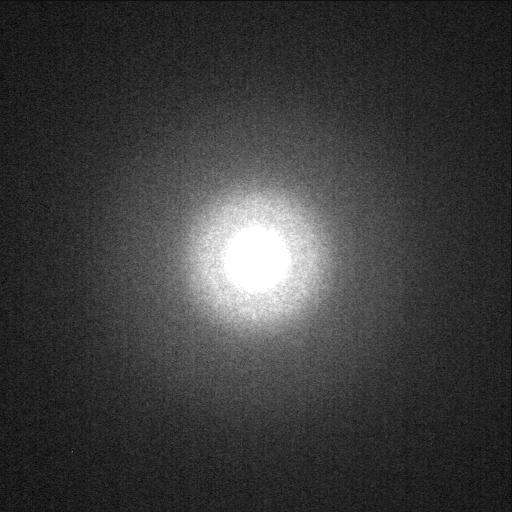

Supplement: Source Data Extended Data Fig. 2 — Unprocessed images that were used in Extended Data Fig. 2. [file 41550_2022_1841_MOESM7_ESM.zip › Source_Data_ED_Fig2/ED_Fig.2_11.jpg]

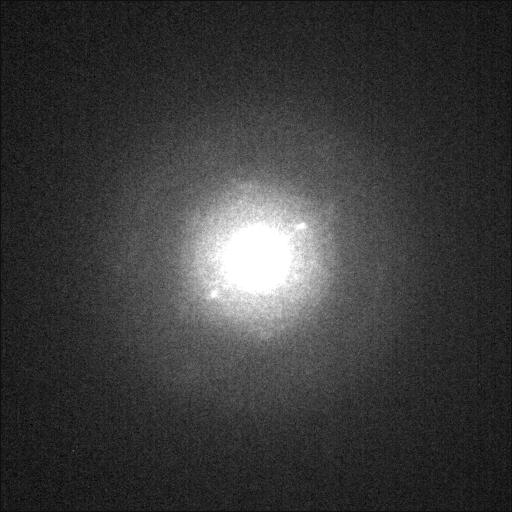

Supplement: Source Data Extended Data Fig. 2 — Unprocessed images that were used in Extended Data Fig. 2. [file 41550_2022_1841_MOESM7_ESM.zip › Source_Data_ED_Fig2/ED_Fig.2_12.jpg]

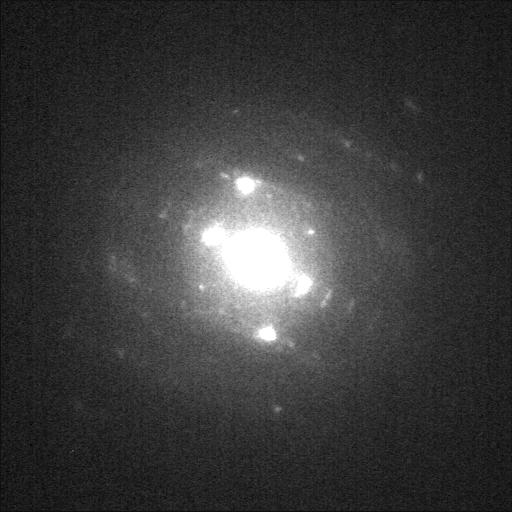

Supplement: Source Data Extended Data Fig. 2 — Unprocessed images that were used in Extended Data Fig. 2. [file 41550_2022_1841_MOESM7_ESM.zip › Source_Data_ED_Fig2/ED_Fig.2_13.jpg]

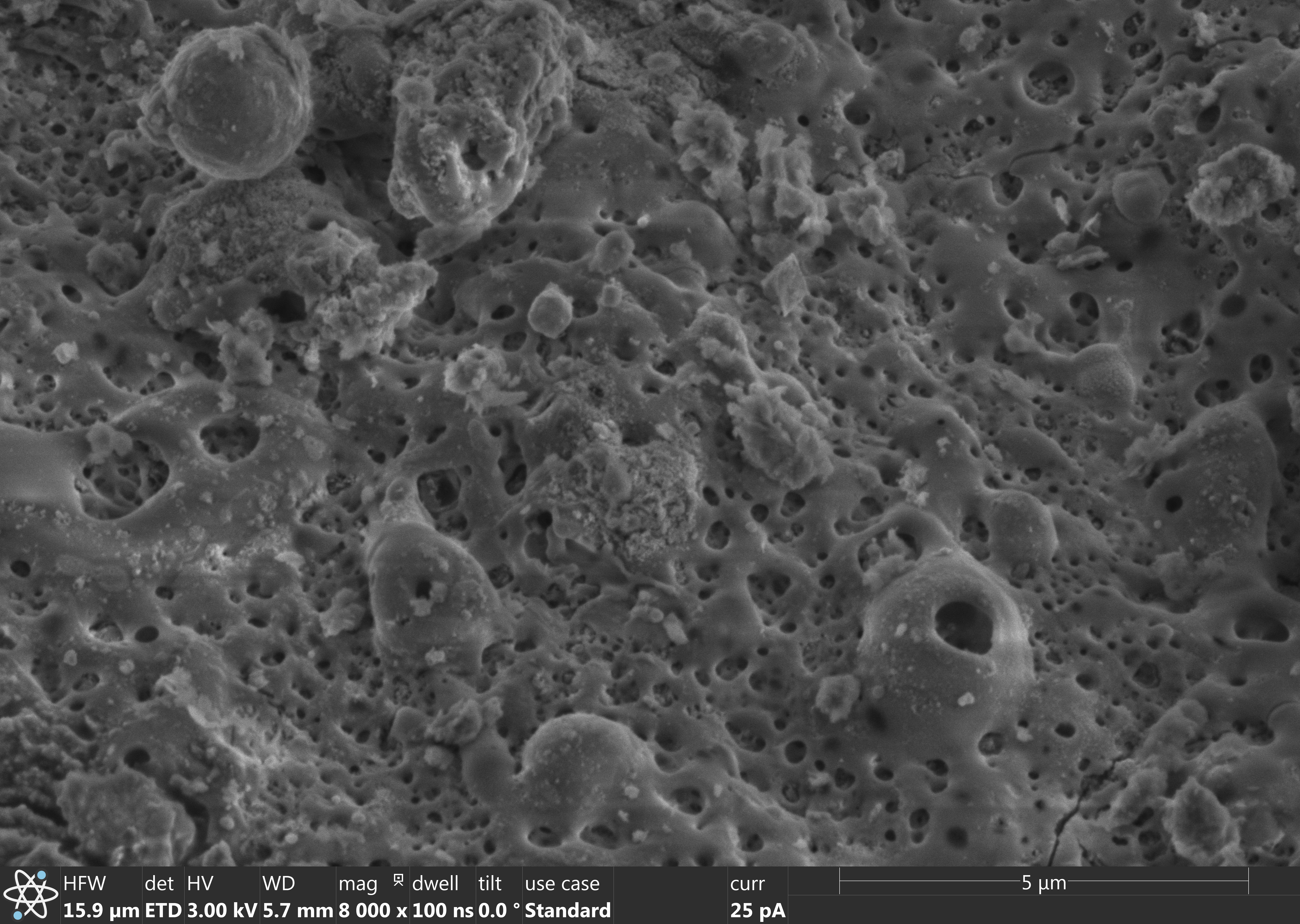

Supplement: Source Data Extended Data Fig. 3 — Unprocessed images that were used in Extended Data Fig. 3. [file 41550_2022_1841_MOESM8_ESM.zip › Source_Data_ED_Fig3/ED_Fig.3_01.jpg]

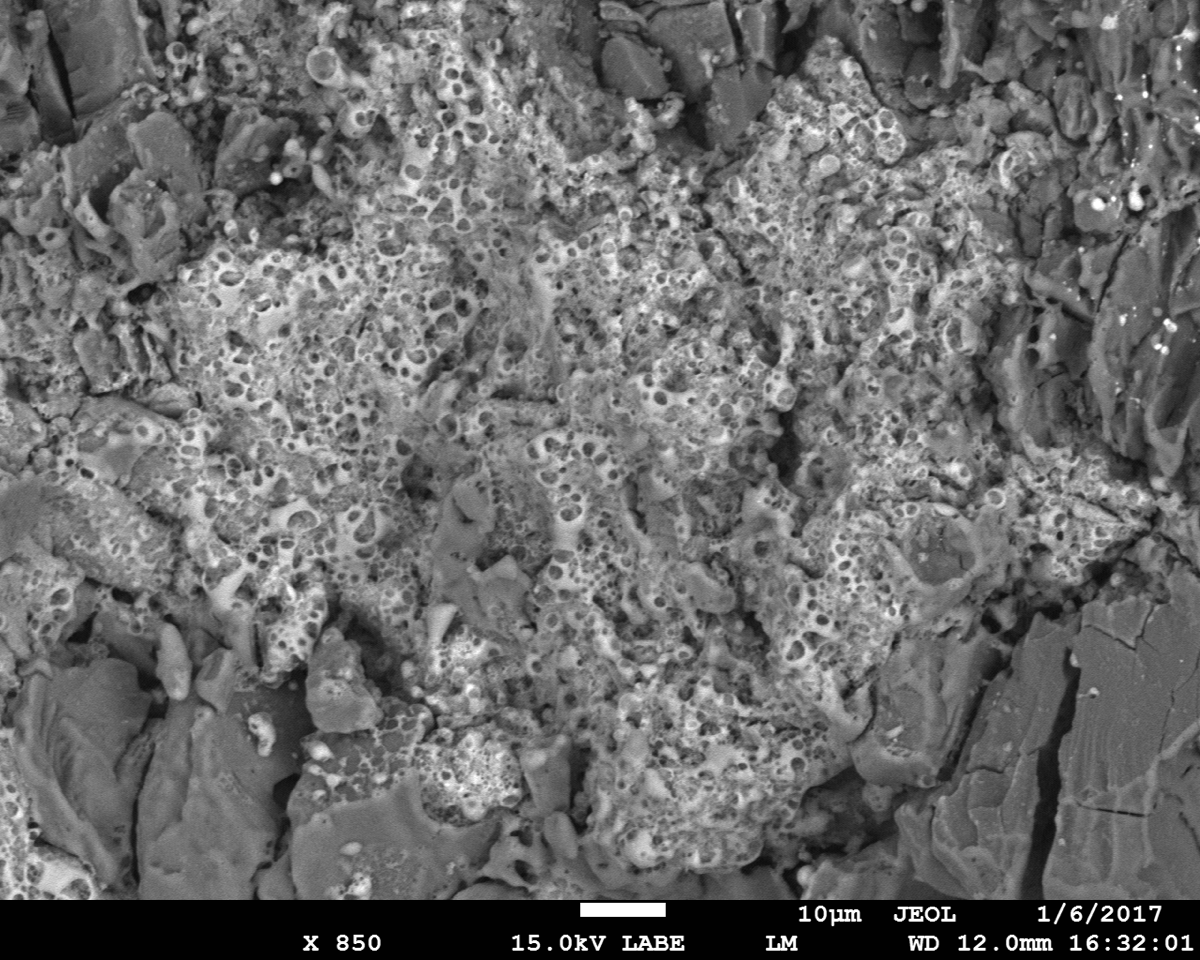

Supplement: Source Data Extended Data Fig. 3 — Unprocessed images that were used in Extended Data Fig. 3. [file 41550_2022_1841_MOESM8_ESM.zip › Source_Data_ED_Fig3/ED_Fig.3_02.tiff]

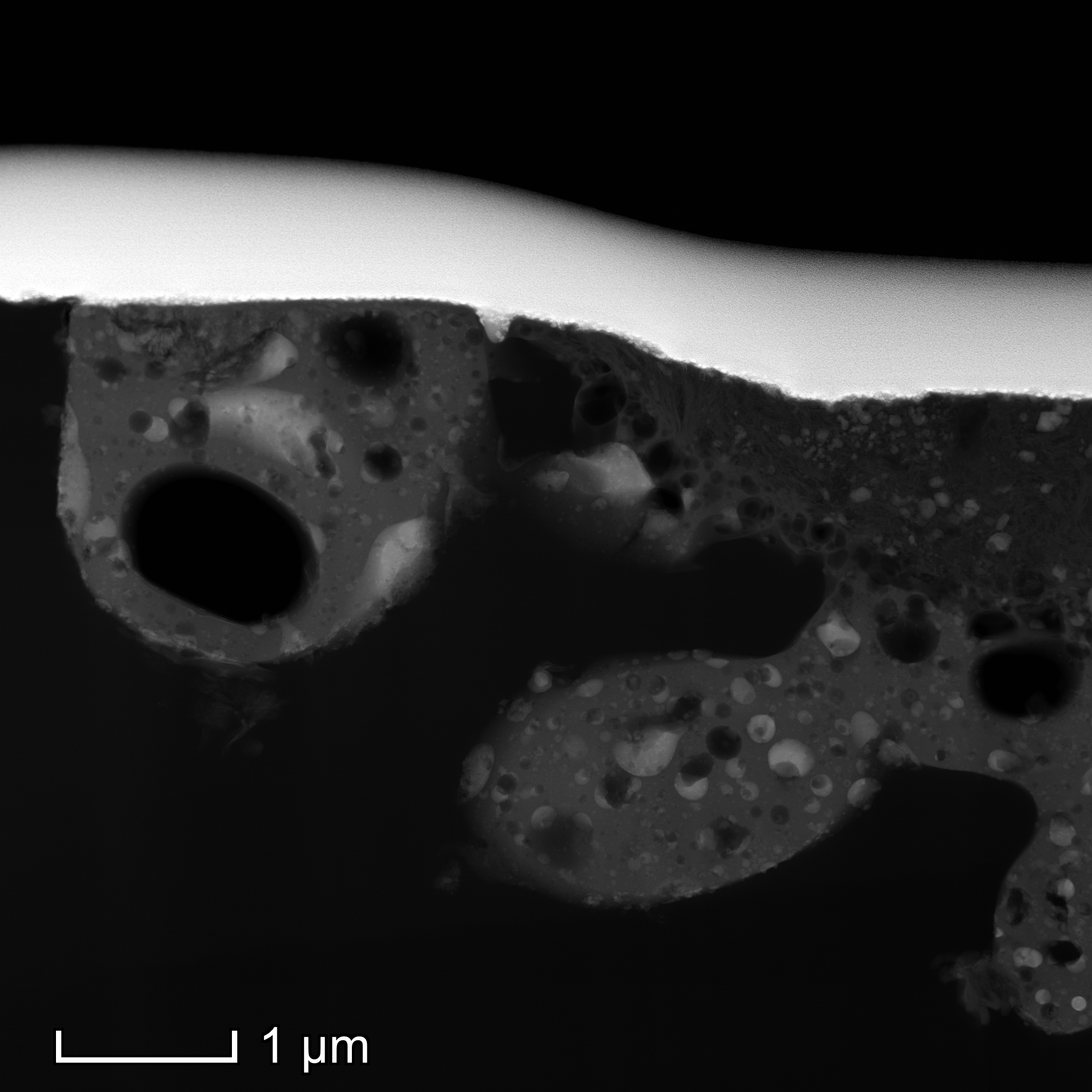

Supplement: Source Data Extended Data Fig. 3 — Unprocessed images that were used in Extended Data Fig. 3. [file 41550_2022_1841_MOESM8_ESM.zip › Source_Data_ED_Fig3/ED_Fig.3_03a.tif]

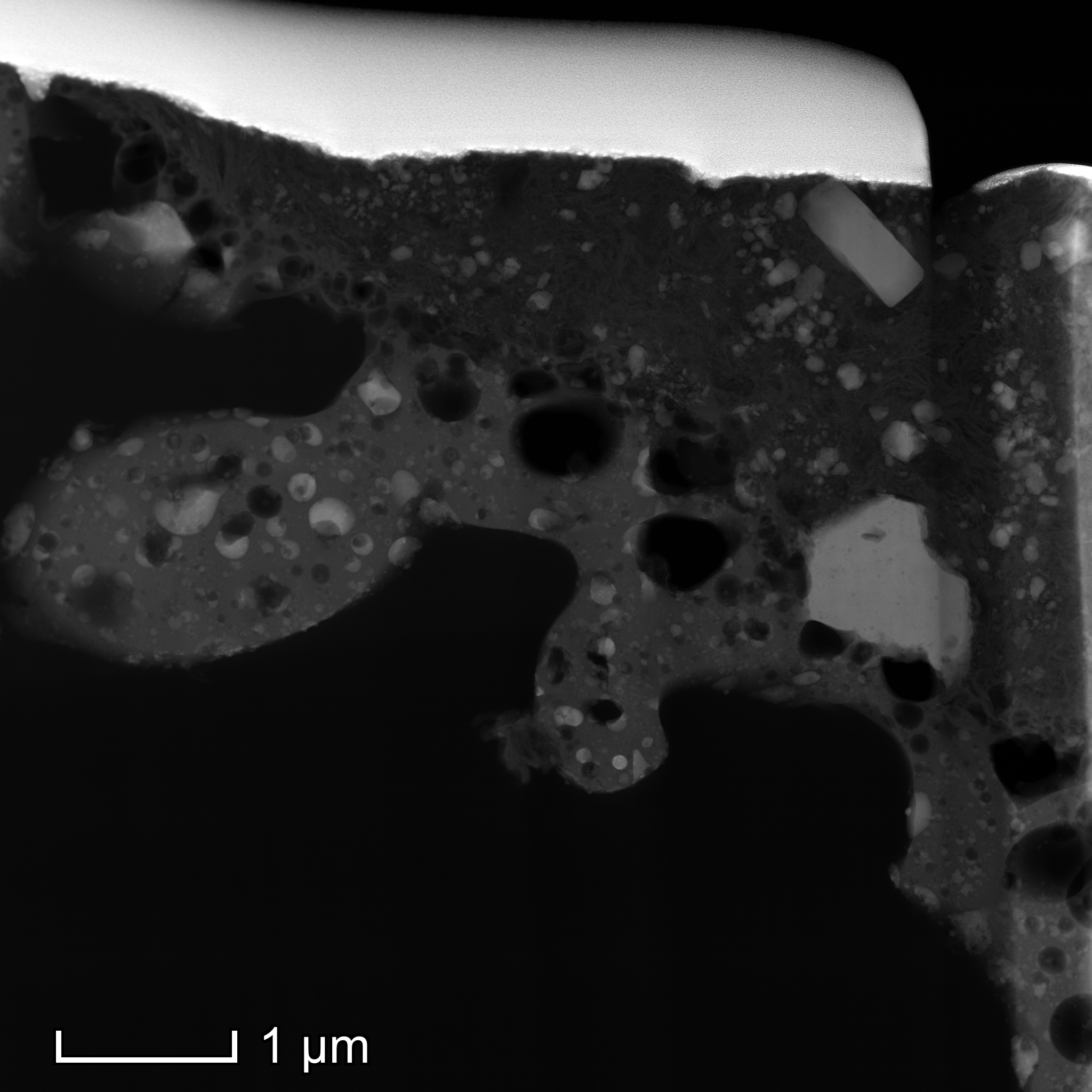

Supplement: Source Data Extended Data Fig. 3 — Unprocessed images that were used in Extended Data Fig. 3. [file 41550_2022_1841_MOESM8_ESM.zip › Source_Data_ED_Fig3/ED_Fig.3_03b.tif]

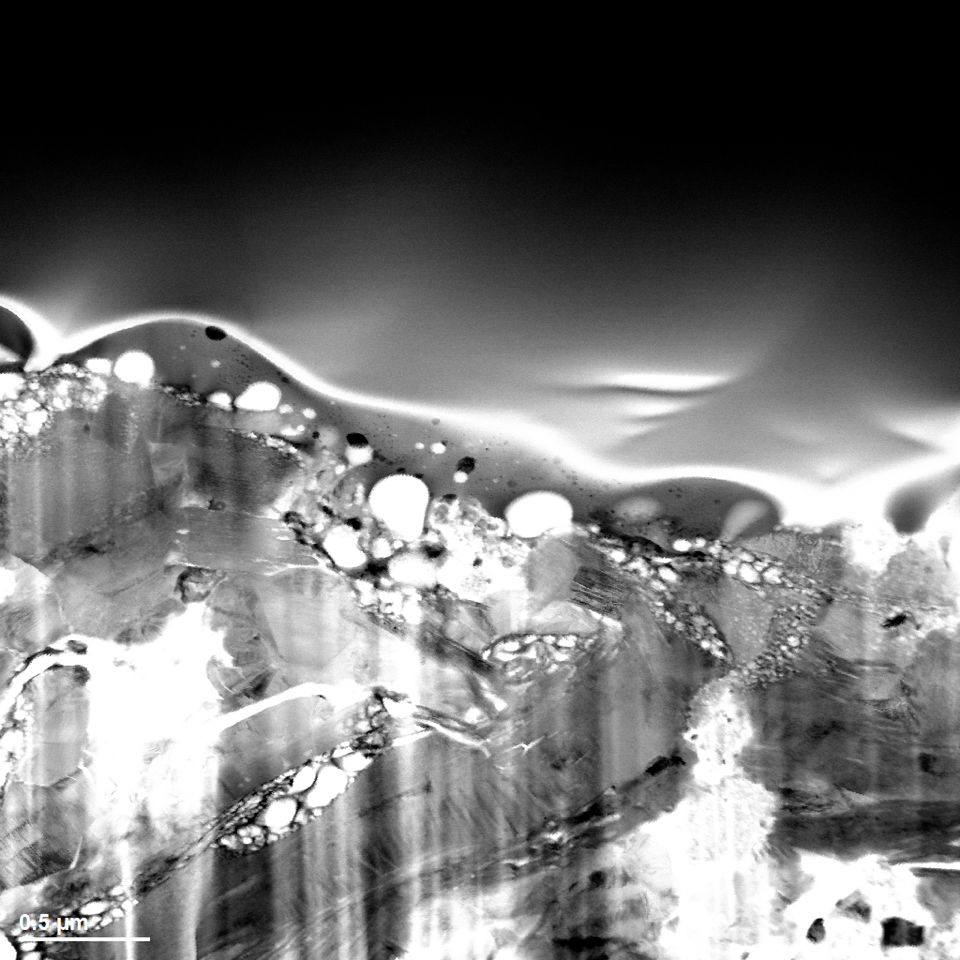

Supplement: Source Data Extended Data Fig. 3 — Unprocessed images that were used in Extended Data Fig. 3. [file 41550_2022_1841_MOESM8_ESM.zip › Source_Data_ED_Fig3/ED_Fig.3_04.tiff]

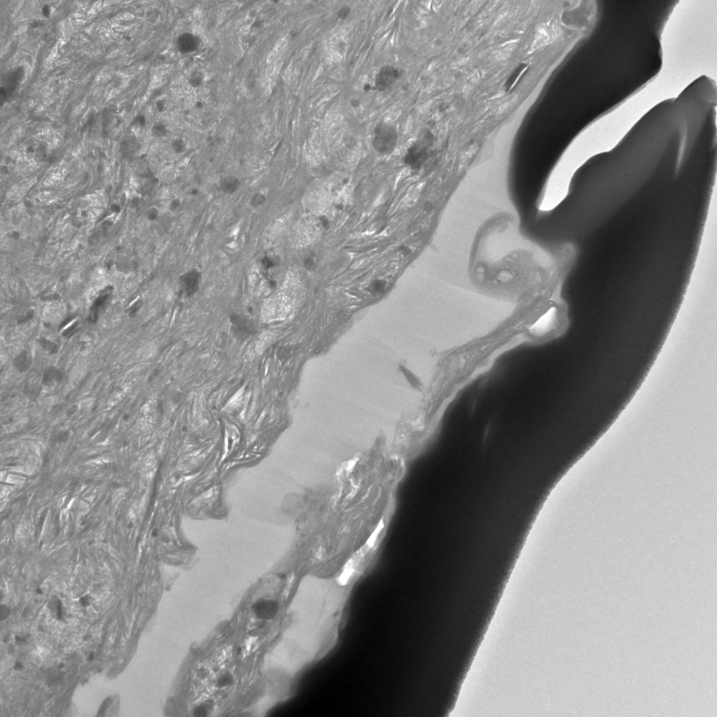

Supplement: Source Data Extended Data Fig. 4 — Unprocessed images that were used in Extended Data Fig. 4. [file 41550_2022_1841_MOESM9_ESM.zip › Source_Data_ED_Fig4/ED_Fig.4_02.tif]

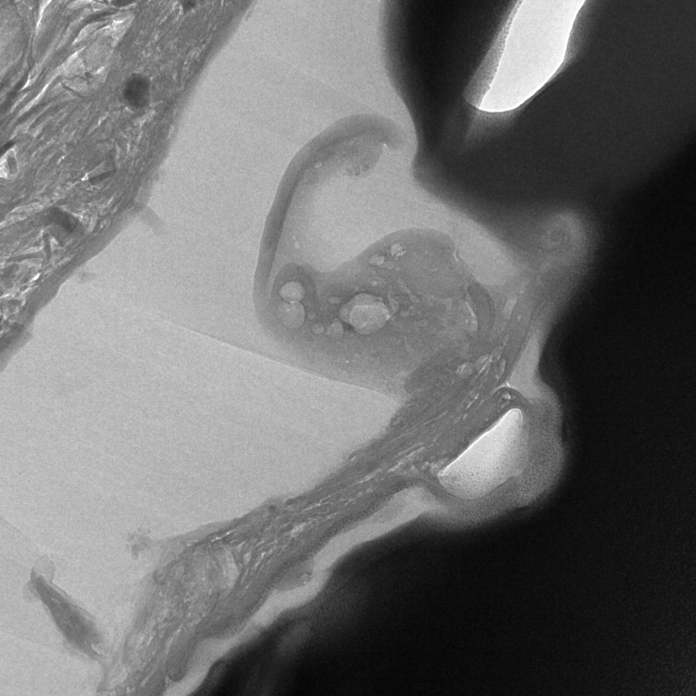

Supplement: Source Data Extended Data Fig. 4 — Unprocessed images that were used in Extended Data Fig. 4. [file 41550_2022_1841_MOESM9_ESM.zip › Source_Data_ED_Fig4/ED_Fig.4_03.tif]

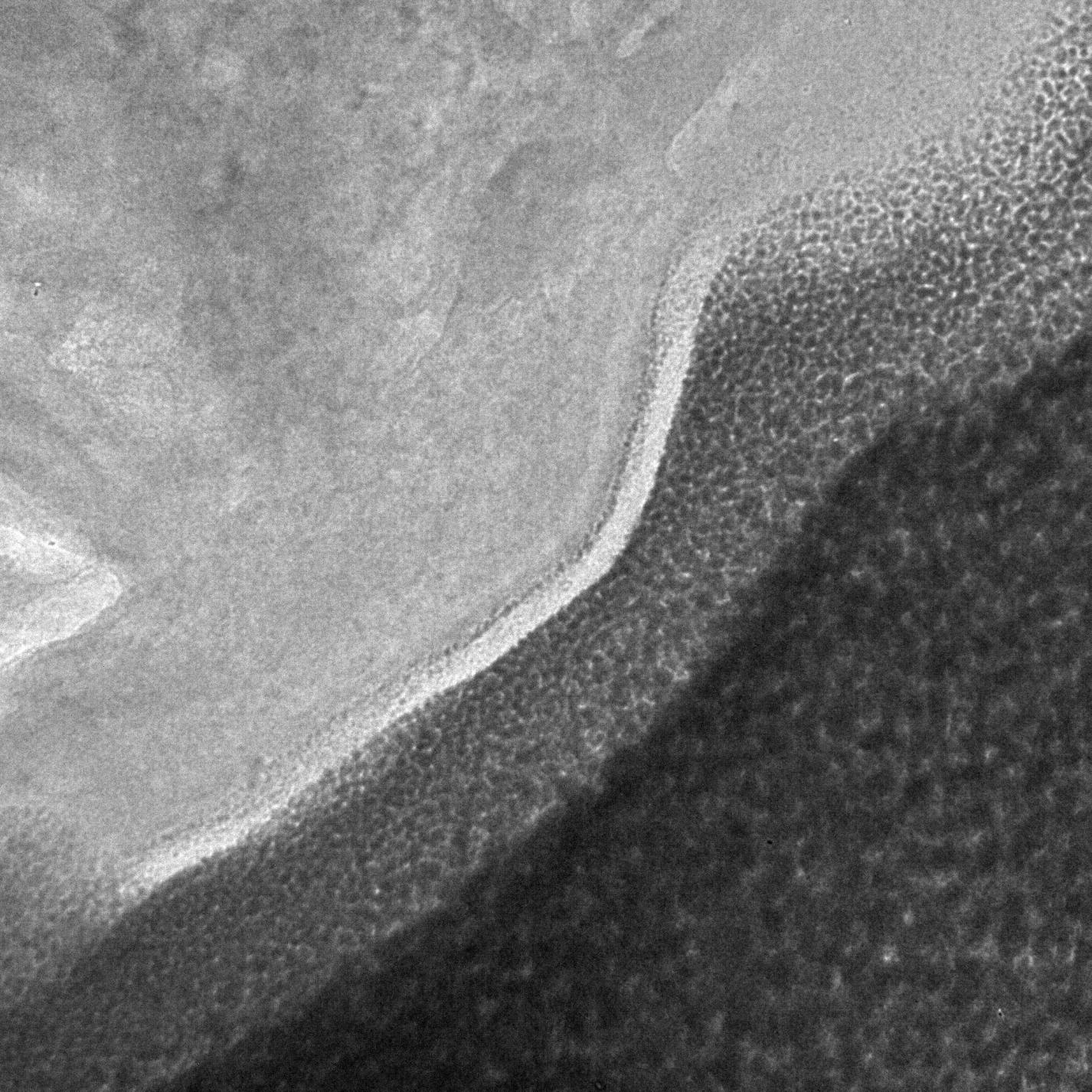

Supplement: Source Data Extended Data Fig. 5 — Unprocessed images that were used in Extended Data Fig. 5. [file 41550_2022_1841_MOESM10_ESM.zip › Source_Data_ED_Fig5/ED_Fig.5_01.jpg]

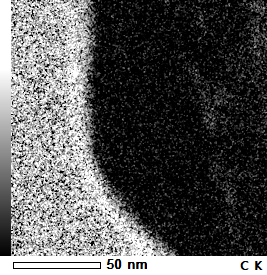

Supplement: Source Data Extended Data Fig. 5 — Unprocessed images that were used in Extended Data Fig. 5. [file 41550_2022_1841_MOESM10_ESM.zip › Source_Data_ED_Fig5/ED_Fig.5_02a.tif]

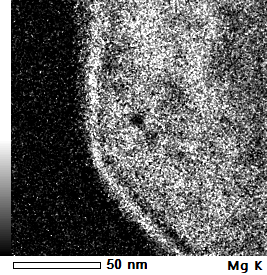

Supplement: Source Data Extended Data Fig. 5 — Unprocessed images that were used in Extended Data Fig. 5. [file 41550_2022_1841_MOESM10_ESM.zip › Source_Data_ED_Fig5/ED_Fig.5_02b.tif]

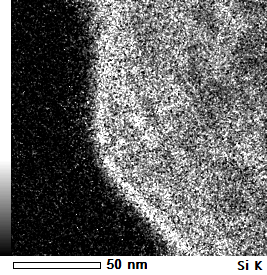

Supplement: Source Data Extended Data Fig. 5 — Unprocessed images that were used in Extended Data Fig. 5. [file 41550_2022_1841_MOESM10_ESM.zip › Source_Data_ED_Fig5/ED_Fig.5_02c.tif]

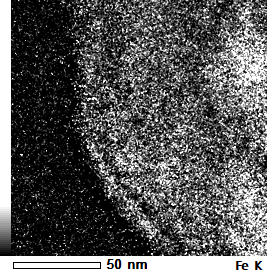

Supplement: Source Data Extended Data Fig. 5 — Unprocessed images that were used in Extended Data Fig. 5. [file 41550_2022_1841_MOESM10_ESM.zip › Source_Data_ED_Fig5/ED_Fig.5_02d.tif]

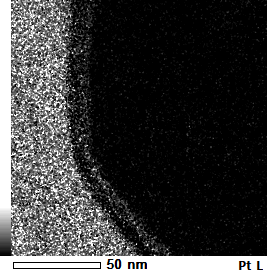

Supplement: Source Data Extended Data Fig. 5 — Unprocessed images that were used in Extended Data Fig. 5. [file 41550_2022_1841_MOESM10_ESM.zip › Source_Data_ED_Fig5/ED_Fig.5_02e.tif]

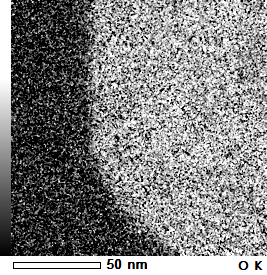

Supplement: Source Data Extended Data Fig. 5 — Unprocessed images that were used in Extended Data Fig. 5. [file 41550_2022_1841_MOESM10_ESM.zip › Source_Data_ED_Fig5/ED_Fig.5_02f.tif]

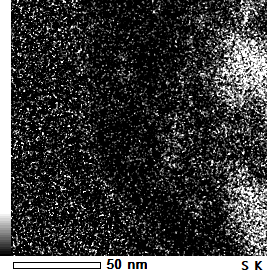

Supplement: Source Data Extended Data Fig. 5 — Unprocessed images that were used in Extended Data Fig. 5. [file 41550_2022_1841_MOESM10_ESM.zip › Source_Data_ED_Fig5/ED_Fig.5_02g.tif]

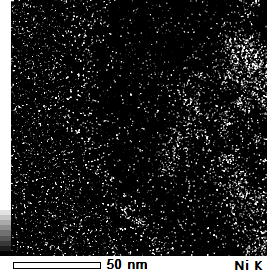

Supplement: Source Data Extended Data Fig. 5 — Unprocessed images that were used in Extended Data Fig. 5. [file 41550_2022_1841_MOESM10_ESM.zip › Source_Data_ED_Fig5/ED_Fig.5_02h.tif]

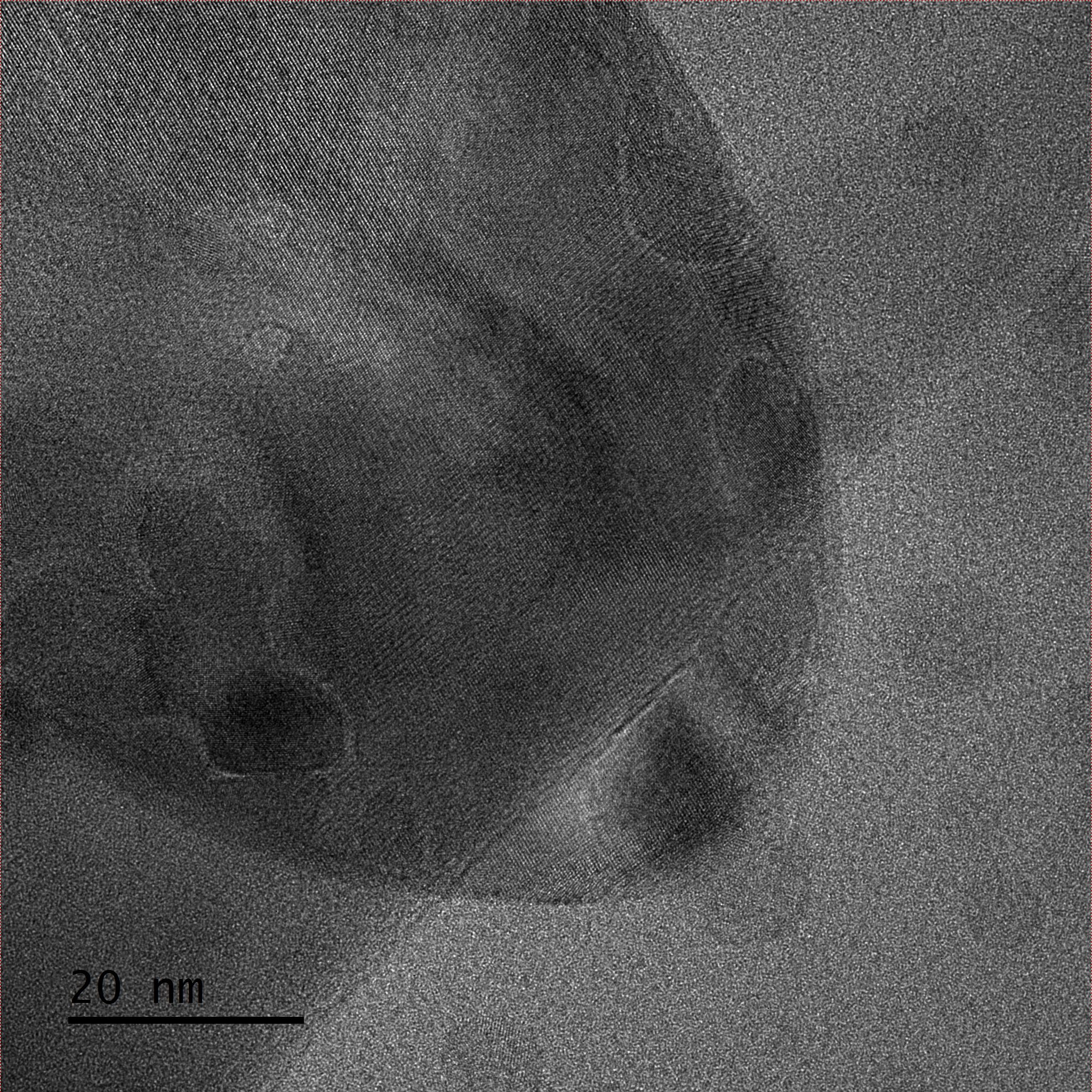

Supplement: Source Data Extended Data Fig. 6 — Unprocessed images that were used in Extended Data Fig. 6. [file 41550_2022_1841_MOESM11_ESM.zip › Source_Data_ED_Fig6/ED_Fig.6_01-03.tif]

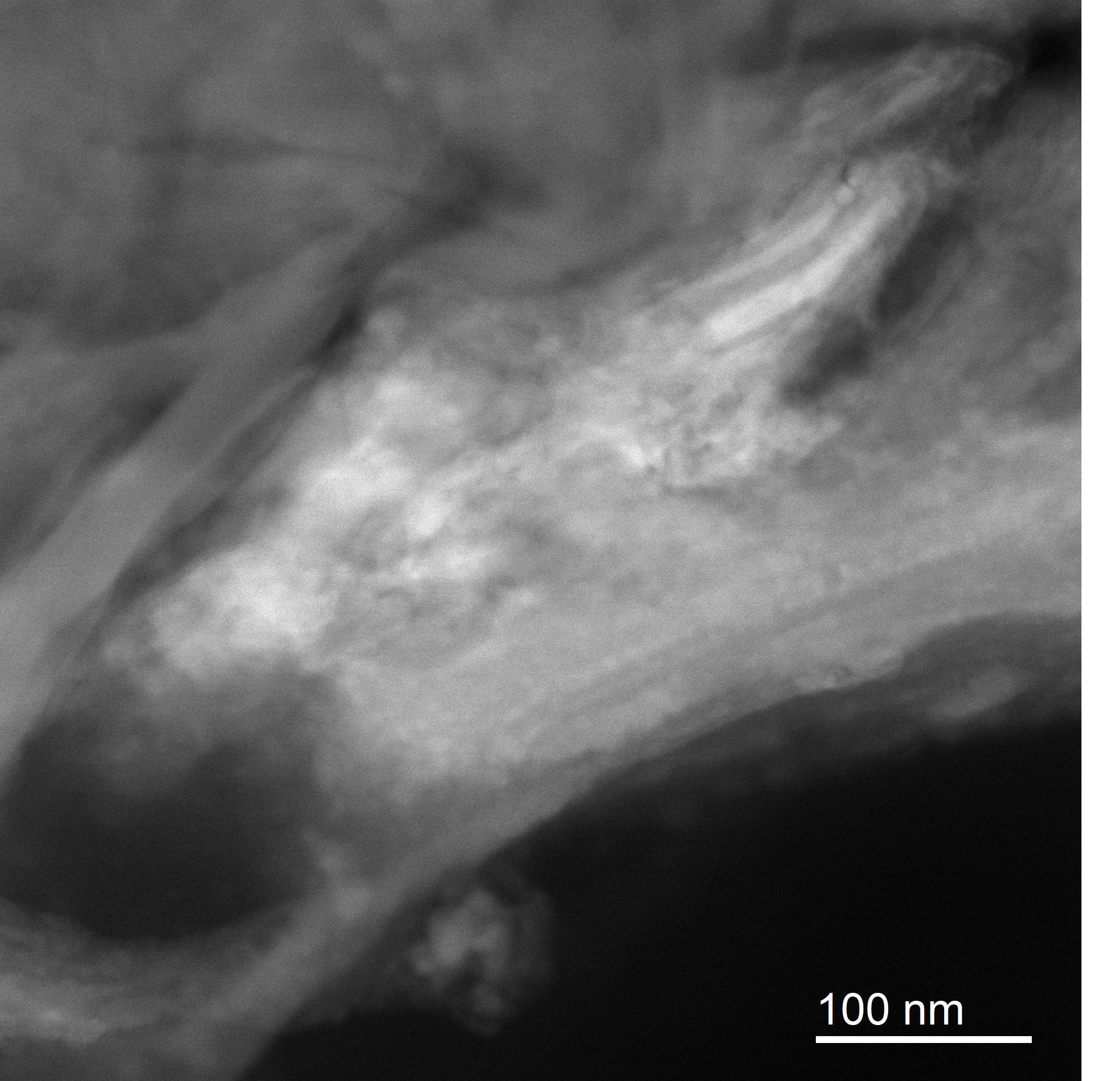

Supplement: Source Data Extended Data Fig. 6 — Unprocessed images that were used in Extended Data Fig. 6. [file 41550_2022_1841_MOESM11_ESM.zip › Source_Data_ED_Fig6/ED_Fig.6_04.jpg]

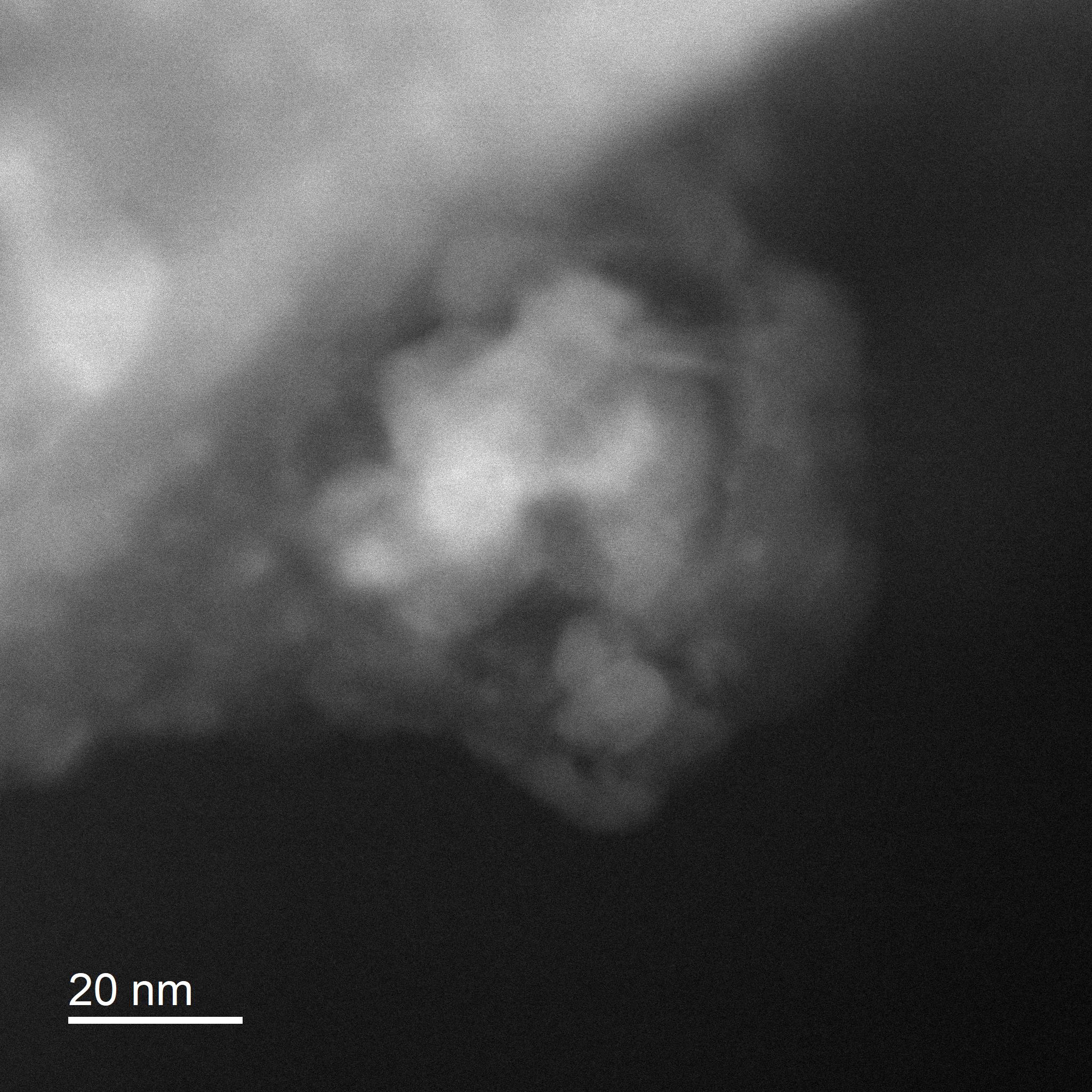

Supplement: Source Data Extended Data Fig. 6 — Unprocessed images that were used in Extended Data Fig. 6. [file 41550_2022_1841_MOESM11_ESM.zip › Source_Data_ED_Fig6/ED_Fig.6_05.jpg]

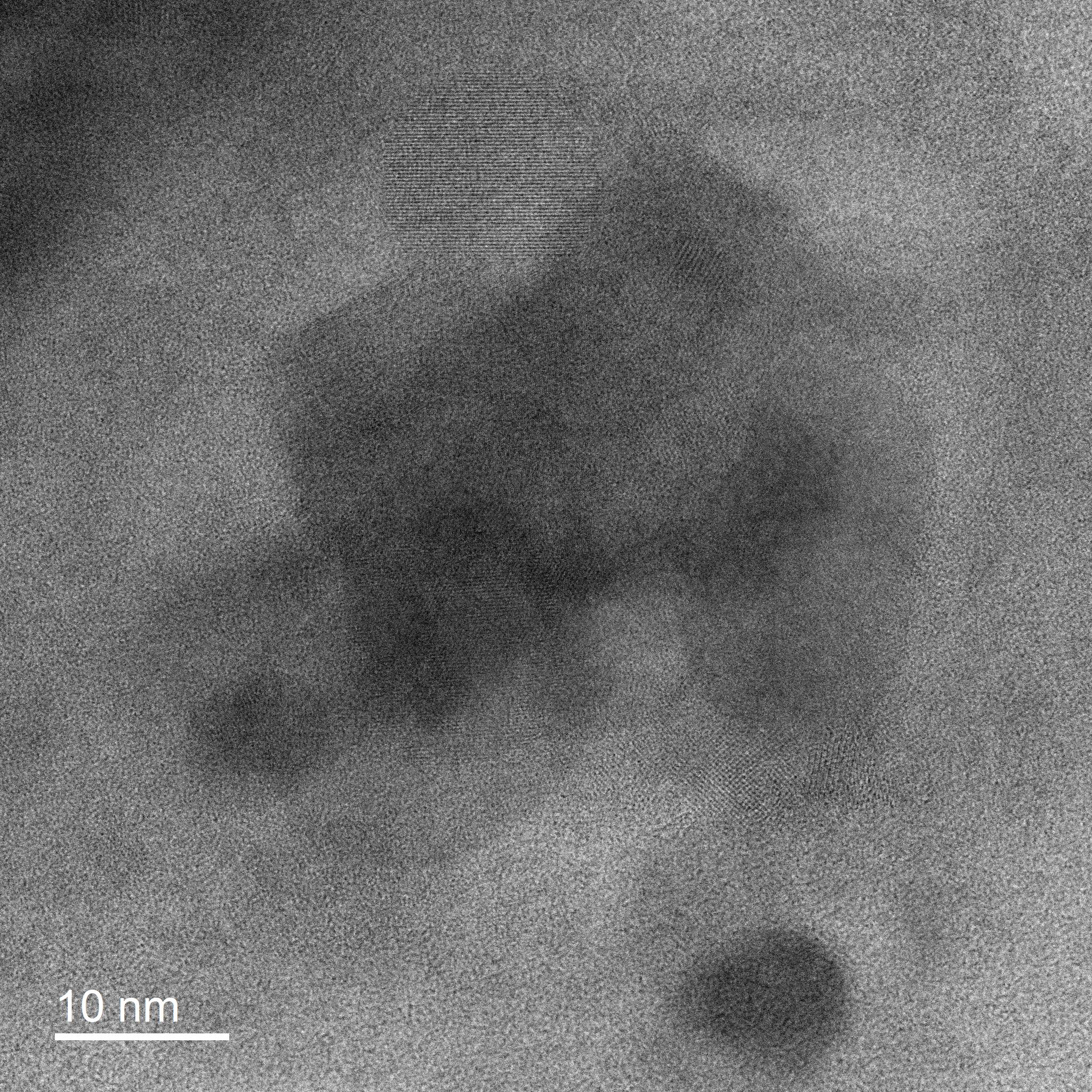

Supplement: Source Data Extended Data Fig. 6 — Unprocessed images that were used in Extended Data Fig. 6. [file 41550_2022_1841_MOESM11_ESM.zip › Source_Data_ED_Fig6/ED_Fig.6_06.jpg]

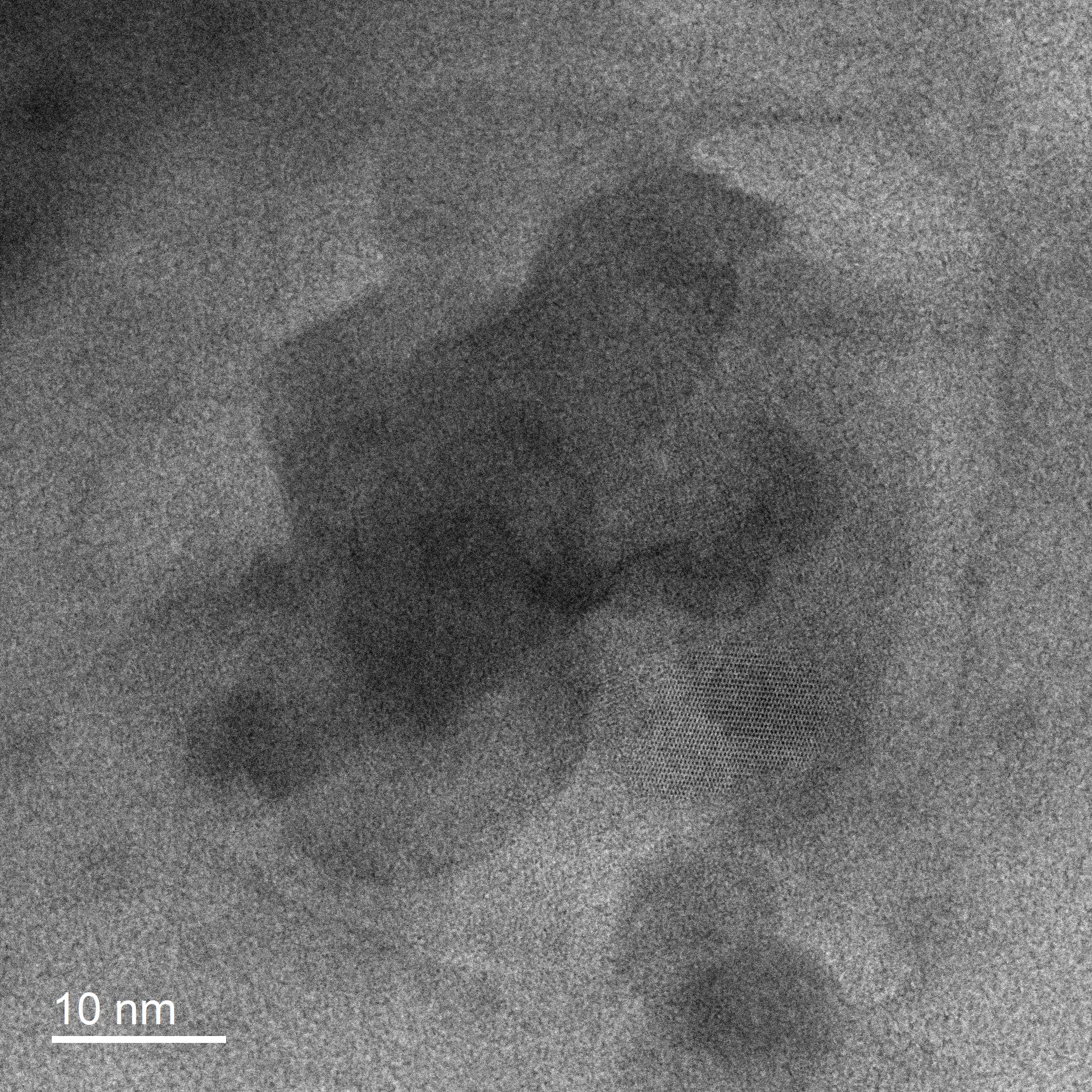

Supplement: Source Data Extended Data Fig. 6 — Unprocessed images that were used in Extended Data Fig. 6. [file 41550_2022_1841_MOESM11_ESM.zip › Source_Data_ED_Fig6/ED_Fig.6_07.jpg]

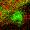

Supplement: Source Data Extended Data Fig. 6 — Unprocessed images that were used in Extended Data Fig. 6. [file 41550_2022_1841_MOESM11_ESM.zip › Source_Data_ED_Fig6/ED_Fig.6_08.jpg]

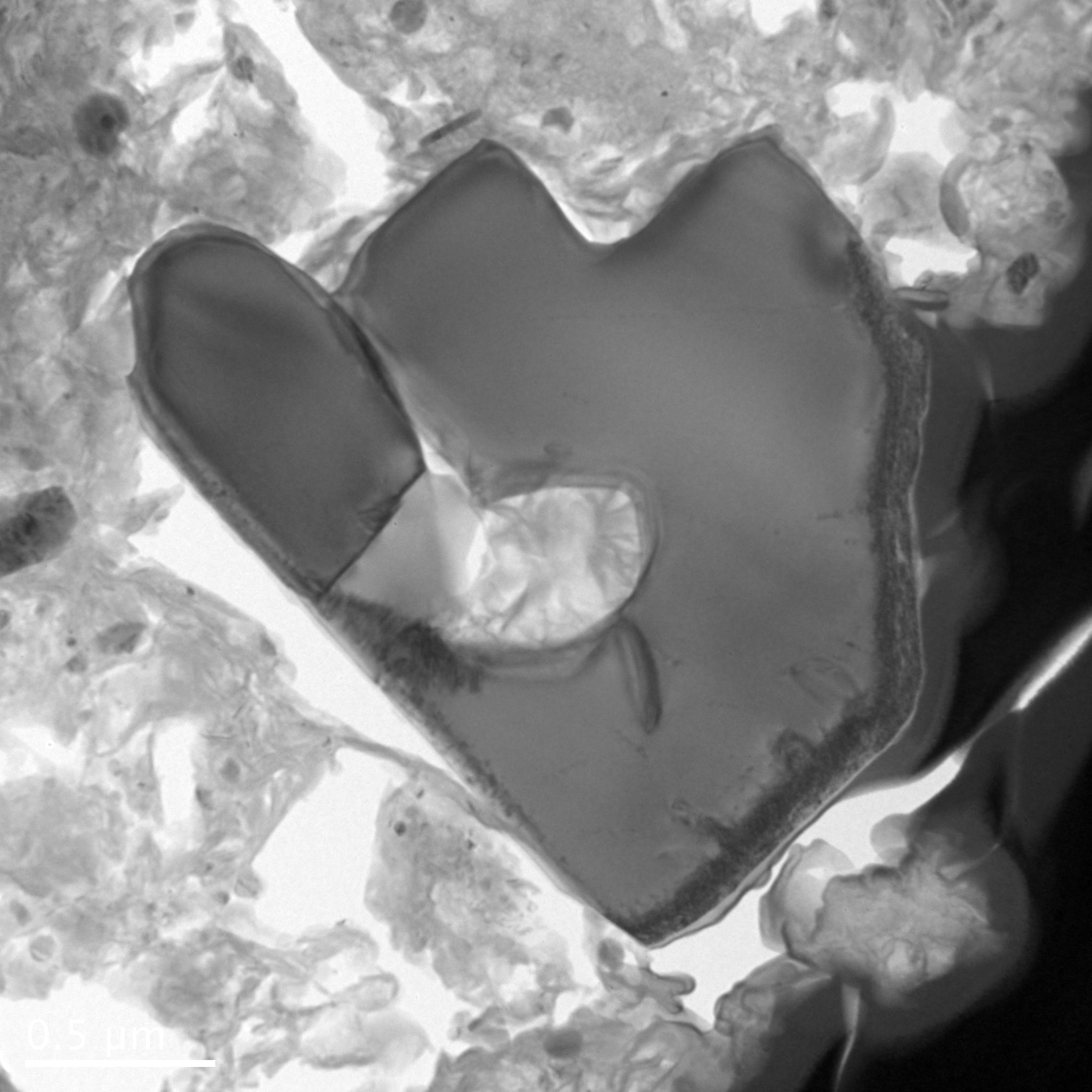

Supplement: Source Data Extended Data Fig. 7 — Unprocessed images that were used in Extended Data Fig. 7. [file 41550_2022_1841_MOESM12_ESM.zip › Source_Data_ED_Fig7/ED_Fig.7_01.jpg]

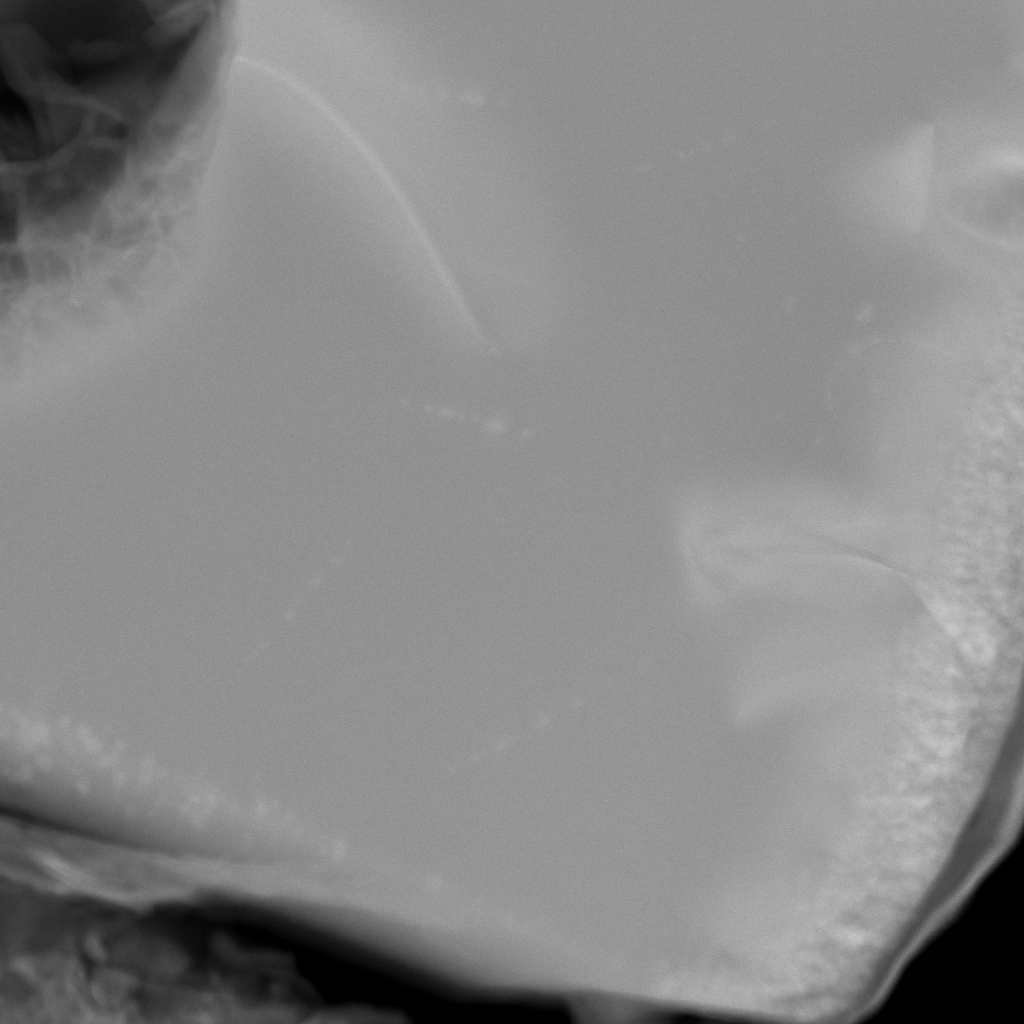

Supplement: Source Data Extended Data Fig. 7 — Unprocessed images that were used in Extended Data Fig. 7. [file 41550_2022_1841_MOESM12_ESM.zip › Source_Data_ED_Fig7/ED_Fig.7_02.tif]

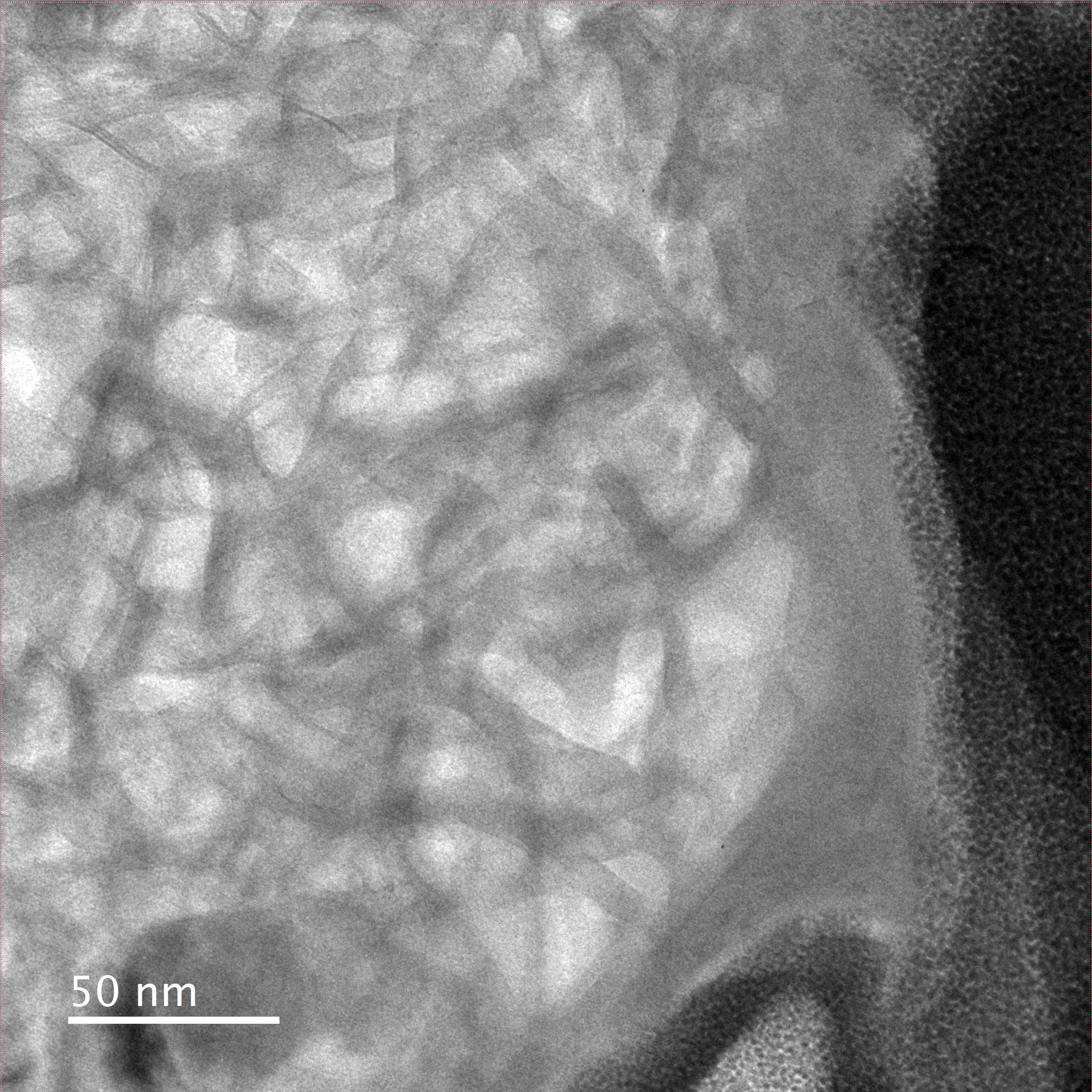

Supplement: Source Data Extended Data Fig. 7 — Unprocessed images that were used in Extended Data Fig. 7. [file 41550_2022_1841_MOESM12_ESM.zip › Source_Data_ED_Fig7/ED_Fig.7_03.jpg]

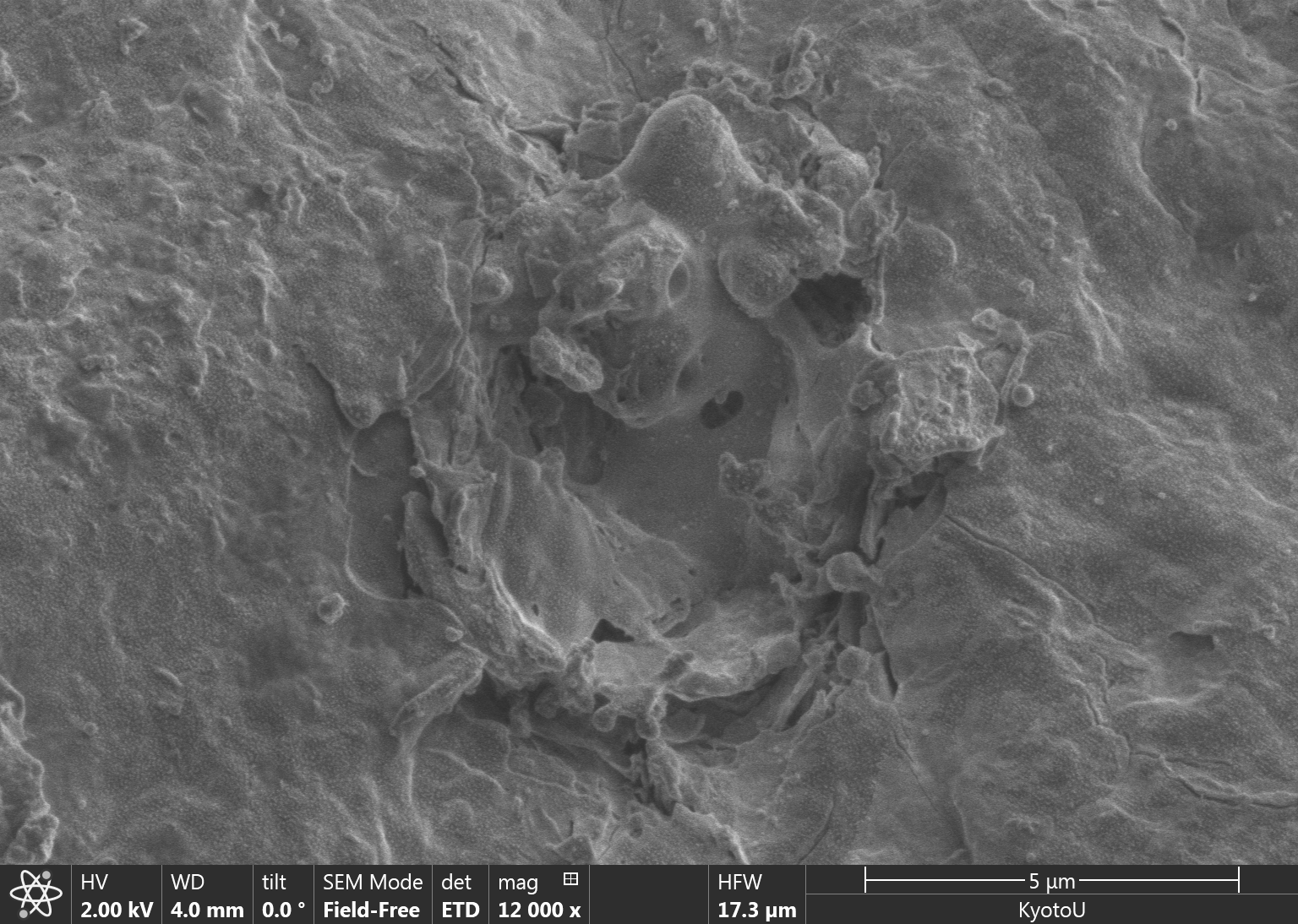

Supplement: Source Data Extended Data Fig. 8 — An unprocessed image and a graph that were used in Extended Data Fig. 8, and an excel file to make the graph. [file 41550_2022_1841_MOESM13_ESM.zip › Source_Data_ED_Fig8/ED_Fig.8_01.tif]

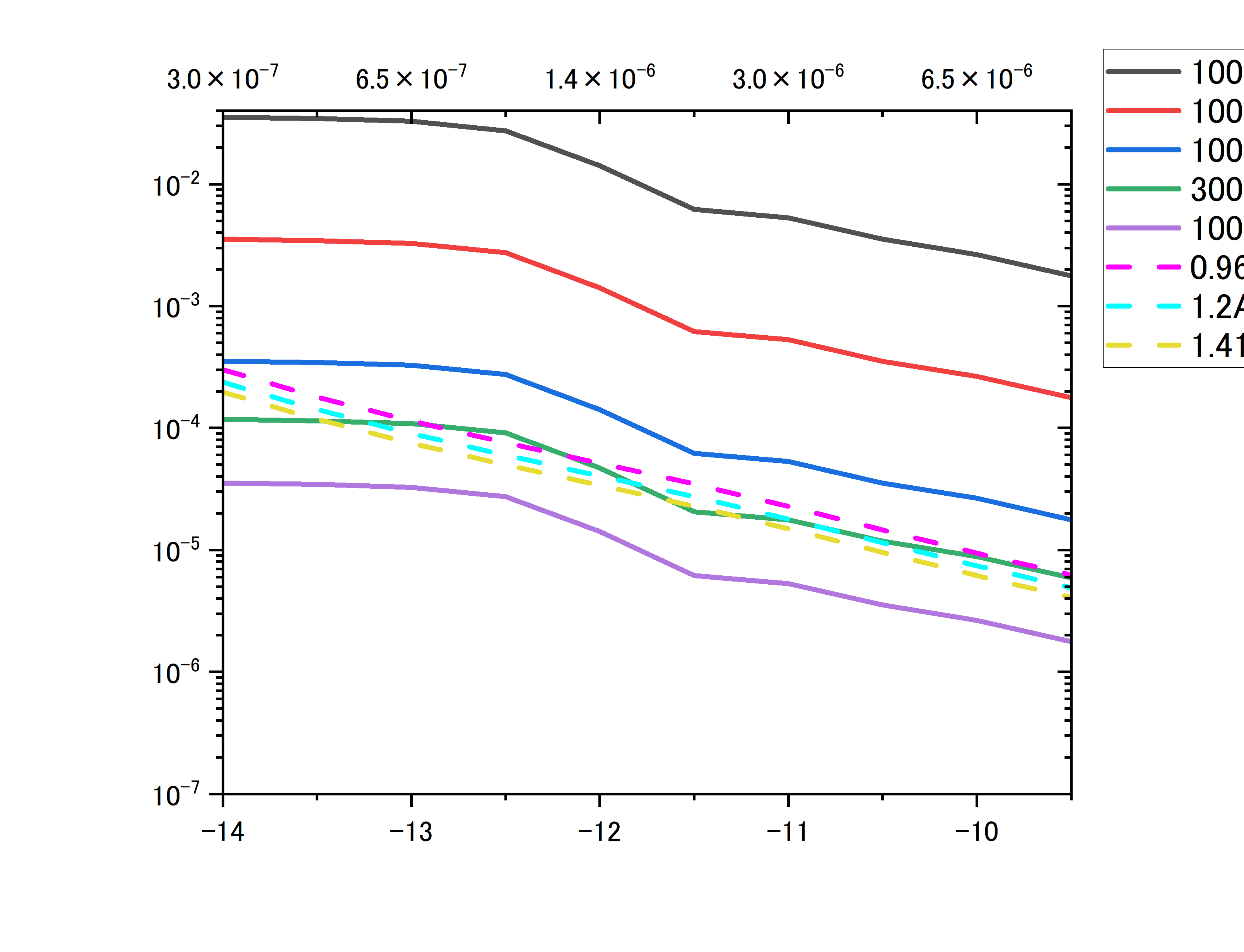

Supplement: Source Data Extended Data Fig. 8 — An unprocessed image and a graph that were used in Extended Data Fig. 8, and an excel file to make the graph. [file 41550_2022_1841_MOESM13_ESM.zip › Source_Data_ED_Fig8/ED_Fig.8_02.jpg]
